# Correction of oxidative stress enhances enzyme replacement therapy in Pompe disease

**DOI:** 10.15252/emmm.202114434

**Published:** 2021-10-04

**Authors:** Antonietta Tarallo, Carla Damiano, Sandra Strollo, Nadia Minopoli, Alessia Indrieri, Elena Polishchuk, Francesca Zappa, Edoardo Nusco, Simona Fecarotta, Caterina Porto, Marcella Coletta, Roberta Iacono, Marco Moracci, Roman Polishchuk, Diego Luis Medina, Paola Imbimbo, Daria Maria Monti, Maria Antonietta De Matteis, Giancarlo Parenti

**Affiliations:** ^1^ Telethon Institute of Genetics and Medicine Pozzuoli Italy; ^2^ Department of Translational Medical Sciences Federico II University Naples Italy; ^3^ Institute for Genetic and Biomedical Research (IRGB) National Research Council (CNR) Milan Italy; ^4^ Department of Biology University of Naples "Federico II", Complesso Universitario di Monte S. Angelo Naples Italy; ^5^ Institute of Biosciences and BioResources ‐ National Research Council of Italy Naples Italy; ^6^ Department of Chemical Sciences Federico II University Naples Italy; ^7^ Department of Molecular Medicine and Medical Biotechnologies Federico II University Naples Italy; ^8^ Present address: Department of Molecular, Cellular, and Developmental Biology University of California Santa Barbara CA USA; ^9^ Present address: IInd Division of Neurology Multiple Sclerosis Center University of Campania "Luigi Vanvitelli" Naples Italy

**Keywords:** alpha‐glucosidase, enzyme replacement therapy, N‐acetylcysteine, oxidative stress, Pompe disease, Genetics, Gene Therapy & Genetic Disease, Metabolism, Musculoskeletal System

## Abstract

Pompe disease is a metabolic myopathy due to acid alpha‐glucosidase deficiency. In addition to glycogen storage, secondary dysregulation of cellular functions, such as autophagy and oxidative stress, contributes to the disease pathophysiology. We have tested whether oxidative stress impacts on enzyme replacement therapy with recombinant human alpha‐glucosidase (rhGAA), currently the standard of care for Pompe disease patients, and whether correction of oxidative stress may be beneficial for rhGAA therapy. We found elevated oxidative stress levels in tissues from the Pompe disease murine model and in patients’ cells. In cells, stress levels inversely correlated with the ability of rhGAA to correct the enzymatic deficiency. Antioxidants (N‐acetylcysteine, idebenone, resveratrol, edaravone) improved alpha‐glucosidase activity in rhGAA‐treated cells, enhanced enzyme processing, and improved mannose‐6‐phosphate receptor localization. When co‐administered with rhGAA, antioxidants improved alpha‐glucosidase activity in tissues from the Pompe disease mouse model. These results indicate that oxidative stress impacts on the efficacy of enzyme replacement therapy in Pompe disease and that manipulation of secondary abnormalities may represent a strategy to improve the efficacy of therapies for this disorder.

## Graphical abstract

Enzyme replacement therapy (ERT) with recombinant human alpha‐glucosidase (rhGAA) is currently the standard of care for the treatment of Pompe disease. However, this approach shows important limitations. We have tested whether modulation of oxidative stress may improve the efficacy of ERT.

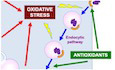

**The paper explained**

**Problem**

Pompe disease is a debilitating and progressive metabolic myopathy due to mutations of the *GAA* gene and functional deficiency of acid alpha‐glucosidase, an enzyme involved in the lysosomal breakdown of glycogen. Enzyme replacement therapy with recombinant human alpha‐glucosidase (rhGAA), currently the standard of care for Pompe disease patients, shows limitations. Despite treatment, some patients experience secondary decline after a few years, and some tissues, particularly skeletal muscles, appear to be relatively refractory to treatment. In addition to glycogen storage, secondary dysregulation of critical cellular pathways and functions contributes to the pathogenetic cascade of Pompe disease. We have tested whether these secondary abnormalities may represent novel therapeutic targets and whether modulation of these pathways is a potential adjunctive strategy to improve the efficacy of treatments for Pompe disease.

**Results**

We found elevated oxidative stress levels in tissues from the Pompe disease murine model and in patients’ cells. In cells, stress levels inversely correlated with the ability of rhGAA to correct the enzymatic deficiency. Correction of oxidative stress with antioxidants improved GAA activity in rhGAA‐treated cells with a synergistic effect. Similar results were observed in the Pompe disease mouse model treated with rhGAA and antioxidants.

**Impact**

The results of our studies point to secondary cellular abnormalities as key therapeutic targets in Pompe disease. We showed that factors related to recipient tissues, in addition to the intrinsic properties of the recombinant enzyme, influence the response to enzyme replacement therapy. Thus, manipulation of secondary abnormalities may represent a strategy to improve the efficacy of therapies for this disorder.

## Introduction

The pathology and the clinical manifestations of lysosomal storage diseases have been traditionally viewed as direct consequences of lysosomal engorgement with inert undegraded substrates. This concept has been challenged by the most recent vision of lysosomal functions. Together with the recognition of a central role of lysosomes in cellular homeostasis and metabolism (Ballabio & Bonifacino, 2020), multiple and diverse secondary events are now emerging as important players in the pathogenesis of these disorders. Among them, some have received particular attention and include autophagy impairment, jamming of intracellular vesicle trafficking, mitochondrial dysfunction, oxidative stress, abnormalities of calcium homeostasis, and dysregulation of signaling pathways (Platt *et al*, 2012; Parenti *et al*, 2021).

Pompe disease (PD) pathophysiology is a paradigm of the role of secondary events in lysosomal storage diseases. PD is due to mutations of the *GAA* gene and functional deficiency of acid alpha‐glucosidase, an enzyme involved in the lysosomal breakdown of glycogen (van der Ploeg & Reuser, 2008). PD phenotype is broad but is almost invariably highly debilitating and associated with premature death. Infantile‐onset patients, presenting within the first months of life, are affected by severe hypertrophic cardiomyopathy, skeletal myopathy, recurrent respiratory infections, and, if untreated, die within the first year of life. Late‐onset patients are affected by a progressive myopathy resulting in motor impairment, while heart involvement is usually asymptomatic (van der Ploeg & Laforet, 2016). Recent reports have further expanded PD phenotype with the demonstration of central nervous system involvement, particularly in severely affected patients (Korlimarla *et al*, 2019; Musumeci *et al*, 2019).

A typical pathological hallmark of PD is generalized intra‐lysosomal glycogen storage due to GAA deficiency. Several reports, however, have suggested that, in addition to glycogen storage, secondary dysregulation of critical cellular pathways and functions contributes to the pathogenetic cascade of the disease (Meena *et al*, 2020). Abnormalities of autophagy have been shown to play a primary role in the disease pathophysiology (Fukuda *et al*, 2006; Nascimbeni *et al*, 2012; Raben *et al*, 2012; Meena & Raben, 2020). Massive accumulation of autophagic material, due to upregulation of autophagy and/or impaired autophagic flux, has been mostly observed in skeletal muscles. A role of dysfunctional autophagy in PD pathophysiology has been further supported by studies based on modulation of autophagy by overexpression of either transcription factor EB (TFEB) or transcription factor binding to IGHM enhancer 3 (TFE3), two members of the MiT‐TFE family of transcription factors that control lysosomal biogenesis and activation of the autophagosomal–lysosomal pathway. In these studies, overexpression of either TFEB or TFE3 resulted in improved cellular clearance of glycogen *in vitro* (Spampanato *et al*, 2013; Martina *et al*, 2014) and in some amelioration of physical performance in the murine model of PD (Gatto *et al*, 2017). Other studies have also shown that additional factors, such as altered calcium signaling, mitochondrial dysfunction and oxidative stress also take part in the pathophysiology of PD (Lim *et al*, 2015).

The characterization of these secondary events has therapeutic implications. Currently, the only approved treatment for PD is enzyme replacement therapy (ERT) with recombinant human GAA (rhGAA) (Van den Hout *et al*, 2000; Van den Hout *et al*, 2004). Albeit effective on many aspects of PD, ERT has some limitations (Case *et al*, 2012; Kuperus *et al*, 2017; van der Meijden *et al*, 2018). A residual phenotype has been observed in infantile‐onset patients (Prater *et al*, 2012), while many late‐onset patients experience some secondary decline after a few years of therapy (Wyatt *et al*, 2012; Harlaar *et al*, 2019). There are several factors that affect ERT efficacy, such as the cross‐reactive immunological material status of patients (Kishnani *et al*, 2010; van Gelder *et al*, 2015), age at start of treatment (Chien *et al*, 2013), and advancement of disease progression (van der Meijden *et al*, 2015).

It is plausible that ERT limitations are in part due to the secondary cellular abnormalities triggered by storage. For example, in *in vitro* cellular systems and in the mouse model of PD rhGAA appears to be mistrafficked into areas of accumulation of autophagic material as a consequence of autophagy impairment and possibly of altered vesicle and membrane trafficking (Fukuda *et al*, 2006; Cardone *et al*, 2008; Spampanato *et al*, 2013). We speculated that secondary increase of oxidative stress is another factor limiting correction of GAA activity by ERT. Increased stress is expected to be one of the consequences of autophagy impairment and has been in fact observed in PD (Lim *et al*, 2015; Sato *et al*, 2017). The existence of a crosstalk between the autophagic pathway and oxidative stress has been clearly documented. On one hand, oxidative stress activates autophagy through reactive oxygen species (ROS) and reactive nitrogen species (RNS) that act as intracellular “alarm molecules” of cellular stress and of the availability of nutrients. This effect is mediated by mucolipin 1 (TRPML1) (Zhang *et al*, 2016) and mTOR complex 1 (mTORC1)‐independent activation of TFEB and TFE3 transcription (Martina & Puertollano, 2018). On the other hand, autophagy is required for removing the conditions that cause oxidative stress (such as starvation), for clearing ROS and RNS from cells, and ultimately to allow cells to cope with stress (Filomeni *et al*, 2015).

It has been shown that induction of oxidative stress in wild‐type astrocytes results into defective clathrin‐mediated endocytosis of transferrin (Volpert *et al*, 2017) and that in rat brain oxidative stress causes loss of soluble NSF (N‐ethylmaleimide‐sensitive factor) attachment protein receptor (SNARE) proteins (Kaneai *et al*, 2013) that are crucial for vesicle docking and fusion (Lang *et al*, 2001).

Thus, we have focused our attention on oxidative stress as a potential therapeutic target in PD. We have tested whether the ability of rhGAA in correcting GAA levels is affected by oxidative stress, and whether stress mitigation is a potential strategy to improve the lysosomal trafficking of the recombinant enzyme.

## Results

### Oxidative stress is increased in the Gaa KO mouse and in cells from PD patients

We evaluated the levels of oxidative stress in PD using standard biochemical tests that measure ROS levels (2′,7′‐dichloro‐dihydrofluorescein, DCFDA), lipid peroxidation (thiobarbituric acid reactive substances, TBARS), and intracellular glutathione (GSH) levels (5,5′‐dithiobis‐2‐nitrobenzoic acid, DTNB).

We first looked at tissues from a *Gaa* KO mouse, a murine model of PD generated by disruption of the *Gaa* gene exon 6 (Raben *et al*, 1998). We analyzed tissues that are most relevant for PD phenotype, specifically skeletal muscles (gastrocnemius, quadriceps, diaphragm), heart, and liver (Fig 1A). The results were compared to those obtained in the same tissues from wild‐type animals that were arbitrarily taken as equal to 100. In muscles and heart from the *Gaa* KO mouse, we found significantly increased ROS levels (*P* ranging between 0.0001 and 0.0451 in the different tissues) and lipid peroxidation (*P* ranging between 0.0012 and 0.0092), while in liver only lipid peroxidation was significantly increased (*P* = 0.0202). Western blot analysis of the stress marker p‐ERK (Fig 1B) in gastrocnemius also supported the presence of increased oxidative stress.

**Figure 1:**
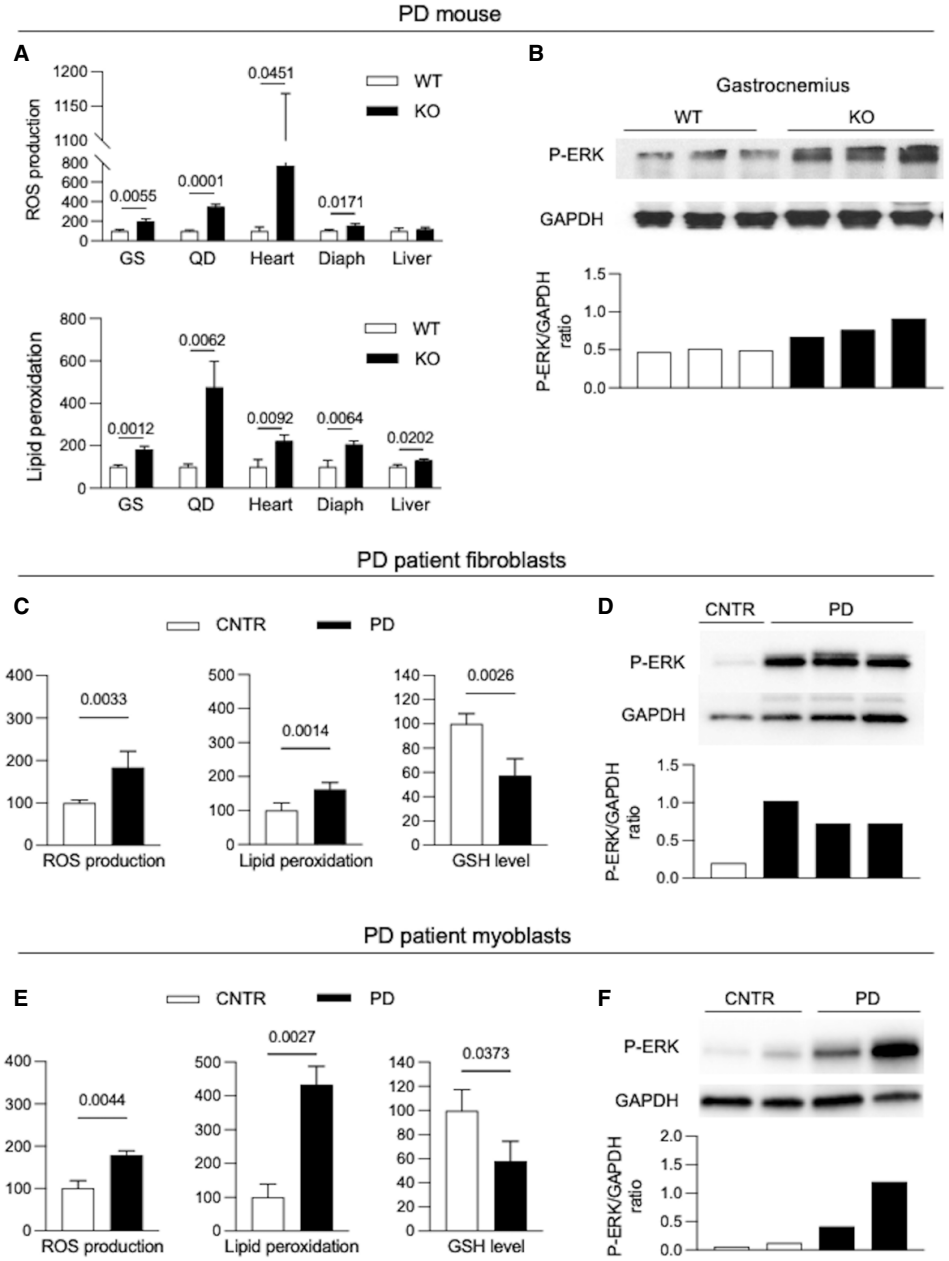
Increased oxidative stress in PD A, BOxidative stress biochemical markers (lipid peroxidation, ROS production) (A) and p‐ERK expression (B) in tissues from 3‐month‐old PD mice (KO) (*n* = 3) and from wild‐type (WT) mice.C, DOxidative stress biochemical markers (lipid peroxidation, ROS production, GSH levels) (C) and p‐ERK expression (D) in PD and control (CNTR) fibroblasts (*n* = 6).E, FOxidative stress biochemical markers (lipid peroxidation, ROS production, GSH levels) (E) and p‐ERK expression (F) in PD and control (CNTR) myoblasts (*n* = 4). Data information: In all instances, indicators of stress were increased in PD, compared to the respective control samples. Data information: In each experiment, at least biological triplicates were analyzed for each cell line or tissue sample; each assay was performed at least in duplicate. Data are presented as mean ± SD. Student’s *t*‐test was applied. Statistically significant comparison *P*‐values are indicated. Fig 1B: brightness +20%, contrast +41%. Source data are available online for this figure.

We performed the same biochemical tests in PD fibroblasts from patients with different phenotypes (classic infantile‐onset, intermediate, late‐onset) and in myoblasts from late‐onset patients (Table 1). The results were compared to those obtained in the respective control cell lines. Also in PD cells we found increased ROS levels (fibroblasts *P* = 0.0033; myoblasts *P* = 0.0044) and lipid peroxidation (fibroblasts *P* = 0.0014; myoblasts *P* = 0.0027), with reduced GSH levels (fibroblasts *P* = 0.0026; myoblasts *P* = 0.0373) and increased p‐ERK (Fig 1C–F). ROS levels and lipid peroxidation were lower in fibroblasts from late‐onset phenotypes (patients PD5, PD6) (Appendix Fig S1). The results obtained in cells were reflective of the *in vivo* findings, thus indicating that cultured cells, such as fibroblasts, are a reliable tool for further studies and for *in vitro* pharmacological manipulation of the oxidative stress pathway.

**Table 1:** Cell lines used in the study, patient genotypes, and phenotypes.

Pt ID	Genotype (DNA)	Genotype (protein)	Phenotype
PD1 fibroblasts	c.1124G>T / c.2261dupC	p.R375L / p.V755Sfs*41	Classic infantile
PD2 fibroblasts	c.1655T>C / c.1655T>C	p.L552P / p.L5552P	Intermediate
PD3 fibroblasts	c.1655T>C / c.236_246del	p.L552P / p.P79Rfs*12	Classic infantile
PD4 fibroblasts	c.1101G>A / c.1927G>A	p.W367X / p.G643R	Classic infantile
PD5 fibroblasts	c.1655T>C / c.‐35C>A	p.L552P / abnormal splicing	Intermediate / late onset
PD6 fibroblasts	c.‐32‐13T>G / unknown	abnormal splicing / unknown	Late onset
PD7 myoblasts	c.‐32‐13T>G / c.1551+1G>C	abnormal splicing / abnormal splicing	Late onset
PD8 myoblasts	c.‐32‐13T>G / c.525delT	abnormal splicing / p.T175fsX46	Late onset
PD9 myoblasts	c.‐32‐13T>G / c.989G>A	abnormal splicing / p.W330*	Late onset
PD10 myoblasts	c.‐32‐13T>G /c.1927G>A	abnormal splicing / p.G643R	Late onset
PD11 myoblasts	c.‐32‐13T>G / c.2237G>A	abnormal splicing / p.W746X	Late onset

In several disease conditions (Pieczenik & Neustadt, 2007; Stepien *et al*, 2017), mitochondrial dysfunction contributes to the increased oxidative stress. Indeed, an impairment of mitochondrial function and calcium signaling has also been extensively documented in PD (Lim *et al*, 2015; Sato *et al*, 2017; Meena & Raben, 2020).

In line with these studies, we found abnormal mitochondrial morphology in the *Gaa* KO mouse. In gastrocnemii, in addition to the typical PD hallmarks (intra‐lysosomal glycogen storage, expansion of the autophagic compartment), ultrastructural analysis showed abnormal mitochondria with altered cristae and electron‐lucent matrix (Fig EV1A). A quantitative analysis of the number and morphology of mitochondria in 15 low‐magnification (16,500×) electron microscopy fields showed that the number of abnormal mitochondria was significantly higher (*P* = 0.0038) in *Gaa* KO mice than in wild‐type animals (Fig EV1B and C). In homogenates from *Gaa* KO gastrocnemii, we also performed a Western blot analysis of components of the 5 oxidative phosphorylation (OXPHOS) complexes (Uqcrc2, ATP5A, mtCOI, Sdhb, and Ndufb8) and of the mitochondrial outer membrane (Mfn2) (Fig EV1D). In the *Gaa* KO gastrocnemii, these markers were increased, as compared to control tissues, indicating an accumulation of mitochondrial proteins.

**Figure EV1:**
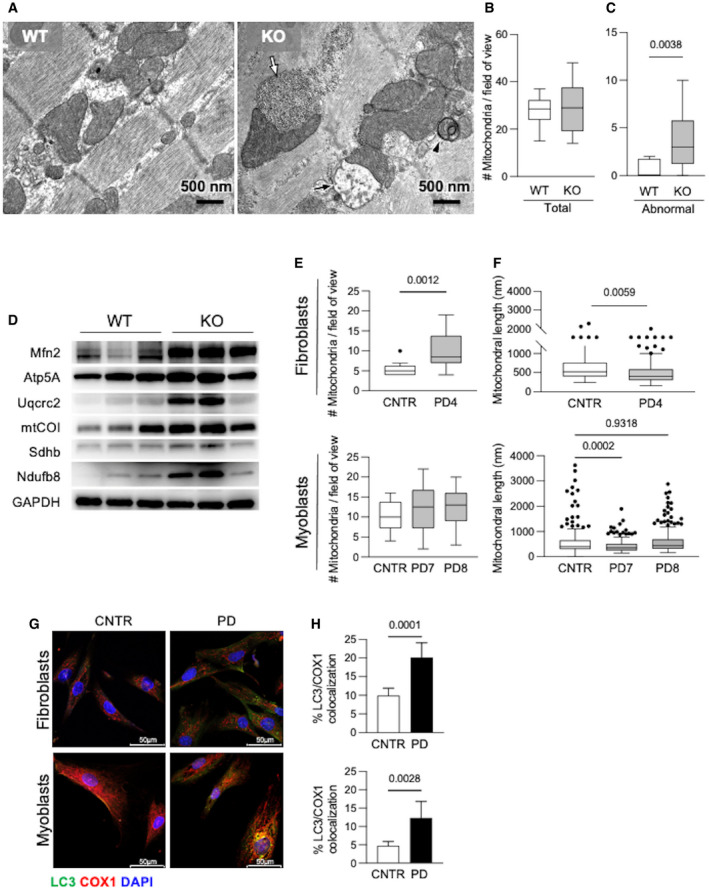
Characterization of mitochondria in gastrocnemii from the PD mouse model and in cultured PD patient cells AUltrastructural analysis of gastrocnemii from the *Gaa* KO mouse showed intra‐lysosomal glycogen storage (white arrow), active mitophagy (arrowhead), abnormal mitochondria (black arrow).B, CQuantitative analysis of the number of mitochondria (B) and of morphologically abnormal mitochondria (C) in 15 low‐magnification (16×) electron microscopy fields showing significantly increased number of abnormal mitochondria (*P* = 0.0038) in *Gaa* KO compared to wild‐type animals. Data presented as mean ± SD of at least 12 fields for each mouse muscle. Student’s *t*‐test was applied.DWestern blot analysis of the levels of OXPHOS complexes in mitochondrial preparations from the Gaa KO gastrocnemii, showing increased levels of the markers tested.E, FNumber of mitochondria (E) and mitochondrial length (F) in PD fibroblasts and myoblasts compared to, respectively, control cells. Data presented as mean ± SD of at least 15 fields for each cell line. A Student’s *t*‐test was applied.G, HCo‐staining of COX1 with LC3 (G) and quantitative analysis (H) showing significantly increased colocalization of these markers in PD cells compared to control cells under standard culture conditions. Data presented as mean ± SD of five images for each cell line. A Student’s *t*‐test was applied. Confocal 63× images; scale bar 50 µm; contrast +15%; brightness +25%. Data information: In B, E, F, boxes include values between upper and lower quartiles, and central band corresponds to median, whiskers, and lower extremes to higher and lower values. Outlier values are indicated as dots. Source data are available online for this figure.

In PD fibroblasts and myoblasts, ultrastructural abnormalities of mitochondria were also present, although less pronounced (Fig EV1E and F).

In PD fibroblasts, co‐staining of cytochrome c oxidase1 (COX1) with the autophagy marker microtubule‐associated protein 1A/1B‐light chain 3 (LC3) showed significantly increased colocalization of these markers in PD cells compared to control cells under standard culture conditions (Fig EV1G and H). These data (in combination with signs of activated autophagy) (Fig EV2A and B) are compatible with active but less efficient mitophagy, with reduced progression of aged or damaged mitochondria to the terminal part of autophagosomal–lysosomal pathway.

**Figure EV2:**
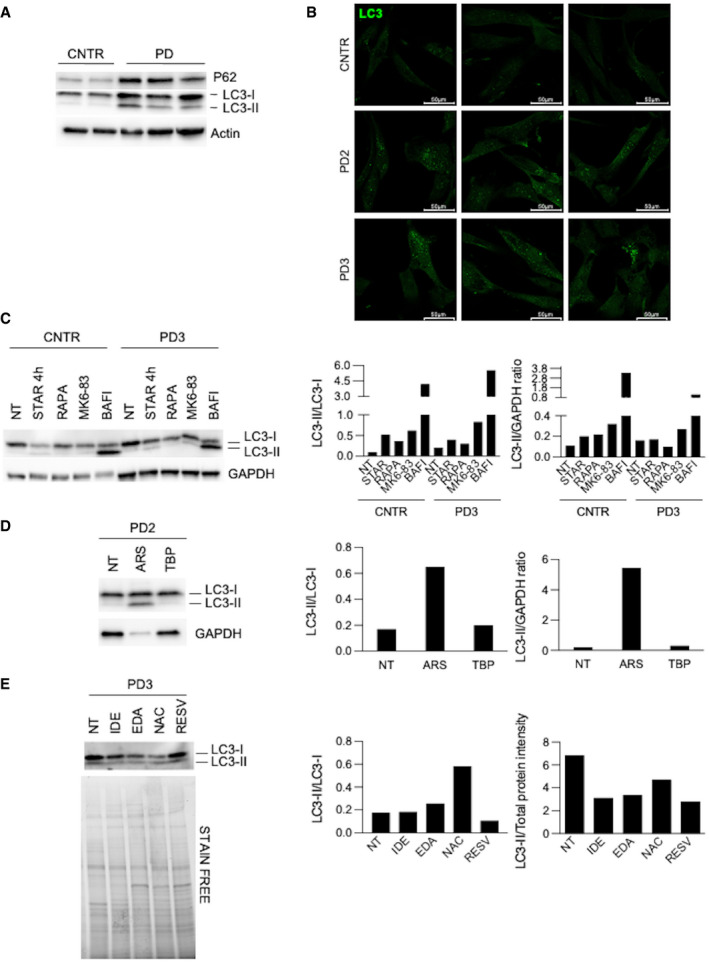
Autophagy markers Western blot analysis of p62 and LC3 in control (CNTR) (*n* = 2) and Pompe disease (PD) (*n* = 3) fibroblasts.Immunofluorescence analysis of LC3 in cultured CNTR and PD fibroblasts. Confocal 63× images; scale bar 50 µm; brightness +25%; contrast +20%.Western blot and quantitative analyses of autophagy marker LC3 in CNTR and in a PD cell line. The analysis was performed in untreated cells and after different treatments to modulate autophagy (starvation, STAR; rapamycin, RAPA; MK6‐83; bafilomycin, BAFI).Western blot and quantitative analyses of autophagy marker LC3 in a PD cell line. The analysis was performed in untreated cells and after different treatments to induce stress (sodium arsenite, ARS; tert‐butyl‐peroxide, TBP).Western blot and quantitative analyses of autophagy marker LC3 in a PD cell line. The analysis was performed in untreated cells and after antioxidant treatments. Source data are available online for this figure.

### Modulation of autophagy impacts on stress in fibroblasts

The increase of oxidative stress is expected to be a consequence of the impairment of autophagy typically observed in PD and of mitochondrial dysfunction. To support this hypothesis, we evaluated the effects of physiological or pharmacological activation of autophagy on oxidative stress levels in PD fibroblasts. As previously observed (Cardone *et al*, 2008), PD fibroblasts from infantile‐onset patients show signs of impaired autophagy, with increased LC3 and p62 levels and accumulation of LC3‐positive vesicles (Fig EV2A and B).

To stimulate autophagy, we used starvation that physiologically activates autophagy (Klionsky & Emr, 2000; Reggiori & Klionsky, 2002; Levine & Klionsky, 2004; He *et al*, 2018), rapamycin, a known inhibitor of the mTOR complex (Ballou & Lin, 2008; Lamming, 2016), and MK6‐83, a known activator of the cation‐permeable channel TRPML1 (Shen *et al*, 2012; Chen *et al*, 2014; Wang *et al*, 2015), while to block of autophagy we treated cells with the vacuolar ATPase inhibitor bafilomycin.

As expected, starvation, rapamycin, and MK6‐83 induced a relative increase of LC3‐II on Western blot analysis and increased LC3‐II to LC3‐I ratio (Figs 2A and B, and EV2C), indicating activation of autophagy. Bafilomycin induced remarkable accumulation of LC3‐II (Figs 2A and B, and EV2C), consistent with further impairment of the autophagic pathway and accumulation of undegraded LC3‐II.

**Figure 2:**
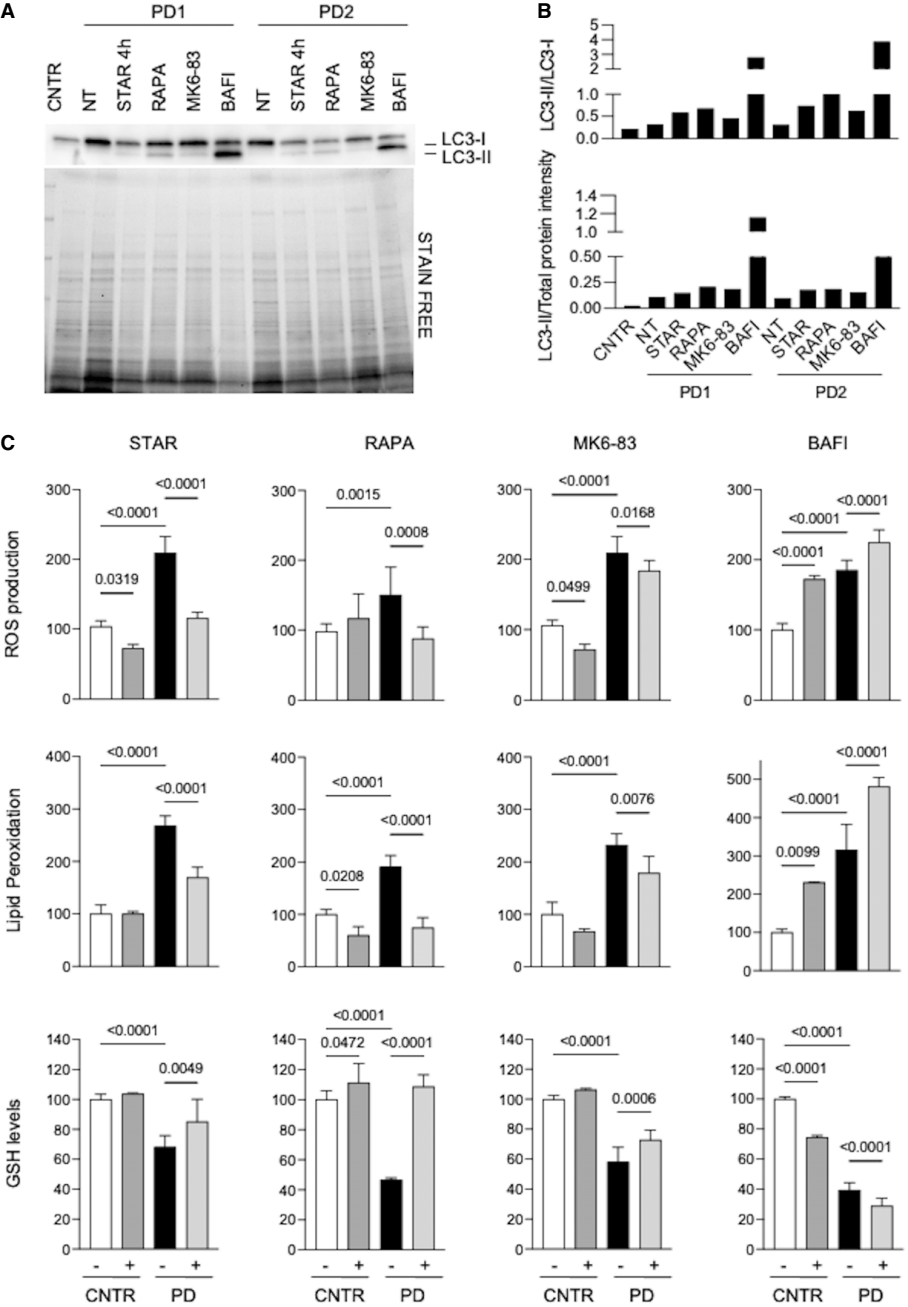
Modulation of autophagy impacts on stress in PD fibroblasts A, BWestern blot analysis of the autophagy marker LC3 (A) and quantitative analyses (B) in untreated control fibroblasts (CNTR) and in 2 PD fibroblasts cell lines (PD1, PD2). PD cells were untreated (NT) or subjected to different treatments to modulate autophagy (starvation, STAR; rapamycin, RAPA; MK6‐83; bafilomycin, BAFI). Results obtained in a third cell line (PD3) and in a control fibroblast cell line subjected to the same autophagy‐modulating treatments are shown in Fig EV2.CROS production, lipid peroxidation, and GSH levels in PD fibroblasts (*n* = 3). CNTR (white bars), CNTR after treatment (dark gray bars), PD samples (black bars), and PD after treatment (light gray bars). Physiological or pharmacological enhancement of autophagy resulted in correction of oxidative stress in PD cells, while bafilomycin treatment further increased stress in CNTR and PD cells. Data information: Starvation of cells was performed for 4 h; treatments with 20 µM rapamycin or 30 µM MK6‐83 or 100 nM bafilomycin was performed for 24 h. Data presented as mean ± SD. *P*‐values were calculated with ANOVA followed by Sidak's multiple comparison test. Statistically significant comparison *P*‐values are indicated. Source data are available online for this figure.

In PD, fibroblasts activation of autophagy by starvation, rapamycin, and MK6‐83 resulted into a significant reduction of oxidative stress, with decreased ROS production and lipid peroxidation, and with increased GSH levels, compared to cells cultured under standard conditions (Fig 2C). In contrast, treatment with bafilomycin, further increased ROS production and lipid peroxidation, and decreased GSH level (Fig 2C, right). In control fibroblasts, in which baseline levels of the stress indicators are normal and autophagy is functional, the use of autophagy‐stimulating conditions had lesser effects compared to PD cells, while bafilomycin‐mediated block of autophagy induced significant changes, with substantial increases of ROS and lipid peroxidation, and reduction of GSH. The effects of starvation on ROS production were time‐dependent, with an initial decrease in the first 4 h (likely mediated by autophagy induction) and progressive increase of stress up to 24 h (Appendix Fig S2). This pattern is consistent with the notion that starvation is an autophagy inducer, but on the long‐term increases ROS production and cellular stress (Scherz‐Shouval *et al*, 2007; Filomeni *et al*, 2010).

The combination of these results and of literature data (Spampanato *et al*, 2013; Myerowitz *et al*, 2021) suggests that in PD the autophagic pathway is impaired, but not totally blocked. Even if upregulated, this pathway is still sensitive to pharmacological or physiological stimuli and can be further manipulated and activated. While substrates (such as glycogen) cannot be degraded in lysosomes, due the enzymatic defect, ROS can be better disposed of upon stimulation of autophagy.

### Stress impacts on correction of GAA activity by rhGAA

We speculated that oxidative stress affects the level of correction of GAA activity by rhGAA. This hypothesis is supported by previous studies, showing an impairment of clathrin‐mediated endocytosis after induction of oxidative stress, as a result of down‐regulation of the transcription factor Sp1 and decreased levels of Hsc70, a protein involved in clathrin uncoating (Volpert *et al*, 2017).

We treated 6 PD fibroblasts cell lines with alglucosidase‐alpha, an rhGAA preparation that is currently approved for the treatment of PD patients, according to previously published procedures (Porto *et al*, 2009), and looked for possible correlations with the stress indicators used in previous experiments. We found that ROS levels and lipid peroxidation inversely correlated with correction of GAA activity in rhGAA‐treated cells (Fig 3A and B), with less efficient correction in cells showing the highest levels of stress, whereas no significant correlation was found between GSH levels and correction of GAA activity (Fig 3C). Cells derived from late‐onset patients showed lower stress levels (Appendix Fig S1) and higher levels of GAA activity correction.

**Figure 3:**
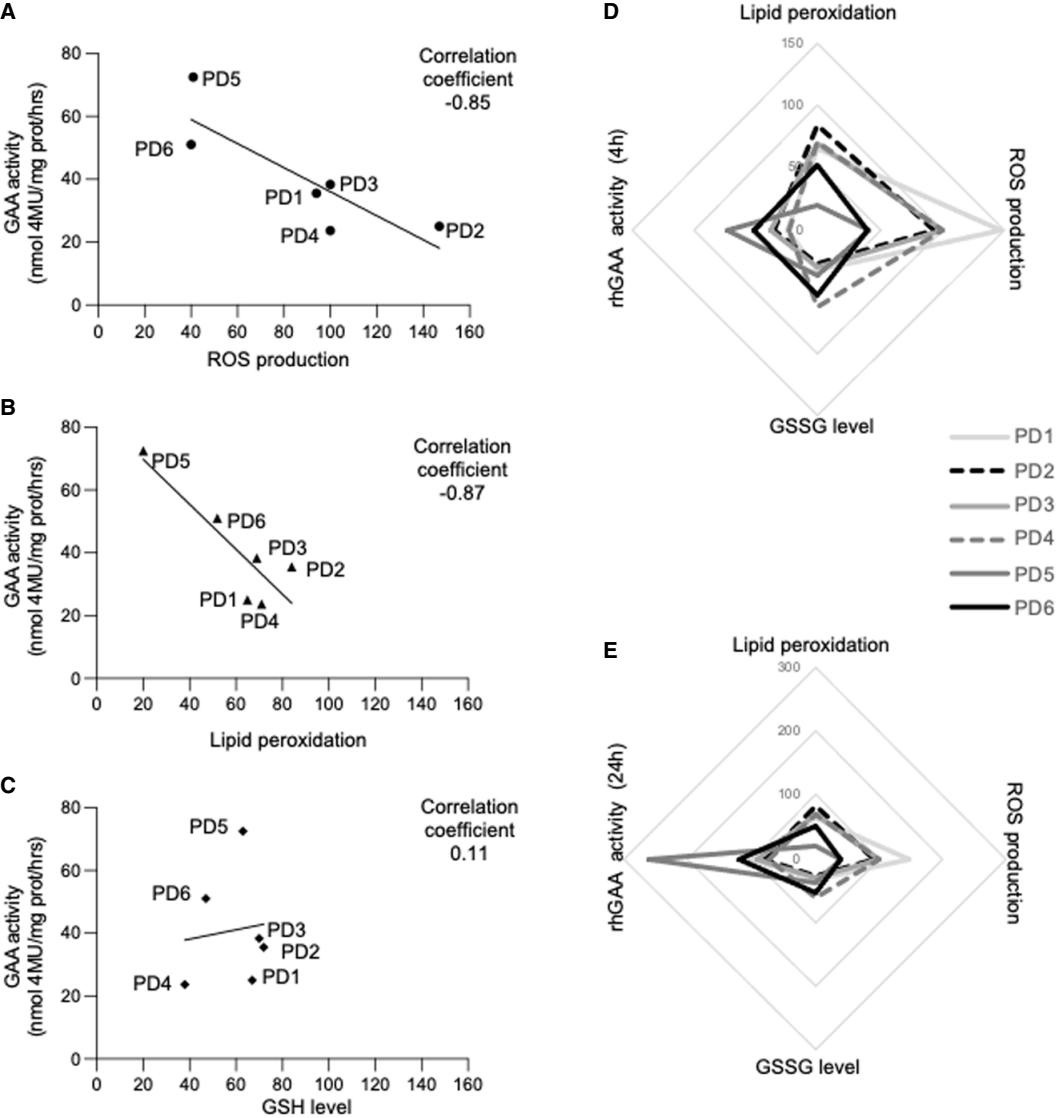
Stress inversely correlates with correction of GAA activity by rhGAA A–CCorrelations between the levels of single stress indicators and correction of GAA activity by rhGAA in 6 different PD fibroblasts. ROS production and lipid peroxidation inversely correlate with the GAA levels attained in cells after incubation with rhGAA for 4 h. The analysis of correlation was calculated, and the coefficient of Pearson is indicated.D, ESpider web charts show the trend of the four variables in each of the 6 PD cell lines analyzed after 4 (D) and 24 h (E) of incubation with rhGAA and support the inverse relationships between the stress levels and correction of GAA activity. Multiple correlation coefficient was calculated; the increase in oxidized glutathione (GSSG level) that is complementary to the reduction of reduced glutathione (GSH) is indicated. Data information: The scales used in D and E have been adjusted to the GAA activity at different time points. Source data are available online for this figure.

We also calculated a multiple correlation coefficient assessing the correlations between each of the three tests and rhGAA uptake after either 4‐ or 24‐h incubation with the recombinant enzyme. We fit a multiple linear regression model, and then, we calculated the coefficient of correlation between the predicted and observed values of the dependent variable. The multiple correlation coefficient was significant both at 4‐ (0.99) and at 24‐h incubation (0.97). Spider web charts show the trend of the 4 variables in each of the 6 PD cell lines studied after 4 h (Fig 3D) and 24 h (Fig 3E) and further support the inverse relationships between stress levels and GAA activity attained in rhGAA‐treated cells.

To further validate the hypothesis that oxidative stress impacts on correction of GAA activity by rhGAA, we induced additional stress in PD cells by sodium arsenite (ARS) and tert‐butyl‐peroxide (TBP) treatments. We evaluated the effects of different concentrations of both compounds on cell viability and cytotoxicity using a validated test (MTT assay, 3‐(4,5‐dimethylthiazol‐2‐yl)‐2,5‐diphenyltetrazolium bromide) (Arciello *et al*, 2011) (Fig 4A). Different concentrations of ARS (10, 30, 100 µM) did not affect cell viability for up to 4 h; after 6 h at the highest concentration (100 µM), cell viability was somehow reduced but still acceptable and largely above 50%. TBP did not impact significantly on cell viability at the lowest concentration (10 µM) for up to 6 h, while higher concentrations appeared more toxic for cells.

**Figure 4:**
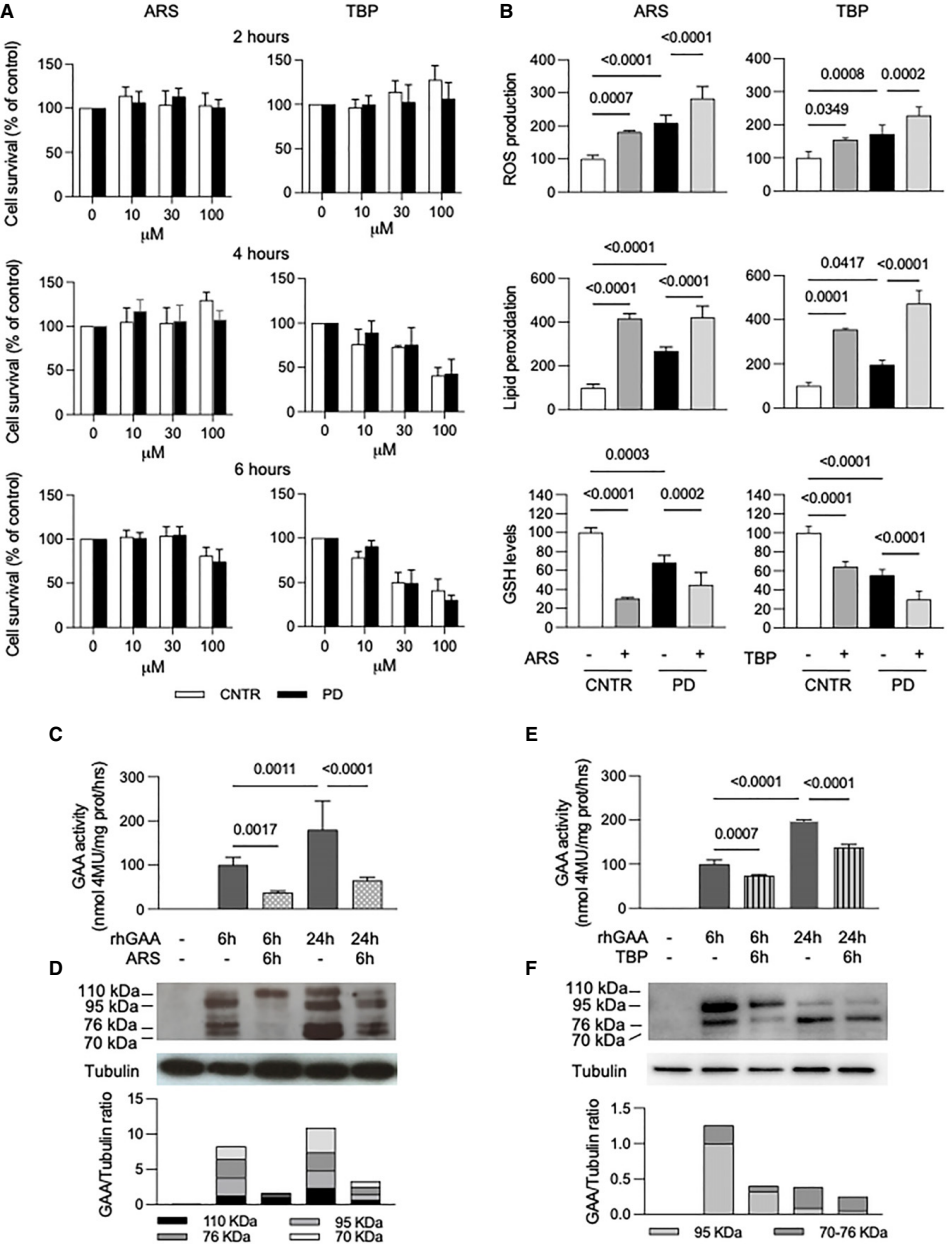
Induction of oxidative stress affect rhGAA uptake/processing ACell viability in control and PD fibroblasts (three different cell lines) measured by MTT assay in the presence of increased concentrations (0–100 µM) of either sodium arsenite (ARS) or tert‐butyl‐peroxide (TBP) at different time points. Data presented as a mean ± SD.BROS production, lipid peroxidation, and GSH levels in fibroblasts (3 different cell lines) after 6 h of treatment with 100 µM ARS and or 10 µM TBP. Both oxidative agents induced increase in oxidative stress in control (CNTR) and in PD cells. Data presented as a mean ± SD. Significance was calculated by one‐way ANOVA followed by Sidak's multiple comparisons test.C–F(C, E) GAA activity in PD fibroblasts (3 different cell lines) treated with rhGAA (gray bars), with rhGAA+ARS (dotted bars) and rhGAA+TBP (stripped bars). Data presented as a mean ± SD. Significance was calculated by one‐way ANOVA followed by Tukey's multiple comparison test. (D, F) Western blot analysis of GAA isoforms and quantitative analysis of the different enzyme isoforms (in a representative patient). The results indicate that ARS and TBP treatment reduces the amount of rhGAA internalized by cells and its processing into the mature forms (most evident at 6 h). Data information: In B, C, E, statistically significant *P*‐values are indicated. Figure 4D, tubulin: brightness +40%. Source data are available online for this figure.

As expected, both in controls and in patients’ cells incubated with 100 µM ARS and with 10 µM TBP for 6 h ROS levels and lipid peroxidation significantly increased, compared to untreated cells, with a concomitant decrease of GSH (Fig 4B). Western blot analysis of the autophagy marker LC3 in PD fibroblasts showed an increase of LC3‐II after ARS treatment (Fig EV2D), possibly indicating further impairment of autophagy, a result consistent with previous studies (Zhu *et al*, 2014). TBP treatment did not result in substantial changes.

We also assessed the correction of GAA activity by rhGAA as a function of time and of ARS concentration (Appendix Fig S3). Cells were incubated for 0, 2, 4, and 6 h with rhGAA, in the absence or in the presence of different ARS concentrations (10, 30, 100 µM). At each time point, cells were harvested and GAA activity was assayed. When present, ARS treatment was started 30 min before incubation with rhGAA. A dose‐dependent reduction of GAA activity in the presence of ARS was already detectable after 2 h and became increasingly evident at 4 and 6 h.

To study the effect of stress induction on rhGAA processing, PD fibroblasts were treated with 100 µM ARS and 10 µM TBP for 6 h and with rhGAA for 24 h (Fig 4D). ARS (Fig 4C and D) and TBP (Fig 4E and F) treatments reduced GAA activity both at 6 and at 24 h, and impacted not only on the amounts of the recombinant enzyme internalized by cells, but also on enzyme maturation into the active 70–76 kDa isoforms (most evident at 6 h).

### Modulation of stress impacts on correction of GAA activity by rhGAA

An easy and straightforward way to reduce stress in cultured cells is based on the use of antioxidants. Many of these molecules are known, are used both *in vitro* and *in vivo*, and reduce stress through different mechanisms. One of these compounds has been reported to activate autophagy through a calcium‐dependent mechanism (Li *et al*, 2021).

We tested idebenone (IDE), a short‐chain coenzyme Q analog that enhances superoxide formation (Buyse *et al*, 2011); edaravone (EDA), a free radical scavenger and antioxidant that reduces post‐ischemic brain injury and inhibits iron‐dependent peroxidation and mitochondrial permeability transition pore (Watanabe *et al*, 2008); N‐acetylcysteine (NAC), a membrane penetrating antioxidant that has anti‐inflammatory properties through regulation of NF‐κB and HIF‐1α activation and modulation of ROS (Samuni *et al*, 2013); resveratrol (RESV), an intracellular antioxidant that activates sirtuin 1 (SIRT1), is involved in mitochondrial biogenesis, and enhances peroxisome proliferator‐γ‐activated receptor coactivator‐1α (PGC‐1α) and FOXO (forkhead box) activities (Kulkarni & Cantó, 2015). These drugs have already been tested in different diseases and have been shown to have beneficial effects on motor function, cardiac hypertrophy, or neuroprotection (Rustin *et al*, 2002; Dolinsky *et al*, 2012; Kikuchi *et al*, 2013; Jaber & Polster, 2015; Reyes *et al*, 2017; Tardiolo *et al*, 2018; Yoshino, 2019). IDE and EDA are currently under clinical evaluation as adjunctive therapies for Duchenne muscular dystrophy and amyotrophic lateral sclerosis, respectively (ClinTrial.gov numbers: NCT03603288 and NCT04259255). With the exception of NAC (Fig EV3A), that in a previous study showed the ability to stabilize rhGAA (Porto *et al*, 2012), and EDA (Fig EV3B) that induced a maximum thermal shift of 5.94°C, the other antioxidants did not show direct chaperone properties in cell‐free system by differential scanning fluorimetry (DSF) (Fig EV3A).

**Figure EV3:**
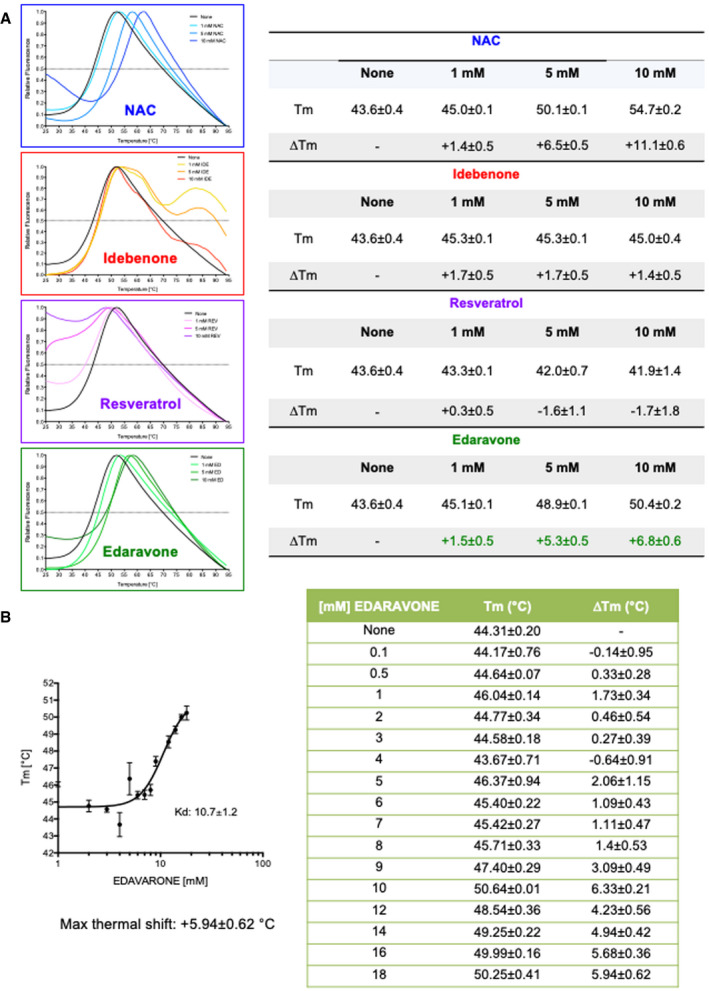
Effects of antioxidants on GAA heat stability by DSF Thermal scans of rhGAA in the presence of the pharmacological chaperone NAC and antioxidants. The table shows the melting temperatures and their relative shifts in the absence and in the presence of antioxidants.Determination of rhGAA‐edaravone interaction by differential scanning fluorimetry. The table shows the melting temperatures and their relative shifts in absence and in the presence of increasing concentrations of edaravone. Data presented as mean ± SD of data obtained in three different PD fibroblast cell lines. In each cell line, the analysis was performed in triplicate. Source data are available online for this figure.

As expected, all antioxidants significantly reduced the stress indicators used in previous experiments, with variable efficacy (Fig 5A).

**Figure 5:**
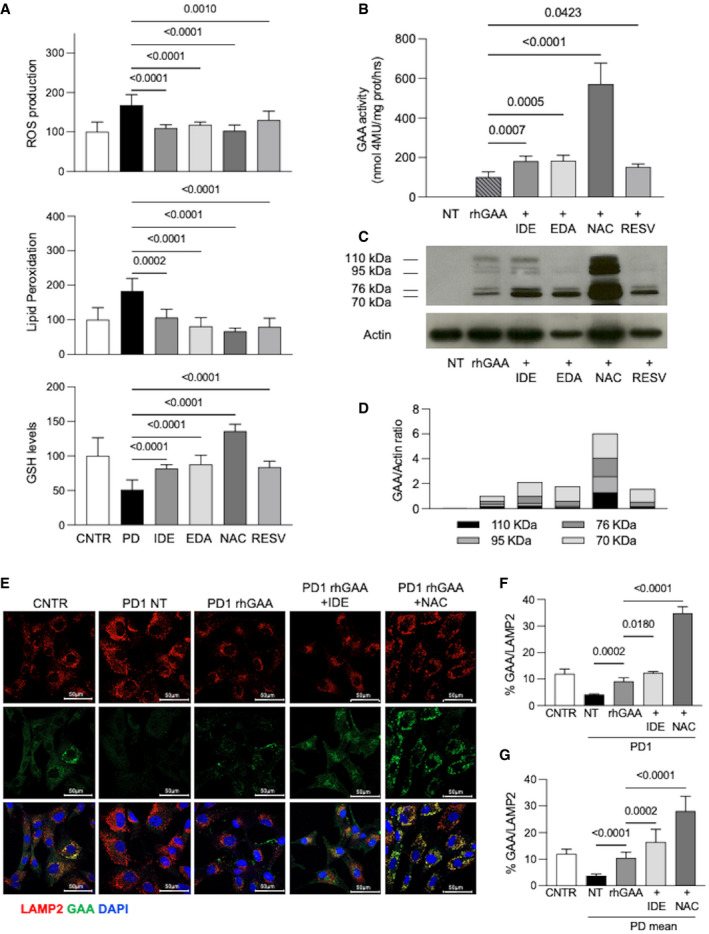
Effect of antioxidants on stress and ERT AEffect of antioxidants on ROS production, lipid peroxidation, and GSH levels in PD fibroblasts (three different cell lines for each treatment, each cell line assayed at least in duplicate). Cells were incubated for 24 h with idebenone (IDE) 0.5 µM; edaravone (EDA) 50 µM; N‐acetylcysteine (NAC) 0.5 mM; and resveratrol (RESV) 30 µM. Mean of control (CNTR) values was taken as equal to 100. The results are shown as mean ± SD.BRelative GAA activity increase in PD fibroblasts (three different cell lines, each cell line assayed in duplicate) treated with rhGAA alone and with rhGAA in combination with antioxidants. The effects of rhGAA alone are taken as 100. The results are expressed as means ± SD.C, DWestern blot analysis of GAA isoforms and quantitative analysis of the different enzyme isoforms. The image shown is representative of three independent experiments in different PD patients.EConfocal immunofluorescence analysis of GAA and LAMP2; representative fibroblast cell lines (PD1). Confocal 63× images; Scale bar 50 µm; Brightness +20%. Data obtained in 2 other cell lines (PD2, PD3) are reported in Fig EV4.FPercent of GAA/LAMP2 colocalization in PD1. The results are expressed as means ± SD of five images for each condition.GPercent of GAA/LAMP2 colocalization; mean of the analyses performed in 3 PD fibroblast cell lines. For each PD patient, five images for each condition were quantified. The results are expressed as means ± SD of five images for each condition. Data information: To calculate statistical significance, one‐way ANOVA was applied for all experiments followed by Dunnett’s test. Statistically significant *P*‐values are indicated. Source data are available online for this figure.

We then tested the effects of all these drugs on the correction of GAA activity by rhGAA in PD patient fibroblasts (*n* = 3). In all instances, co‐incubation for 24 h of rhGAA with antioxidants resulted into improved correction of GAA activity (with 0.52–4.72‐fold increases), compared to ERT alone (Fig 5B). In this experiment, the best‐performing drug was NAC that remarkably enhanced GAA activity, highly increased the amounts of GAA‐related polypeptides by Western blot analysis, and improved rhGAA processing into the active molecular isoforms of 76 and 70 kDa (Fig 5C and D). Though less efficiently, also the other drugs significantly improved GAA activity correction and GAA maturation (Fig 5C and D).

To investigate whether antioxidants enhance rhGAA lysosomal delivery, we performed an immunofluorescence analysis of rhGAA and LAMP2 colocalization in three PD fibroblasts. We selected two antioxidants (NAC, because of its apparent superiority in improving GAA activity and processing, and IDE that did not show a chaperone effect by DSF and is one of the drugs already in clinical evaluation to treat neuromuscular disorders). PD cell lines were incubated for 24 h with rhGAA alone or in combination with either NAC or IDE. GAA colocalization with the lysosomal marker LAMP2 was increased in cells treated with the co‐administration of rhGAA and antioxidants, compared to cells treated with rhGAA alone (Figs 5E and EV4). A quantitative analysis of rhGAA‐LAMP2 colocalization showed significant increases in each of the cell line studies (Figs 5F and EV4) and as a mean of the values obtained in all three cell lines (Fig 5G). Also in this case NAC was the drug that induced the most evident improvements of rhGAA lysosomal trafficking.

**Figure EV4:**
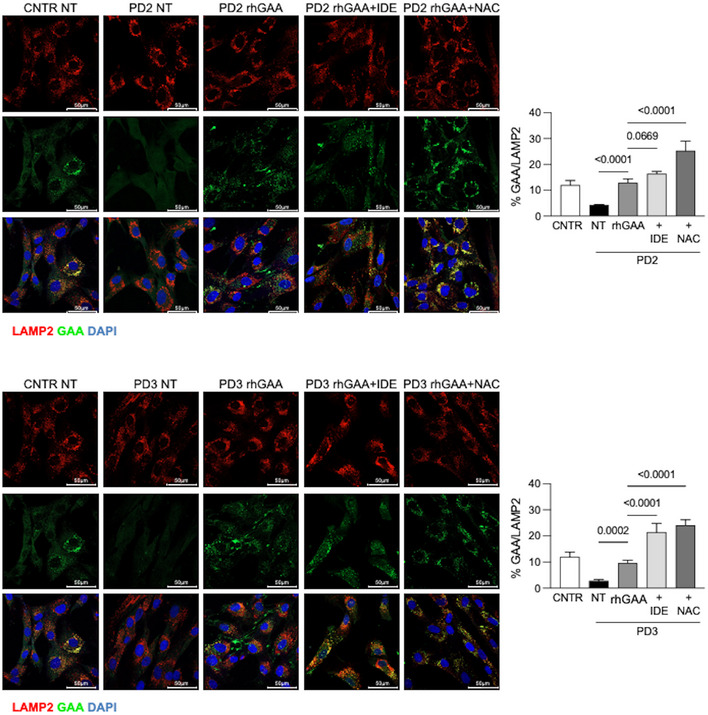
Immunofluorescence analysis of GAA‐Lamp2 in cells treated with rhGAA alone and in combination with antioxidants Confocal immunofluorescence analysis of GAA and LAMP2 in PD fibroblasts (PD2, PD3) and respective percent of GAA/LAMP2 colocalization. Fibroblasts were treated with rhGAA in the absence and in the presence of IDE or NAC. Control fibroblasts from Fig 5E and untreated PD cells are shown for comparison. Data information: Data presented as mean ± SD of five images for each condition in each patient. To calculate statistical significance, one‐way ANOVA was applied for all experiments followed by Dunnett’s test. Statistically significant *P* values are indicated. Confocal 63× images; scale bar 50 µm; brightness +20%. Source data are available online for this figure.

Western blot analysis showed that antioxidant treatments, with the exception of RESV, resulted in decreased LC3 total amounts (Fig EV2E) compared to untreated PD cells. This result is consistent with previous studies reported in the literature (Wang *et al*, 2014; Filomeni *et al*, 2015).

Physiological or pharmacological activation of autophagy also impacted on correction of GAA activity by rhGAA. Four‐hour starvation and activation of autophagy through mTOR inhibition by rapamycin or TRPML1 stimulation by ML‐SA1 and MK3‐83 resulted in significant increases of GAA activity and of GAA‐related peptides in PD fibroblasts treated with rhGAA (Fig EV5A and B).

**Figure EV5:**
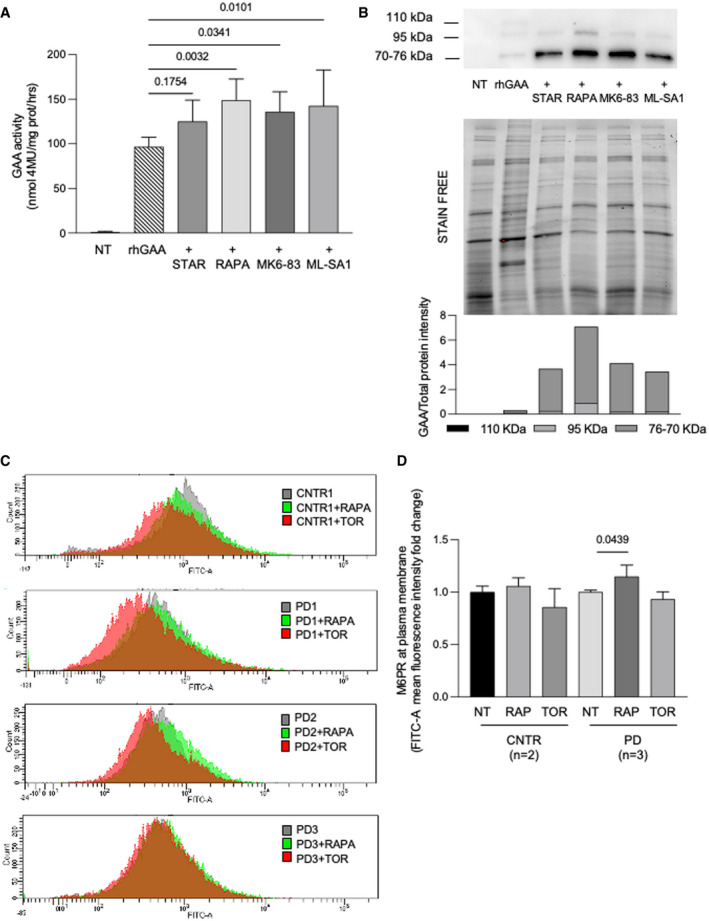
Effects of autophagy induction on correction of GAA activity by rhGAA and M6PR localization at the plasma membrane Relative GAA activity increase in PD fibroblasts (cell lines *n* = 3) treated with rhGAA alone and with rhGAA in combination with autophagy activators (starvation, STAR; rapamycin, RAPA; MK6‐83; ML‐SA1). The effects of rhGAA alone are taken as 100%. The results are expressed as means ± SD. ANOVA was applied followed by Dunnett’s multicomparison test.Western blot analysis of GAA isoforms and quantitative analysis of the different enzyme isoforms (top) normalized to stain free (middle). All drugs improved the amounts of rhGAA‐related polypeptides and the processing of rhGAA into the active isoforms (76–70 kDa). The image shown is representative of at least three independent experiments in different PD patients.FACS analysis of control and PD fibroblast shows the M6PR amount at plasma membrane before and after treatments with autophagy activators (rapamycin and torin1).Mean of results obtained in control (*n* = 2 cell lines) and PD fibroblast (*n* = 3 cell lines). For each cell line, the amount of M6PR‐positive cells was normalized, taking that observed in non‐treated fibroblasts as 1. Data presented as mean ± SD. ANOVA was applied followed by Sidak's multicomparison test. Source data are available online for this figure.

### Antioxidants increase the mannose‐6‐phosphate receptor (M6PR) at the plasma membrane of PD fibroblasts

The mechanisms underlying the effect of antioxidants on rhGAA are not clear. In principle, several factors may be implicated, for example, vesicle and membrane‐bound protein trafficking. We speculated that studying the availability of M6PR at the plasma membrane would be of interest in this respect. M6PR is a membrane‐bound protein that is crucial for the uptake and lysosomal trafficking of exogenous enzymes. We have already learned in previous studies that the M6PR intracellular distribution is impaired in PD cells compared to controls (Cardone *et al*, 2008). Published studies suggest that M6PR localization at the plasma membrane is affected in PD muscle cells and that some drugs, such as beta‐2 agonists, enhance ERT through improvements of M6PR expression at the plasma membrane (Koeberl *et al*, 2011). In keeping with previous work (Cardone *et al*, 2008), we found reduced amounts of intracellular M6PR in PD fibroblasts compared to controls, and reduced M6PR colocalization with the trans‐Golgi marker TGN46 (Appendix Fig S4).

We also assessed the amounts of M6PR localized at the plasma membrane of PD fibroblasts. We analyzed control and PD fibroblasts by fluorescence‐activated cell sorter (FACS) in ice‐cold medium to prevent the internalization of the M6PR and to label and detect only the fraction of M6PR exposed at the plasma membrane. A representative experiment of this analysis is shown in Fig 6A, while Fig 6B shows the average amount of M6PR exposed at the plasma membrane in PD fibroblasts in three independent experiments, indicating a significant reduction in PD cells compared to control cells (*P* = 0.0042).

**Figure 6:**
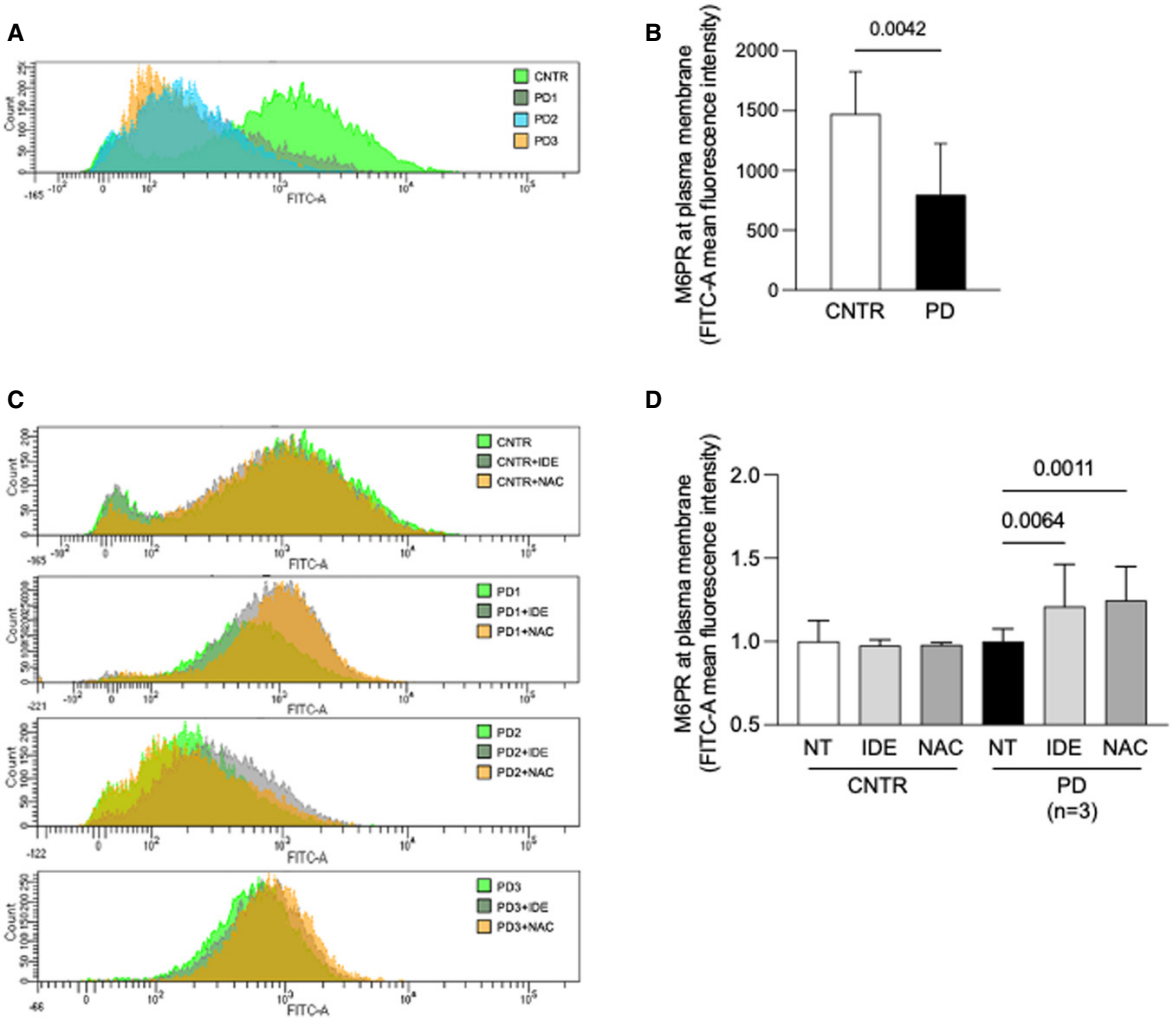
M6PR localization in PD cells and effect of antioxidants A, BFluorescence activated cell sorter (FACS) analysis of control and PD fibroblasts (*n* = 3). A representative experiment (A) and mean values of three independent experiments (B). Data presented as mean ± SD. A Student’s *t*‐test was applied for statistical analysis.CFACS analysis of control and PD fibroblast show the M6PR amount at plasma membrane before and after antioxidants treatments (NAC and IDE).DMean of results obtained in control (two cell lines in duplicate) and PD fibroblast (three different cell lines, each cell line tested in duplicate in three different experiments). For each cell line, the amount of M6PR‐positive cells was normalized, taking that observed in non‐treated fibroblasts as 1. Data presented as mean ± SD. Data information: For statistical analysis, a one‐way ANOVA followed by Sidak's post hoc test was applied. Source data are available online for this figure.

When three PD fibroblast cell lines were incubated with NAC and IDE, the amounts of M6PR localized at the plasma membrane significantly increased both with IDE (*P* = 0.0054) and with NAC (*P* = 0.0011) (Fig 6C and D).

We also tested the effects of autophagy enhancers on M6PR localization at the plasma membrane (Fig EV5C and D). Only rapamycin induced a significant increase in the amounts of the receptor available at the plasma membrane (*P* = 0.0439), although to a lower extent compared with antioxidants. This finding is in line with the enhancement of GAA activity correction in cells treated with rhGAA in combination with rapamycin (Fig EV5A and B). We may speculate that both correction of stress and stimulation of autophagy impact on correction of GAA activity. This is not surprising as stimulation of autophagy by itself reduces stress (Fig 2C) and since it has been shown that autophagy enhancement through TFEB overexpression results into accelerated vesicle trafficking in Pompe cells (Spampanato *et al*, 2013).

### Antioxidants enhance ERT *in vivo*

We then tested the effects of IDE and NAC on ERT *in vivo*. Six‐month‐old *Gaa* KO mice were treated with a single retro‐orbital injection of 40 mg/kg rhGAA alone, or in combination of either NAC (2 g/kg/die for 2 days) or IDE (100 mg/kg/die for 7 days) (Fig 7A and B). Although the recommended rhGAA dose in clinical use for PD patients is 20 mg/kg/injection, a dose 40 mg/kg is also accepted in human therapy and empirically used particularly in infantile‐onset PD patients (Poelman *et al*, 2020). From previous *in vivo* experiments done in our laboratory in the *Gaa* KO mouse model (Porto *et al*, 2009, 2012), the dose of 40 mg/k/injection is preferable in order to obtain detectable GAA activity increases in muscles (such as gastrocnemius and quadriceps) that are relatively refractory to ERT.

**Figure 7:**
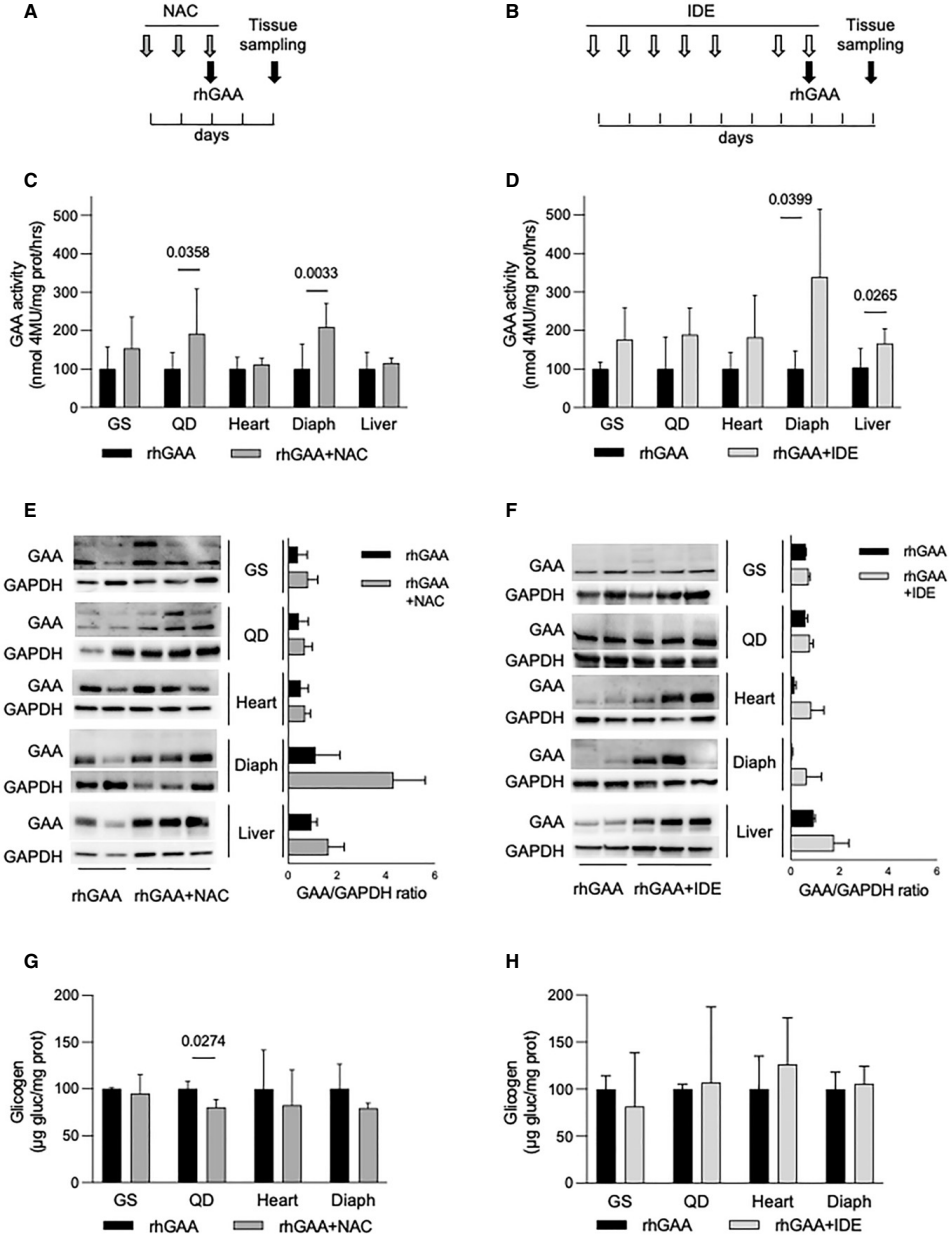
Effect of antioxidants in the PD mouse A, BCo‐dosing schedule of rhGAA and NAC (A) and rhGAA and IDE (B).CGAA activity in tissues from the Gaa KO mouse after treatment with rhGAA alone (black bars) (*n* of mice = 10) and with co‐dosing of rhGAA and 2 g/kg/day NAC (*n* of mice = 7, dark gray bars). Data presented as mean ± SD. Values obtained in tissues treated with rhGAA alone for each treatment are taken as 100. A Student’s *t*‐test was applied to compare the results in each of the tissues.DGAA activity in tissues from the Gaa KO mouse after treatment with rhGAA alone (black bars) (*n* of mice = 4) and with co‐dosing of rhGAA and 100 mg/kg/day IDE (*n* of mice = 6, light gray bars). Data presented as mean ± SD. Values obtained in tissues treated with rhGAA alone for each treatment are taken as 100. A Student’s *t*‐test was applied to compare the results in each of the tissues.E, FWestern blot analyses of GAA and quantitative analyses of the enzyme in representative tissues from the Gaa KO mouse after treatment with rhGAA alone (*n* of mice = 2) or in combination with NAC (*n* of mice = 3) (E) or IDE (*n* of mice = 3) (F). In F, data are presented as mean ± SD.GGlycogen assay in tissues from the Gaa KO mouse after treatment with rhGAA alone (*n* of mice = 3) or in combination with NAC (*n* of mice = 3).HGlycogen assay in tissues from the Gaa KO mouse after treatment with rhGAA alone (*n* of mice = 4) or in combination with IDE (*n* of mice = 6). Data information: In G and H, data are presented as mean ± SD. Values obtained in tissues treated with rhGAA alone for each treatment are taken as 100. A Student’s *t*‐test was applied to compare the results in each tissue. Source data are available online for this figure.

The dose of NAC was selected on the basis of pilot unpublished experiments aimed at optimizing the dosing and timing of co‐administration of ERT with this drug. The dose of IDE was selected on the basis of previous literature data (Yan *et al*, 2018).

Co‐administration of both NAC (Fig 7C) and IDE (Fig 7D) enhanced GAA activity correction in *Gaa* KO tissues, compared to the results obtained with ERT alone. In all tissues, there was a trend toward improvement with the co‐dosing protocols. Significant increases were obtained with NAC in quadriceps (*P* = 0.0358) and diaphragm (*P* = 0.0033), and with IDE in diaphragm (*P* = 0.0399) and liver (*P* = 0.0265). We also looked at the amounts of mature GAA in mice tissues by Western blot analysis (Fig 7E and F). In general, we found improved amounts of mature GAA in most of the animals treated with the combination of ERT and antioxidants, compared to those treated ERT alone. These data appear consistent with the results of GAA activity assay, although a quantitative analysis of Western blots failed to show statistical significance.

In addition, we measured the effect of co‐administration of rhGAA on tissue glycogen, in comparison with rhGAA alone. With the co‐administration of NAC, we observed a general trend toward decrease in tissue glycogen in animals treated with NAC and rhGAA, compared to those treated with rhGAA alone, that reached statistical significance in quadriceps (*P* = 0.0274) (Fig 7G). We did not observe significant changes in glycogen levels with the co‐administration of IDE (Fig 7H). However, these data are not surprising as it is not expected that a single injection of rhGAA is sufficient to induce major and detectable changes in glycogen storage.

## Discussion

The current standard of care for the treatment of PD is ERT with rhGAA. Other approaches are in phase of preclinical or clinical development. These include new‐generation recombinant enzymes with improved muscle‐targeting properties (neo‐rhGAA, AT‐GAA) (Zhu *et al*, 2009; Byrne *et al*, 2017; Xu *et al*, 2019), enhancement or stabilization of rhGAA by co‐dosing with a pharmacological chaperone (Porto *et al*, 2009; Parenti *et al*, 2014; Kishnani *et al*, 2017) or with beta‐2 agonist drugs (Koeberl *et al*, 2020), antisense oligonucleotide technologies (Goina *et al*, 2017; van der Wal *et al*, 2017), and gene therapy‐based strategies exploiting different viral vectors and gene expression cassettes (van Til *et al*, 2010; Wagemaker, 2014; Corti *et al*, 2017; Puzzo *et al*, 2017; Byrne *et al*, 2019; Doyle *et al*, 2019; Cagin *et al*, 2020). An approach based on reducing the synthesis of substrate (substrate reduction therapy) was also proposed (Douillard‐Guilloux *et al*, 2008).

These approaches are essentially based on replacing the defective mutant enzyme with a wild‐type functional enzyme and restoring the equilibrium between substrate synthesis and enzyme activity. The results of our studies propose a shift in this perspective and point to secondary cellular abnormalities as key therapeutic targets. We show that factors related to recipient tissues, in addition to the intrinsic properties of the recombinant enzyme, influence the response to ERT and possibly the outcome of patients.

We have shown that PD is associated with oxidative stress as a result of autophagy impairment, and that increased oxidative stress is present both in tissues from the mouse model of the disease and in cultured cells from patients. In the *Gaa* KO mouse, stress was most significantly increased in muscles. These findings are consistent with the fact that the impairment of autophagy and the accumulation of autophagic material are typically found and are more prominent in muscles (Lim *et al*, 2014) and indicate a link between oxidative stress and dysfunctional autophagy. The effects of drugs that enhance or block autophagy on oxidative stress further support this link.

The presence of mitochondrial abnormalities, a known consequence of ineffective autophagy, is consistent with previous studies (Lim *et al*, 2015) and with the notion that mitochondrial dysfunction is not only associated with primary defects of mitochondrial genes but is also impaired in a variety of non‐mitochondrial human diseases.

We also provided evidence, indicating that oxidative stress is detrimental for the ability of rhGAA to correct GAA activity in cells. PD‐cultured fibroblasts showed different levels of stress using standard tests that measure lipid peroxidation, ROS, and GSH levels. We found that the higher is the stress measured in cells, the lower is the GAA specific activity attained in cells after incubation with rhGAA, with a significant inverse correlation. Further induction of stress resulted into additional and remarkable decrease of GAA activity in rhGAA‐treated cells. This information is clinically relevant and may add another piece of information on the mechanisms underlying the variable responses of patients to ERT. Indeed, the experience of more than a decade of ERT in PD indicates that in some patients this therapy fails to restore normal histology in target tissues (Thurberg *et al*, 2006), that some tissues appear to be more refractory to treatment than others (Raben *et al*, 2002), that the stage of disease progression in tissues impacts on the response to treatment (Shea & Raben, 2009), and that the autophagic build‐up observed in affected muscles hampers efficient delivery of the therapeutic enzyme to lysosomes (Fukuda *et al*, 2006).

Correction of stress using common antioxidants resulted into improved correction of GAA activity in rhGAA‐treated cells and in enhanced processing and maturation of the enzyme. Two selected antioxidants, NAC and IDE, were also able to rescue GAA delivery to the lysosomes. Among the different antioxidants that we tested NAC showed the best results. This substantial effect of NAC is not surprising as this drug combines antioxidant properties with a direct, allosteric chaperone effect on GAA (Porto *et al*, 2012). The interactions of NAC with two non‐catalytic domains of the enzyme have been experimentally documented with co‐crystallization studies, together with the ability of NAC to prevent oxidation of a cysteine at position 938 (Roig‐Zamboni *et al*, 2017). This direct effect on the GAA protein provides an explanation for the apparent discrepancy between NAC superiority in terms of GAA activity enhancement, and the equivalent efficacy of NAC and IDE in improving M6PR localization at the plasma membrane of cells.

We also showed that autophagy enhancement resulted into improved correction of GAA activity by rhGAA. This suggests that multiple approaches directed toward correction of secondary abnormalities in cells may synergize with ERT in PD.

Why correction oxidative stress is beneficial for ERT needs to be clarified. We speculated that one of the mechanisms could be associated with an effect of stress on trafficking of vesicle, membranes, and membrane‐associated proteins. This assumption is supported by published evidence demonstrating that oxidative stress impacts on membrane composition and function (Khatchadourian *et al*, 2009; Li *et al*, 2012; Dielschneider *et al*, 2017) and that in other lysosomal storage diseases membrane composition impacts on SNAREs and the machinery involved in vesicle fusion (Fraldi *et al*, 2010). In principle, impaired vesicle trafficking may affect the recycling of M6PR, a membrane‐bound protein that is crucial for the sorting of lysosomal enzyme to lysosomes and is involved in the uptake and lysosomal delivery of most recombinant enzyme preparations used for ERT, including rhGAA. We know from previous work that M6PR localization is affected in fibroblasts from PD patients (Cardone *et al*, 2008). Indeed, we found that the amounts of M6PR at the plasma membrane of PD fibroblasts are reduced, and that this partial deficiency of M6PR available for rhGAA uptake and lysosomal trafficking is significantly improved by NAC and IDE. It is plausible, however, that the availability of M6PR at the plasma membrane is only one of several mechanisms implicated in the enhancing effect of antioxidants on ERT.

Antioxidants were effective in improving the level of enzyme correction also *in vivo*, in tissues that are commonly considered as important targets of treatment in a neuromuscular disorder like PD. With the co‐dosing of ERT and NAC, we reached significant improvements of GAA activity in quadriceps and diaphragm. Co‐administration of rhGAA and IDE improved GAA activity levels in diaphragm and liver. The improvements obtained in diaphragm may be relevant for patients’ outcome. Diaphragm dynamics have been shown to be severely affected in PD patients and to have a major impact on patient health conditions (van der Beek *et al*, 2011). Diaphragm has been selected as a site of injection in the first AAV‐based gene therapy study in PD (Corti *et al*, 2017).

In conclusion, our work provides proof‐of‐concept evidence that reducing oxidative stress, one of the secondary abnormalities observed in PD, is a strategy that may be pursued to enhance the effects of ERT. As antioxidants drugs are approved for human therapy and show in general good safety profiles, their use would represent a convenient and safe option as adjunctive treatments for PD.

However, before clinical translation of our results, several aspects remain to be clarified. The combination of antioxidants with ERT should be evaluated in long‐term preclinical *in vivo* studies to study the effects on animal performance and behavior and should be compared with the effect of antioxidants alone.

In the *in vivo* evaluation of the synergy between ERT and antioxidants the bioavailability and pharmacokinetics of drugs should be carefully considered. For example, in the *in vivo* experiments NAC, that was the best‐performing drug *in vitro*, did not appear substantially superior to IDE. This may reflect the influence of factors such as intestinal absorption, barriers, bioavailability, pharmacokinetics, and drug metabolism in liver and other organs.

The mechanisms by which antioxidants impact on ERT should be better clarified. We showed that NAC and IDE improve the amounts of M6PR available at the plasma membrane, but we are aware that there might be other factors that impact on rhGAA uptake and GAA activity correction.

It is possible to speculate that other, more potent drugs with antioxidant properties may show a better therapeutic profile. A screening for further molecules may be feasible with high‐content platforms, taking advantage of the indicators analyzed in our study.

In principle, the same approach may be extended to other therapeutic agents, such as new enzyme preparations, and possibly gene therapy, with the ultimate objective of improving the outcome and the quality of life of PD patients. The identification of secondary dysregulation of cellular pathways as therapeutic targets may also open the way to similar studies in other lysosomal storage disorders in which secondary abnormalities participate in the disease pathophysiology.

## Materials and Methods

### Patients’ cells and animal model

Human PD fibroblasts were available at the cell bank of the Department of Translational Medical Sciences (DISMET), Section of Pediatrics, Federico II University, Naples.

Fibroblasts were grown in Dulbecco’s modified Eagle’s medium (Invitrogen, NY, USA) and supplemented with 20% fetal bovine serum (Invitrogen, NY, USA), 2 mM l‐glutamine, and antibiotics (Invitrogen, NY, USA). Human primary myoblasts were provided by the "Telethon BioBank" (Telethon Research Service, Istituto Nazionale Neurologico "Carlo Besta", Milano, Italy). Myoblasts were expanded with proliferation medium: 60% high‐glucose Dulbecco’s modified Eagle’s medium (Invitrogen, NY, USA), 20% fetal bovine serum (Invitrogen, NY, USA), 10 ng/ml EGF (R&D Systems, Minneapolis, MN, USA), 2 ng/ml β‐FGF (R&D Systems, Minneapolis, MN, USA), 10 μg/ml insulin (Roche Applied Science, Indianapolis, IN, USA), and 1% Penicillin‐Streptomycin‐Glutamine mix solution (Invitrogen, NY, USA). Cells were grown in a 5% CO_2_ humidified atmosphere at 37°C.

The experimental procedures involving cells derived from human subjects were conducted in accordance with the principles of the World Medical Association (WMA) Declaration of Helsinki and with the Department of Health and the Human Services Belmont Report.

Fibroblast and myoblasts cell lines were obtained for diagnostic purposes. Patients or their legal guardians provided their informed consent to the storage of cells and to their use for future research purposes.

A KO PD mouse model obtained by insertion of *neo* into the GAA gene exon 6 (Raben *et al*, 1998) was purchased from Charles River Laboratories (Wilmington, MA) and was maintained at the Cardarelli Hospital's Animal Facility (Naples, Italy) and at the TIGEM Animal Facility (Pozzuoli, Italy). Animal studies were performed according to the EU Directive 86/609, regarding the protection of animals used for experimental purposes (IACUC project no 523/2015‐PR approved by the Italian Ministry of Health). Every procedure on mice was performed with the aim of ensuring that discomfort, distress, pain, and injury would be minimal. Mice were euthanized following anesthesia.

### GAA activity assay

Fibroblasts were harvested by trypsinization, resuspended in water, and disrupted by three cycles of freezing and thawing. Tissues were immersed in water, disrupted by tissue lyser (50 oscillations per minute for 2 min) (Qiagen, Hilden, Germany) and three cycles of freezing and thawing. Protein concentration was measured according to the method of Lowry (Lowry *et al*, 1951).

GAA activity was assayed by using the fluorogenic substrate 4‐methylumbelliferyl‐alpha‐D glucopyranoside (Sigma‐Aldrich, St. Louis, MO, USA). Cell homogenates (10 μg protein for fibroblasts; 50 μg for tissues) were incubated at 37° for 60 min with 2 mM substrate in 0.2 M acetate buffer pH 4.0 in incubation mixtures of 20 μl (fibroblasts) or 50 μl (tissues). Reactions were stopped by adding 1 ml 0.5 M glycine carbonate buffer, pH 10.7, and fluorescence was read on a Turner Biosystems fluorometer Modulus 9200 (360 nm excitation, 450 nm emission).

### Western blot analysis

Cell extracts were obtained by vortexing cell suspensions for 30 min in ice in RIPA buffer with the addition of a protease inhibitors cocktail (Roche Diagnostics, Mannheim, Germany) and of phosphatase inhibitors. Tissue extracts were obtained by lysing samples in a tissue lyser (50 oscillations per minute for 2 min) (Qiagen, Hilden, Germany) in RIPA buffer with the addition of a protease inhibitors cocktail (Roche Diagnostics, Mannheim, Germany) and phosphatase inhibitors, and by five cycles of freezing and thawing. Samples were centrifuged at 14,000 *g* for 30 min at 4°C. Protein concentration was measured according to the method of Lowry (Lowry *et al*, 1951).

Twenty μg of proteins for cells or 30–50 μg for tissues was subjected to sodium dodecyl sulfate‐polyacrylamide gel electrophoresis (4–15% acrylamide in different experiments), and proteins were transferred onto nitrocellulose membranes (Amersham, Freiburg, Germany). After blocking with 5% milk, membranes were washed with Tris buffer saline containing 0.1% Tween‐20 (TBS‐T), incubated overnight at 4°C with primary antibodies (Table 2), and with HRP‐coupled secondary antibodies (Table 2) for 1 h at room temperature. Immunoreactive proteins were detected by chemiluminescence (ECL, Amersham, Freiburg, Germany). Images were captured by a CAWOMAT 2000 IR processor or by ChemiDoc™ XRS^+^ with Image Lab™ Software. ImageJ (NIH) or ImageLab software (Bio‐Rad Laboratories, Hercules, CA, USA) was used for band densitometric quantification. These software tools allow for correct quantitative analysis also in Western blots showing band distortion. For quantification, bands were normalized to endogenous reference proteins or to stain free gels. For stain‐free membranes, the UV light obtained from the entire lane was measured (representing the total protein content in the lane).

**Table 2:** Antibodies used and working concentrations.

Antibody	Producer	Dilution	Working concentration
Anti‐LC3	Novus Biologicals, Littleton, CO, USA	1:500	2 μg/ml
Anti‐phospho ERK	Cell Signaling, Danvers, MA, USA	1:1,000	0.2 μg/ml
Anti‐p62	Sigma‐Aldrich, St. Louis, MO, USA	1:1,000	1.1 μg/ml
Anti‐GAA	PRIMM	1:500	Not available
Anti‐GAPDH	Ambion, Austin, TX, USA	1:2,000	2 μg/ml
Anti‐actin	Sigma‐Aldrich, St. Louis, MO, USA	1:2,000	1.1 μg/ml
Anti‐tubulin	Sigma‐Aldrich, St. Louis, MO, USA	1:2,000	2.7 μg/ml
Total OXPHOS Rodent WB	Abcam, Cambridge, UK	1:250	6 μg/ml
Anti‐Mfn2	Abcam, Cambridge, UK	1:1,000	1 μg/ml
Anti‐rabbit IgG HRP‐conjugated	Bio‐Rad Laboratories, Hercules, CA, USA	1:2,000	0.5 μg/ml
Anti‐mouse IgG HRP‐conjugated	Bio‐Rad Laboratories, Hercules, CA, USA	1:2,000	0.5 μg/ml
Anti‐MTCO1	Abcam, Cambridge, UK	1:1,000	1 μg/ml
Anti‐IGF‐II R	Novus Biologicals, Littleton, CO, USA	1:1,000	1 μg/ml
Anti‐TGN46	Bio‐Rad Laboratories, Hercules, CA, USA	1:750	0.33 μg/ml
Anti‐rabbit Alexa Fluor 488 or 596	Invitrogen Corporation, Carlsbad, CA, USA	1:1,000	2 μg/ml
Anti‐mouse Alexa Fluor 488 or 596	Invitrogen Corporation, Carlsbad, CA, USA	1:1,000	2 μg/ml

### Oxidative stress assays

ROS levels were evaluated by 2′,7′‐dichloro‐dihydrofluorescein (DCFDA), according to published procedures (Del Giudice *et al*, 2017). Cells were washed twice with warm phosphate‐buffered saline supplemented with 1 mM CaCl_2_, 0.5 mM MgCl_2_ and 30 mM glucose (PBS plus), harvested by trypsinization, centrifuged at 1,000 *g* for 10 min, and resuspended in PBS plus at a density of 1 × 10^5^ cells/ml. Cells were then incubated with 25 μM H_2_‐DCFDA (Sigma‐Aldrich, St. Louis, MO, USA) for 30 min at 37°C and resuspended in a final volume of 200 μl. Emission spectra were acquired at 488 nm (excitation) and 525 nm (emission) using a PerkinElmer LS50 spectrofluorometer. ROS production was expressed as the percentage of DCF fluorescence intensity of the sample under test compared with untreated samples.

To measure ROS levels in mice tissues, samples were immersed in PBS plus (weight/volume ratio 1:10) and homogenized by tissue lyser (40 oscillations per minute for 3 min). Ten microlitre of each sample was incubated with 25 μM H_2_‐DCFDA for 30 min at 37°C and assayed as described above. Biological triplicates were analyzed for each cell line or tissue sample; each assay was performed in technical triplicate.

Lipid peroxidation was analyzed using the thiobarbituric acid‐reactive substances (TBARS) assay. Cells were harvested by trypsinization and centrifuged at 1,000 *g* for 10 min, and 5 × 10^5^ cells were resuspended in 1 ml of 0.67% thiobarbituric acid (TBA) (Sigma‐Aldrich, St. Louis, MO, USA). After the addition of 300 μl, 20% trichloroacetic acid (TCA) (Sigma‐Aldrich, St. Louis, MO, USA) samples were heated at 95°C for 30 min, incubated in ice for 10 min, and centrifuged at 3,000 *g* for 5 min at 4°C. Absorbance was read at 532 nm. Lipid peroxidation levels were expressed as percentage of absorbance at 532 nm of the sample under test, compared to untreated samples.

To measure lipid peroxidation in mouse tissues, samples were lysed as described for DCFDA and the assay was performed as described above.

Biological triplicates were analyzed for each cell line or tissue sample; each assay was performed at least in technical duplicate.

Intracellular glutathione levels and activity were assayed by 5,5′‐dithiobis‐2‐nitrobenzoic acid (DTNB). Cells were harvested by trypsinization, centrifuged at 1,000 *g* for 10 min, and resuspended in RIPA buffer, containing protease inhibitors. After incubation for 30 min in ice, lysates were centrifuged at 14,000 *g* for 30 min at 4°C. Supernatant protein concentration was determined by the Lowry assay (Lowry *et al*, 1951). Fifty μg of proteins was incubated with 3 mM ethylenediaminetetraacetic acid (EDTA) and 144 μM DTNB (Sigma‐Aldrich, St. Louis, MO, USA) in 30 mM Tris–HCl buffer, pH 8.2, and centrifuged at 14,000 *g* for 5 min at room temperature. Absorbance was measured at 412 nm using a multiplate reader (Epoch Biotek Winooski, USA). GSH levels were expressed as percentage of TNB absorbance of the sample under test compared with untreated samples.

Biological triplicates were analyzed for each cell line; each assay was performed in technical triplicate.

### MTT assay

Cells were seeded in 96‐well plates at a density of 1 × 10^4^/well. Twenty‐four hours after seeding, different concentrations of sodium arsenite (ARS) or tert‐butyl‐peroxide (TBP) (10–100 µM) were added to cells for different times (2, 4, and 6 h). After incubation, cell viability was measured by the tetrazolium salt colorimetric assay (MTT) (Arciello *et al*, 2011). Cell survival was expressed as percentage of viable cells in the presence of either ARS or TBP compared with untreated cells. Biological triplicates were analyzed for each cell line; each assay was performed in technical triplicate.

### Glycogen assay

Glycogen concentration was assayed in tissue lysates by measuring the release of glucose after digestion with *Aspergillus niger* amyloglucosidase, using a commercial kit (Sigma‐Aldrich, St Louis, MO, USA) according to manufacturer instructions. Data were expressed as μg of glycogen/mg of protein.

### Immunofluorescence analysis

To study the distribution of M6PR, human fibroblasts grown on coverslips were fixed using 4% PFA, permeabilized with 0.05% saponin, and blocked with 5% bovine serum albumin (BSA), in PBS with the addition of 50 mM NH_4_Cl, 0.02% NaN_3_ for 1 h. Cells were incubated overnight with an anti‐IGF‐II R primary antibody (Table 2); cells were then incubated with an anti‐mouse IgG secondary antibody (Table 2) in blocking solution and then mounted in Vectashield mounting medium with DAPI (Vector Laboratories, Burlingame, CA).

To study the distribution of LC3‐COX1, human fibroblasts grown on coverslips were fixed using methanol, permeabilized with 0.05% saponin, and blocked with 5% BSA, in PBS with the addition of 50 mM NH_4_Cl for 30 min. Cells were incubated with an anti‐LC3 rabbit polyclonal antiserum (Table 2), and with an anti‐MTCO1 mouse antiserum. Cells were then incubated with anti‐rabbit IgG and anti‐mouse IgG secondary antibodies in blocking solution (Table 2), then mounted in Vectashield mounting medium with DAPI.

Images were taken using a Zeiss LSM700 confocal microscope (Carl Zeiss, Jena, Germany) integrated with the AxioCam MR camera and 63× oil objective. Digital images were captured by using Zeiss AxioVision software, and maximum intensity projections were generated using ImageJ software.

### Electron microscopy

For electron microscopy (EM), muscle tissues, fibroblasts, and myoblasts were fixed in 1% glutaraldehyde in 0.2 M HEPES buffer and post‐fixed in OsO4 and uranyl acetate. After dehydration through a graded series of ethanol, the samples were embedded in the Epoxy resin (Epon 812, Sigma‐Aldrich) and polymerized at 60°C for 72 h. Thin 60‐nm sections were obtained using a Leica EM UC7 microtome. EM images were acquired from thin sections using a FEI Tecnai‐12 electron microscope equipped with a VELETTA CCD digital camera (FEI, Eindhoven, The Netherlands). To count the number of mitochondria within the cells, EM images were acquired at the same magnification. Normal and abnormal mitochondria were manually counted on EM images.

### Differential scanning fluorimetry

Thermal stability scans of rhGAA were performed as described in Porto *et al* (2012). Briefly, the enzyme was diluted 48‐fold (0.1 mg/ml in 25 mM Na‐phosphate buffer, pH 7.4, 150 mM NaCl, and 1:1,000 SYPRO Orange dye (Life Technologies)). Thermal scans were performed in triplicate in absence or in presence of 1 mM, 5 mM, and 10 mM antioxidants, with steps of 1°C per minute in the range 25–95°C in a Applied Biosystems Real‐Time PCR Instrument. Fluorescence was normalized to the maximum value within each scan to obtain relative fluorescence. Melting temperatures were calculated according to Niesen *et al* (2007). The standard deviations for each melting temperature were calculated from three replicates.

The dissociation constant (*K*_D_) of Edaravone was measured by thermal stability scans of rhGAA according to (Vivoli *et al*, 2014). DSF scans were performed as described above, in the range 0‐18 mM Edaravone. The melting temperature values were plotted as function of ligand concentration. Experimental data were best fitted according to a simple cooperative model equation reported in Vivoli *et al* (2014) by using the software GraphPad Prism (GraphPad Software, San Diego, California, USA).

### FACS analysis

To obtain a quantitative analysis of M6PR availability at the plasma membrane, PD and control fibroblasts were detached and suspended in blocking buffer (1% FBS in PBS). After incubating for 30 min on ice, the cells were washed and suspended in blocking buffer containing excess of a mouse anti‐IGF‐II R antibody (Table 2) for 30 min on ice. Cells were then washed, resuspended in blocking buffer containing an 488 Alexa Fluor anti‐mouse secondary antibody (Table 2) (Invitrogen Corporation, Carlsbad, CA, USA) for 30 min in ice, filtered through 70‐μm filcons (BD Biosciences Pharmingen, San Jose, CA, USA), and analyzed using a BD Biosciences FACS Aria III (BD Biosciences Pharmingen, San Jose, CA, USA).

### *In vivo* experiments

For *in vivo* experiments, we treated 6‐month‐old *Gaa* KO mice. NAC‐treated mice (*n* = 7) received 2 g/kg/day for 3 days by gavage (Fig 7A). On the third day, they also received a single retro‐orbital injection of 40 mg/kg rhGAA. IDE‐treated mice (*n* = 6) received intra‐peritoneal injections (100 g/kg/day) for 6 days (over a period of 7 days, with an interruption on Sunday, Fig 7B). On the last day of treatment with IDE, they received a single retro‐orbital injection of 40 mg/kg rhGAA. Animals were euthanized 48 h after rhGAA injection, and tissues were harvested for GAA assay, Western blot analysis and glycogen assay. The results were compared with those obtained in mice treated with rhGAA alone. In each experiment, GAA activity and glycogen content obtained in tissues from mice treated with rhGAA alone were taken as 100%.

### Statistical analyses

For *in vitro* studies, *n* indicates experimental replicates. For animal studies, *n* indicates the number of animals per treatment cohort. Bar graphs represent the mean, with error bars showing either the standard deviation of the mean for biological replicates or the standard deviation for technical replicates. Significance *P*‐values between means were computed using either the Student’s *t*‐test for comparison of two groups, the one‐way ANOVA followed by different post hoc tests (Tukey, Sidak, or Dunnett) for multiple treatment groups, the Pearson correlation test for association between two variables in GraphPad Prism version 9.1.1. Multiple correlation analyses were calculated by R environment (Stats package).

## Author contributions

AT performed and/or supervised experimental procedures and participated in the experimental design. CD performed and/or supervised experimental procedures. SS performed experimental procedures (immunofluorescence analysis of rhGAA trafficking). NM performed experimental procedures (rhGAA uptake studies). AI performed experimental procedures (characterization of mitochondrial function). EP performed electron microscopy studies. FZ participated in immunofluorescence analysis of M6PR subcellular localization. EN performed experimental procedures in mice. SF performed experimental procedures in cultured fibroblasts. CP performed experimental procedures (rhGAA uptake studies). MC performed experimental procedures (oxidative stress). RI performed rhGAA thermal stability and DSF studies in the presence of antioxidants. MM supervised rhGAA thermal stability and DSF studies in the presence of antioxidants. RP performed electron microscopy studies. DLM supervised experimental procedures. PI performed the cell viability tests. DMM participated in the experimental design of cell and tissue oxidative stress studies. MADM contributed to the experimental design. GP designed the experimental plan.

## Conflict of interest

G.P. received honoraria or travel reimbursement from TAKEDA, Sanofi‐Genzyme, and Orchard therapeutics. This work was supported in part by Spark Therapeutic Inc.

## For more information

- OMIM website of Pompe disease: https://omim.org/entry/232300
- Orphanet website for Rare Diseases: https://www.orpha.net/consor/cgi‐bin/Disease_Search_Simple.php?lng=EN&diseaseGroup=pompe+disease
- Pompe disease variation database Erasmus Medical Center: https://www.pompevariantdatabase.nl/pompe_mutations_list.php?orderby=aMut_ID1
- Associazione Italiana Glicogenosi onlus: https://www.aig‐aig.it/

## Supporting information



Appendix

Expanded View Figures PDF

Source Data for Expanded View

Source Data for Figure 1

Source Data for Figure 2

Source Data for Figure 3

Source Data for Figure 4

Source Data for Figure 5

Source Data for Figure 6

Source Data for Figure 7

## Data Availability

This study includes no data deposited in external repositories.
