# Supplementary material for: Correction of oxidative stress enhances enzyme replacement therapy in Pompe disease
Source: EMBO Mol Med. 2021 Oct 4;13(11):e14434. doi: 10.15252/emmm.202114434 (PMC8573602; doi:10.15252/emmm.202114434)
Supplement: Supplementary file 1 — Appendix [file EMMM-13-e14434-s010.pdf]

Appendix

Appendix Figure S1-Oxidative stress in single PD fibroblast cell lines

Appendix Figure S2-Time course of the effects of starvation on ROS levels in PD fibroblasts

Appendix Figure S3-Time course of ARS

Appendix Figure S4-M6PR localization in PD cells and effect of antioxidants

Appendix Figure S1

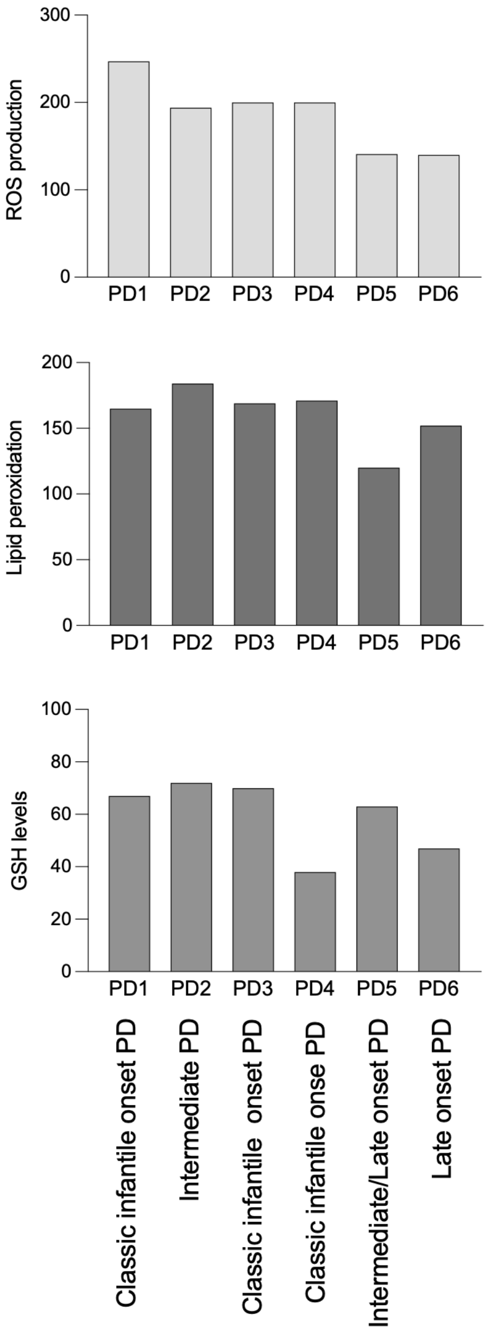

Oxidative stress in single PD fibroblast cell lines.

Oxidative stress biochemical markers (ROS production, lipid peroxidation, GSH levels) in individual PD patient fibroblasts. The PD clinical form for each patient is indicated.

Appendix Figure S2-Time course of the effects of starvation on ROS levels in PD fibroblasts

Appendix Figure S2

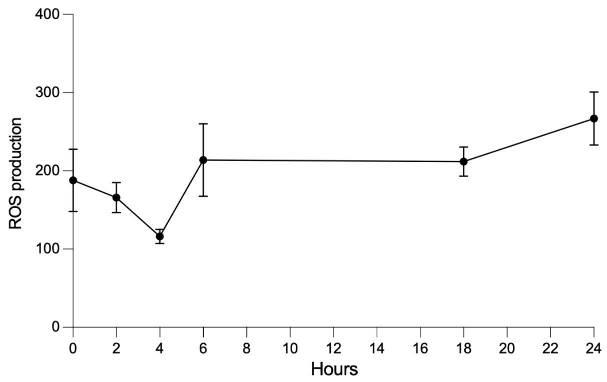

Time course of the effects of starvation on ROS levels in PD fibroblasts

Time course of the effect of starvation on ROS production in PD fibroblasts (n=3). Starvation induced an initial decrease in the first 4 hours (likely mediated by autophagy induction), followed by progressive increase of stress up to 24 hours.
Data information: Data presented as mean ± SD.

Appendix Figure S3

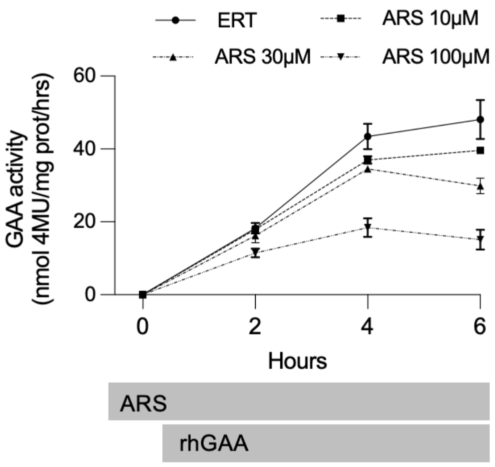

Time course of ARS

Time course of the effects of ARS on the correction of GAA activity by rhGAA in fibroblasts (n=3), indicating a deleterious effect of ARS on the correction of GAA acitivity in PD cells. Data presented as a mean ± SD of experiments performed on three PD patient cell lines.

Appendix Figure S4

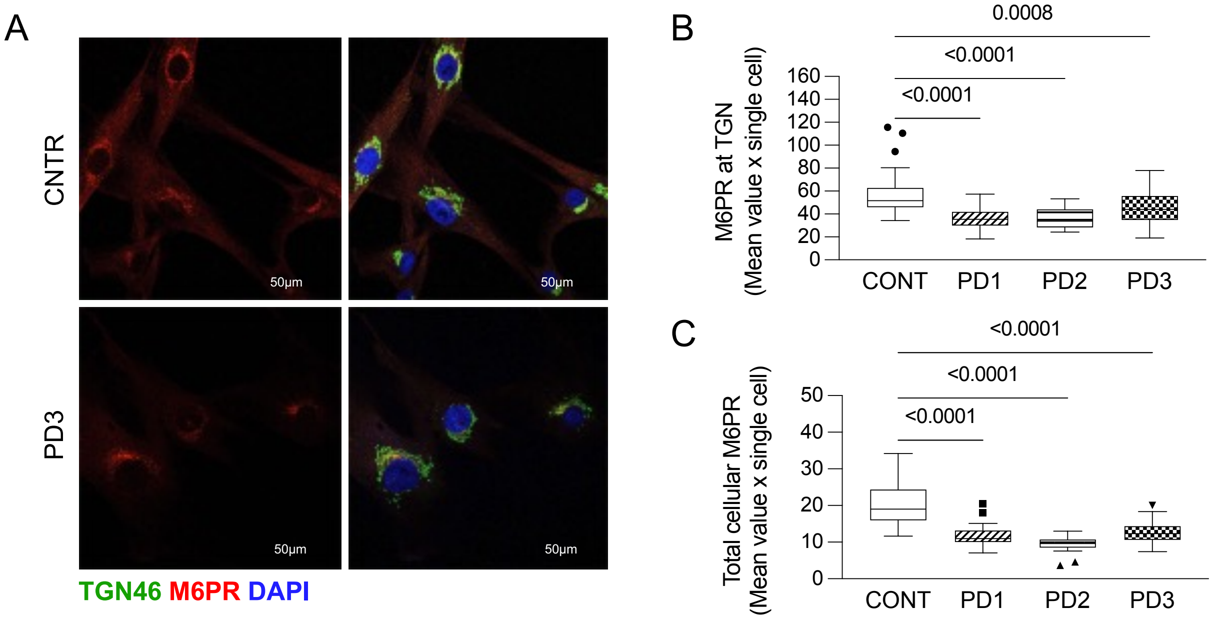

M6PR localization in PD cells and effect of antioxidants.

A. Immunofluorescence analysis of M6PR in PD fibroblasts and co-localization with the trans-Golgi marker TGN46. Confocal 63x images; Scale bar 50µm; Brightness +50%; contrast +20%.
B. C. Quantitative analysis of the M6PR amount localized at the trans-Golgi network (B) and total amount in cells (C). A significant reduction of the M6PR colocalization with TGN46 and of the total amount of M6PR signal was detectable in PD fibroblasts (n=3) compared to controls. Data presented as mean ± SD. ANOVA was applied followed by Dunnett's multicomparison test.
