# Supplementary material for: Correction of oxidative stress enhances enzyme replacement therapy in Pompe disease
Source: EMBO Mol Med. 2021 Oct 4;13(11):e14434. doi: 10.15252/emmm.202114434 (PMC8573602; doi:10.15252/emmm.202114434)
Supplement: Supplementary file 2 — Expanded View Figures PDF [file EMMM-13-e14434-s008.pdf]

Expanded View Figures

Figure EV1. Characterization of mitochondria in gastrocnemii from the PD mouse model and in cultured PD patient cells.

A Ultrastructural analysis of gastrocnemii from the *Gaa* KO mouse showed intra-lysosomal glycogen storage (white arrow), active mitophagy (arrowhead), abnormal mitochondria (black arrow).

B, C Quantitative analysis of the number of mitochondria (B) and of morphologically abnormal mitochondria (C) in 15 low-magnification (16×) electron microscopy fields showing significantly increased number of abnormal mitochondria ($P = 0.0038$) in *Gaa* KO compared to wild-type animals. Data presented as mean ± SD of at least 12 fields for each mouse muscle. Student's *t*-test was applied.

D Western blot analysis of the levels of OXPHOS complexes in mitochondrial preparations from the Gaa KO gastrocnemii, showing increased levels of the markers tested.

E, F Number of mitochondria (E) and mitochondrial length (F) in PD fibroblasts and myoblasts compared to, respectively, control cells. Data presented as mean ± SD of at least 15 fields for each cell line. A Student's *t*-test was applied.

G, H Co-staining of COX1 with LC3 (G) and quantitative analysis (H) showing significantly increased colocalization of these markers in PD cells compared to control cells under standard culture conditions. Data presented as mean ± SD of five images for each cell line. A Student's *t*-test was applied. Confocal 63× images; scale bar 50 μm; contrast +15%; brightness +25%.

Data information: In B, E, F, boxes include values between upper and lower quartiles, and central band corresponds to median, whiskers, and lower extremes to higher and lower values. Outlier values are indicated as dots.

Source data are available online for this figure.

© 2021 The Authors

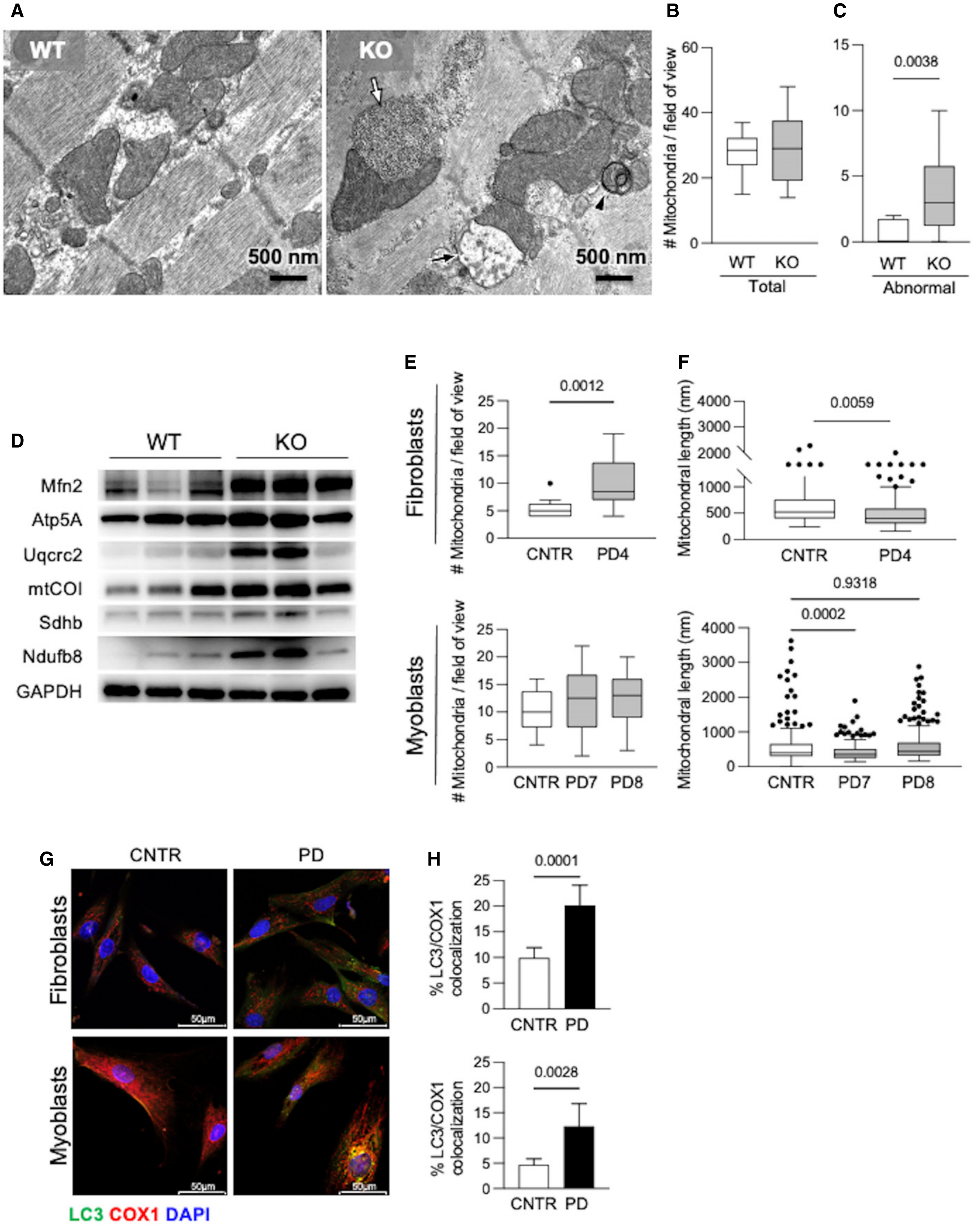
A
WT
500 nm
KO
500 nm
B
Mitochondria / field of view
0
20
40
60
WT KO
Total
C
0
5
10
15
0.0038
WT KO
Abnormal
D
WT
KO
Mfn2
Atp5A
Uqcrc2
mtCOI
Sdhb
Ndufb8
GAPDH
E
Fibroblasts
Mitochondria / field of view
0.0012
CNTR PD4
Myoblasts
CNTR PD7 PD8
F
Mitochondral length (nm)
0.0059
CNTR PD4
0.9318
0.0002
CNTR PD7 PD8
G
CNTR
PD
Fibroblasts
Myoblasts
50µm
LC3 COX1 DAPI
H
% LC3/COX1 colocalization
0.0001
CNTR PD
0.0028
CNTR PD

Figure EV1.

Figure EV2. Autophagy markers.

A Western blot analysis of p62 and LC3 in control (CNTR) (n = 2) and Pompe disease (PD) (n = 3) fibroblasts.
B Immunofluorescence analysis of LC3 in cultured CNTR and PD fibroblasts. Confocal 63× images; scale bar 50 μm; brightness +25%; contrast +20%.
C Western blot and quantitative analyses of autophagy marker LC3 in CNTR and in a PD cell line. The analysis was performed in untreated cells and after different treatments to modulate autophagy (starvation, STAR; rapamycin, RAPA; MK6-83; bafilomycin, BAFI).
D Western blot and quantitative analyses of autophagy marker LC3 in a PD cell line. The analysis was performed in untreated cells and after different treatments to induce stress (sodium arsenite, ARS; tert-butyl-peroxide, TBP).
E Western blot and quantitative analyses of autophagy marker LC3 in a PD cell line. The analysis was performed in untreated cells and after antioxidant treatments.

Source data are available online for this figure.

 © 2021 The Authors

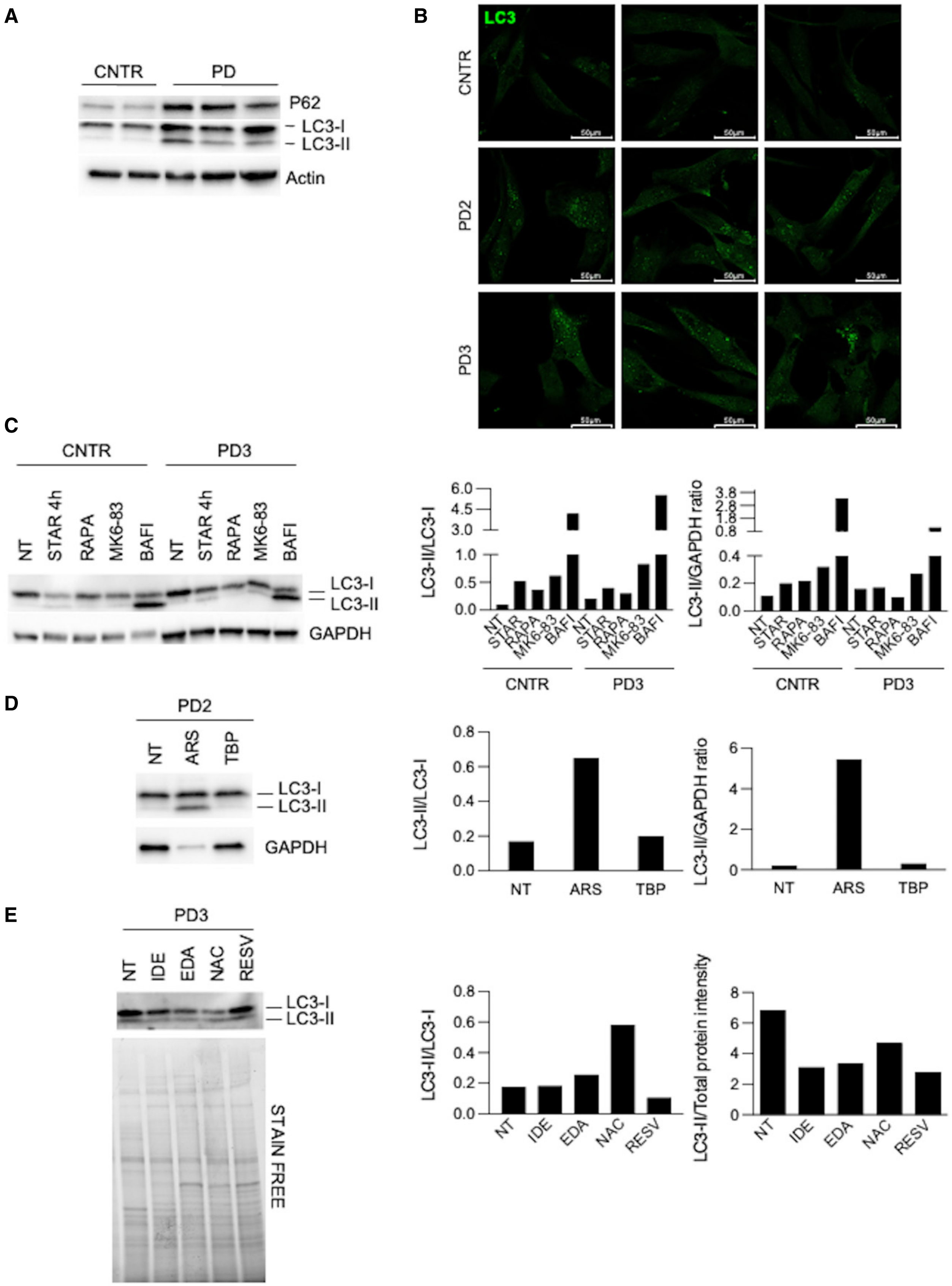
A
CNTR
PD
P62
LC3-I
LC3-II
Actin
B
LC3
CNTR
PD2
PD3
50µm
C
CNTR
PD3
NT
STAR 4h
RAPA
MK6-83
BAFI
LC3-I
LC3-II
GAPDH
LC3-II/LC3-I
LC3-II/GAPDH ratio
D
PD2
NT
ARS
TBP
LC3-I
LC3-II
GAPDH
E
PD3
NT
IDE
EDA
NAC
RESV
LC3-I
LC3-II
STAIN FREE
LC3-II/Total protein intensity

Figure EV2.

Figure EV3. Effects of antioxidants on GAA heat stability by DSF.

A Thermal scans of rhGAA in the presence of the pharmacological chaperone NAC and antioxidants. The table shows the melting temperatures and their relative shifts in the absence and in the presence of antioxidants.

B Determination of rhGAA-edaravone interaction by differential scanning fluorimetry. The table shows the melting temperatures and their relative shifts in absence and in the presence of increasing concentrations of edaravone. Data presented as mean ± SD of data obtained in three different PD fibroblast cell lines. In each cell line, the analysis was performed in triplicate.

Source data are available online for this figure.

© 2021 The Authors

A

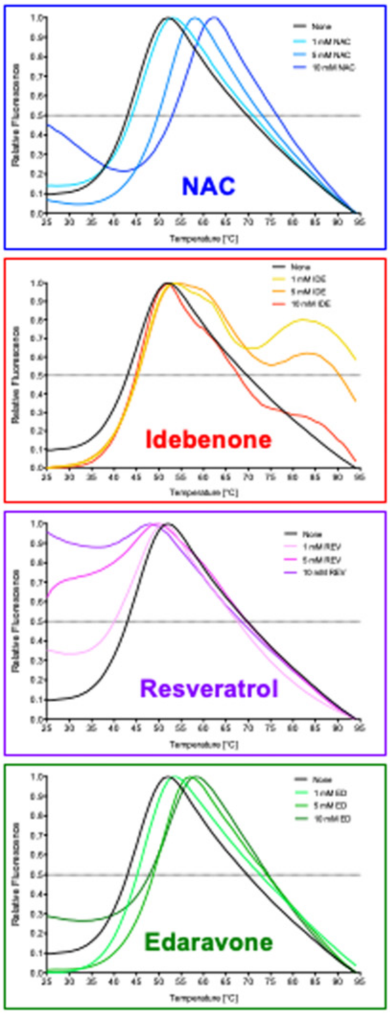

	NAC			
	None	1 mM	5 mM	10 mM
Tm	43.6±0.4	45.0±0.1	50.1±0.1	54.7±0.2
ΔTm	-	+1.4±0.5	+6.5±0.5	+11.1±0.6

	Idebenone			
	None	1 mM	5 mM	10 mM
Tm	43.6±0.4	45.3±0.1	45.3±0.1	45.0±0.4
ΔTm	-	+1.7±0.5	+1.7±0.5	+1.4±0.5

	Resveratrol			
	None	1 mM	5 mM	10 mM
Tm	43.6±0.4	43.3±0.1	42.0±0.7	41.9±1.4
ΔTm	-	+0.3±0.5	-1.6±1.1	-1.7±1.8

	Edaravone			
	None	1 mM	5 mM	10 mM
Tm	43.6±0.4	45.1±0.1	48.9±0.1	50.4±0.2
ΔTm	-	+1.5±0.5	+5.3±0.5	+6.8±0.6

B

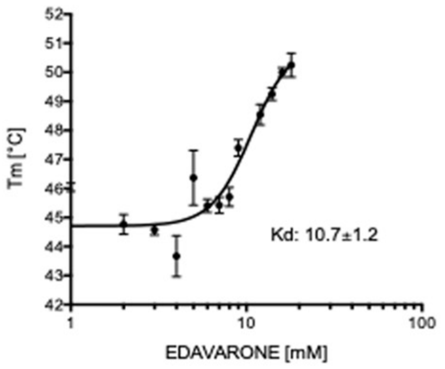

Max thermal shift: +5.94±0.62 °C

[mM] EDARAVONE	Tm (°C)	ΔTm (°C)
None	44.31±0.20	-
0.1	44.17±0.76	-0.14±0.95
0.5	44.64±0.07	0.33±0.28
1	46.04±0.14	1.73±0.34
2	44.77±0.34	0.46±0.54
3	44.58±0.18	0.27±0.39
4	43.67±0.71	-0.64±0.91
5	46.37±0.94	2.06±1.15
6	45.40±0.22	1.09±0.43
7	45.42±0.27	1.11±0.47
8	45.71±0.33	1.4±0.53
9	47.40±0.29	3.09±0.49
10	50.64±0.01	6.33±0.21
12	48.54±0.36	4.23±0.56
14	49.25±0.22	4.94±0.42
16	49.99±0.16	5.68±0.36
18	50.25±0.41	5.94±0.62

Figure EV3.

© 2021 The Authors

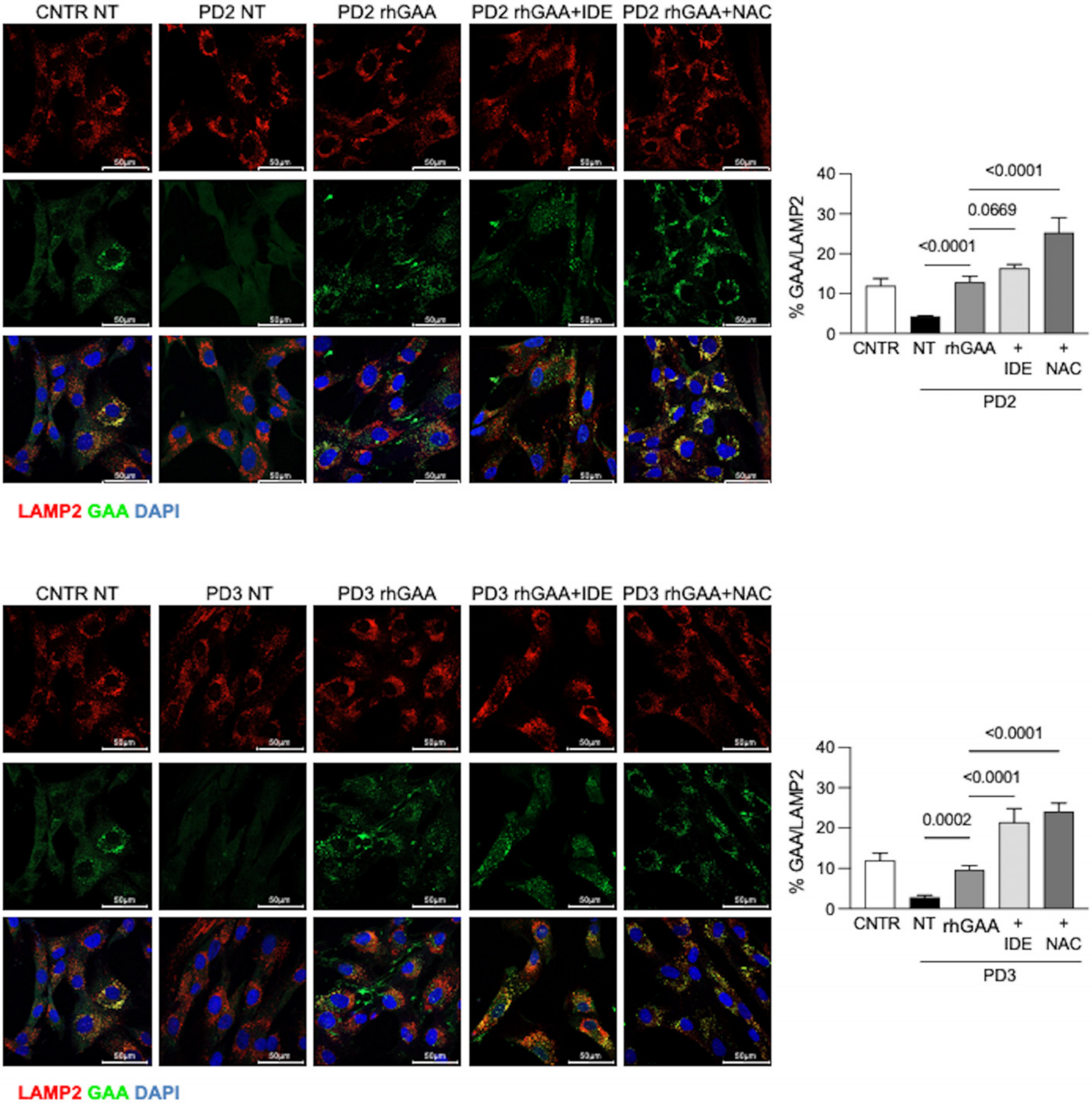

Figure EV4. Immunofluorescence analysis of GAA-Lamp2 in cells treated with rhGAA alone and in combination with antioxidants.

Confocal immunofluorescence analysis of GAA and LAMP2 in PD fibroblasts (PD2, PD3) and respective percent of GAA/LAMP2 colocalization. Fibroblasts were treated with rhGAA in the absence and in the presence of IDE or NAC. Control fibroblasts from Fig 5E and untreated PD cells are shown for comparison.

Data information: Data presented as mean ± SD of five images for each condition in each patient. To calculate statistical significance, one-way ANOVA was applied for all experiments followed by Dunnett's test. Statistically significant *P* values are indicated. Confocal 63× images; scale bar 50 μm; brightness +20%.

Source data are available online for this figure.

 © 2021 The Authors

Figure EV5. Effects of autophagy induction on correction of GAA activity by rhGAA and M6PR localization at the plasma membrane.

A Relative GAA activity increase in PD fibroblasts (cell lines $n = 3$) treated with rhGAA alone and with rhGAA in combination with autophagy activators (starvation, STAR; rapamycin, RAPA; MK6-83; ML-SA1). The effects of rhGAA alone are taken as 100%. The results are expressed as means ± SD. ANOVA was applied followed by Dunnett's multicomparison test.

B Western blot analysis of GAA isoforms and quantitative analysis of the different enzyme isoforms (top) normalized to stain free (middle). All drugs improved the amounts of rhGAA-related polypeptides and the processing of rhGAA into the active isoforms (76–70 kDa). The image shown is representative of at least three independent experiments in different PD patients.

C FACS analysis of control and PD fibroblast shows the M6PR amount at plasma membrane before and after treatments with autophagy activators (rapamycin and torin1).

D Mean of results obtained in control ($n = 2$ cell lines) and PD fibroblast ($n = 3$ cell lines). For each cell line, the amount of M6PR-positive cells was normalized, taking that observed in non-treated fibroblasts as 1. Data presented as mean ± SD. ANOVA was applied followed by Sidak's multicomparison test.

Source data are available online for this figure.

© 2021 The Authors

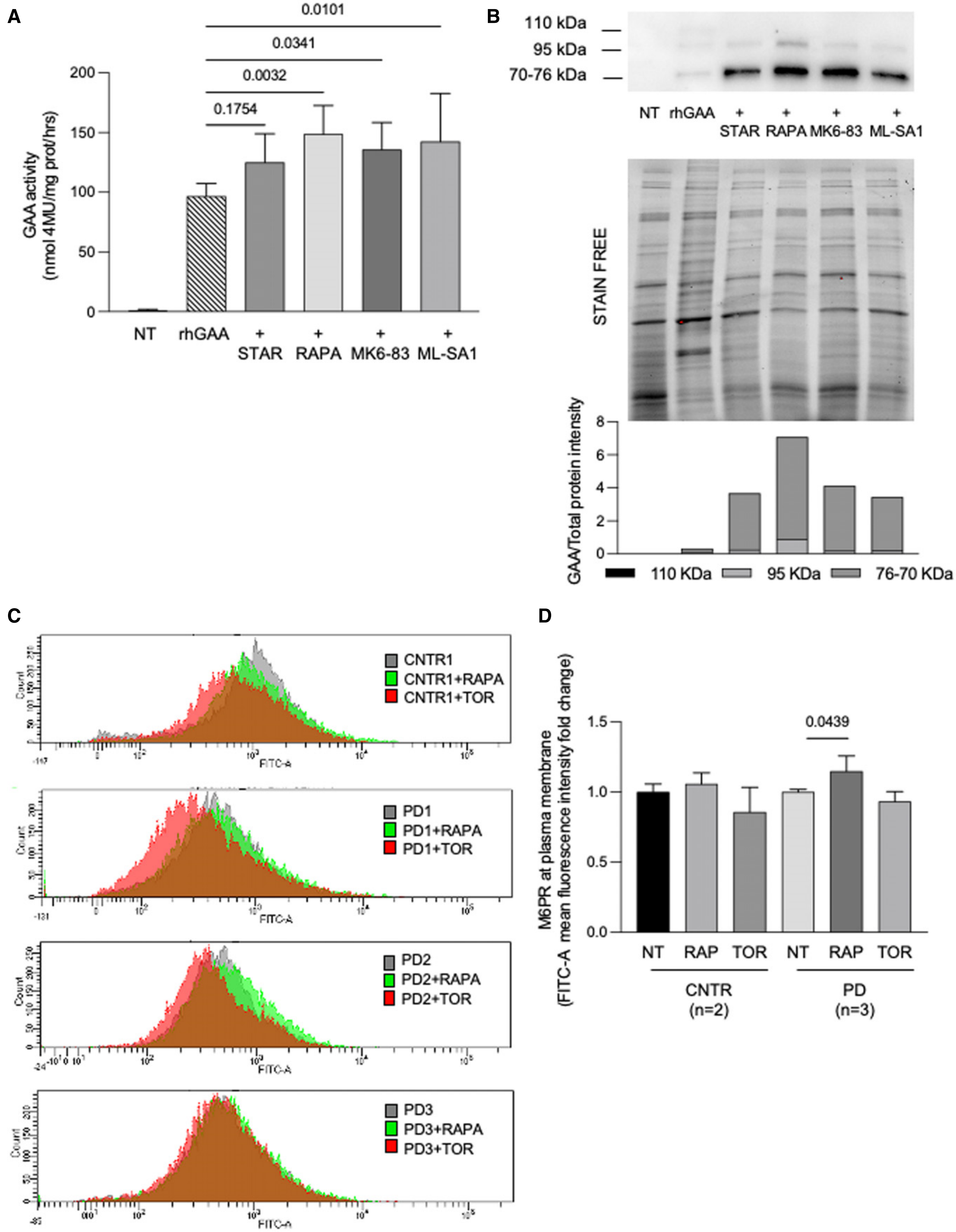

Figure EV5.

© 2021 The Authors
