# Supplementary material for: Correction of oxidative stress enhances enzyme replacement therapy in Pompe disease
Source: EMBO Mol Med. 2021 Oct 4;13(11):e14434. doi: 10.15252/emmm.202114434 (PMC8573602; doi:10.15252/emmm.202114434)
Supplement: Supplementary file 3 — Source Data for Expanded View [file EMMM-13-e14434-s007.zip › SourceDataForExpandedView/SourceDataForExpandedView5/FigEV5-WB.pdf]

Figure EV5-Effects of autophagy induction on correction of GAA activity by rhGAA and M6PR localization at the plasma membrane

B

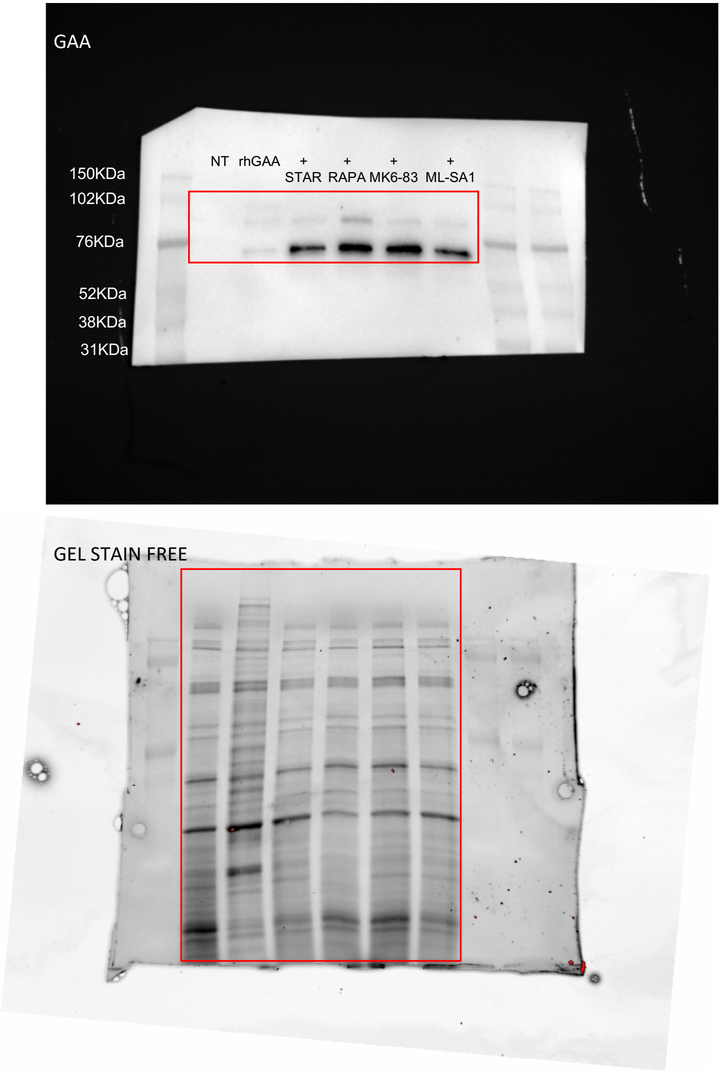

Amersham Rainbow Marker
anti-GAA, PRIMM, MA, 1:500
