# Supplementary material for: Correction of oxidative stress enhances enzyme replacement therapy in Pompe disease
Source: EMBO Mol Med. 2021 Oct 4;13(11):e14434. doi: 10.15252/emmm.202114434 (PMC8573602; doi:10.15252/emmm.202114434)
Supplement: Supplementary file 3 — Source Data for Expanded View [file EMMM-13-e14434-s007.zip › SourceDataForExpandedView/SourceDataForExpandedView2/FigEV2-WB.pdf]

Figure EV2- Autophagy markers.

A

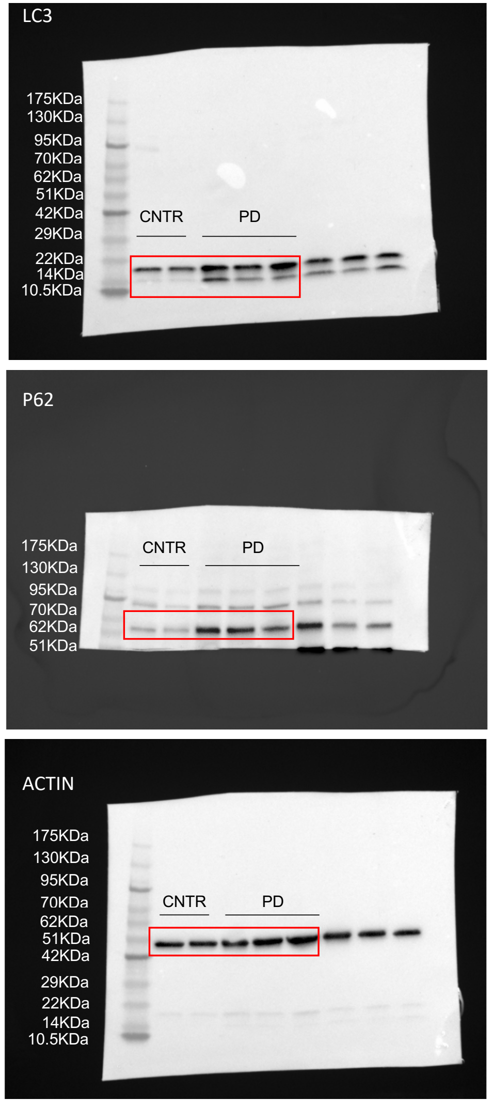

Opti-Protein Marker/Ladder
anti-LC3, Novus Biologicals, Littleton, CO, USA, 1:500
anti-p62, Sigma-Aldrich, St. Louis, MO, USA, 1:1000
anti-Actin, Sigma-Aldrich, St. Louis, MO, USA, 1:2000

C

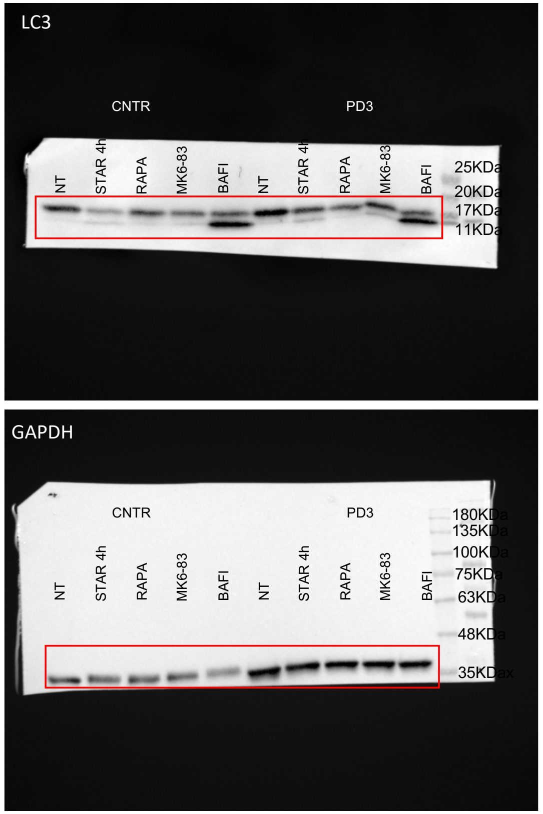

Opti-Protein XL Marker/Ladder
anti-LC3, Novus Biologicals, Littleton, CO, USA, 1:500
anti-GAPDH, Ambion, Austin. TX, USA, 1:2000

D

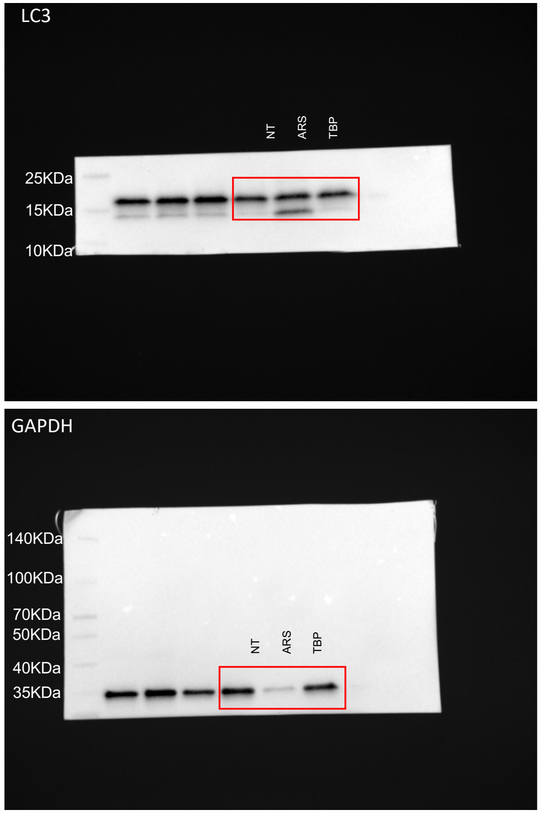

Spectra Multicolor Broad Range Protein Ladder
anti-LC3, Novus Biologicals, Littleton, CO, USA, 1:500
anti-GAPDH, Ambion, Austin. TX, USA, 1:2000

E

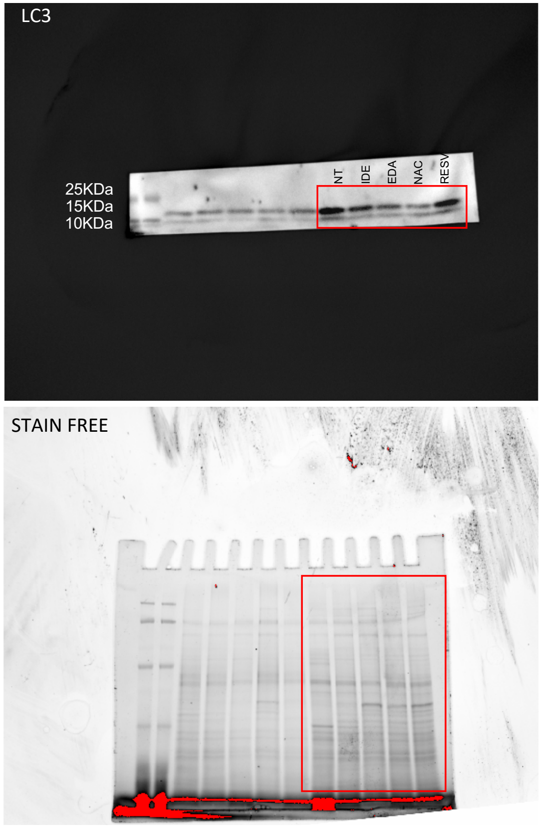

Spectra Multicolor Broad Range Protein Ladder
anti-LC3, Novus Biologicals, Littleton, CO, USA, 1:500
