# Supplementary material for: Correction of oxidative stress enhances enzyme replacement therapy in Pompe disease
Source: EMBO Mol Med. 2021 Oct 4;13(11):e14434. doi: 10.15252/emmm.202114434 (PMC8573602; doi:10.15252/emmm.202114434)
Supplement: Supplementary file 3 — Source Data for Expanded View [file EMMM-13-e14434-s007.zip › SourceDataForExpandedView/SourceDataForExpandedView1/FigEV1-WB.pdf]

Figure EV1 - Characterization of mitochondria in gastrocnemii from the PD mouse model and in cultured PD patient cells

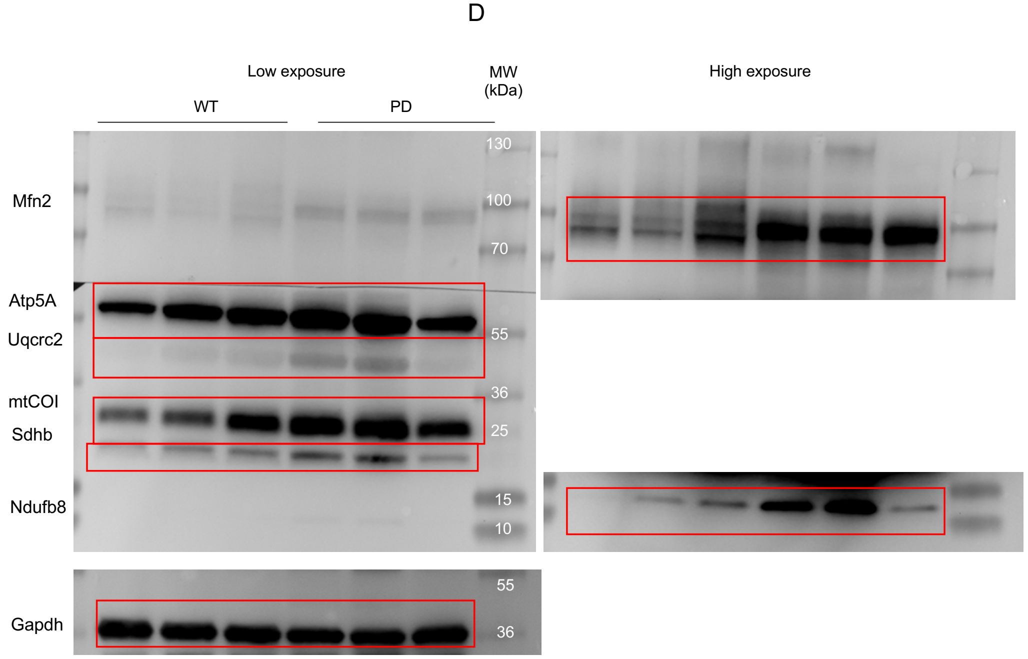

total OXPHOS Rodent WB, Abcam, Cambridge, UK,1:250
anti-Mfn2, Abcam, Cambridge, UK,1:1000
anti-GAPDH, Ambion, Austin. TX, USA,1:2000
