# Supplementary material for: Correction of oxidative stress enhances enzyme replacement therapy in Pompe disease
Source: EMBO Mol Med. 2021 Oct 4;13(11):e14434. doi: 10.15252/emmm.202114434 (PMC8573602; doi:10.15252/emmm.202114434)
Supplement: Supplementary file 4 — Source Data for Figure 1 [file EMMM-13-e14434-s009.zip › SourceDataForFigur1/Fig1.pdf]

Figure 1-Increased oxidative stress in PD

B

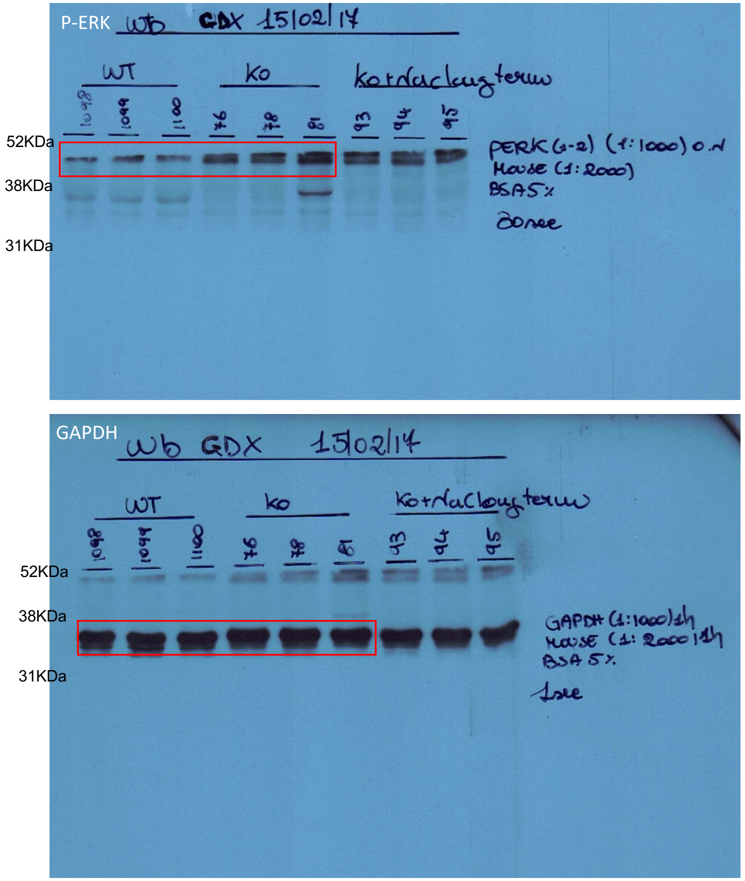

Amersham Rainbow Marker
anti-phospho ERK, Cell Signaling, Danvers, MA, USA, 1:1000
anti-GAPDH, Ambion, Austin. TX, USA, 1:2000

D

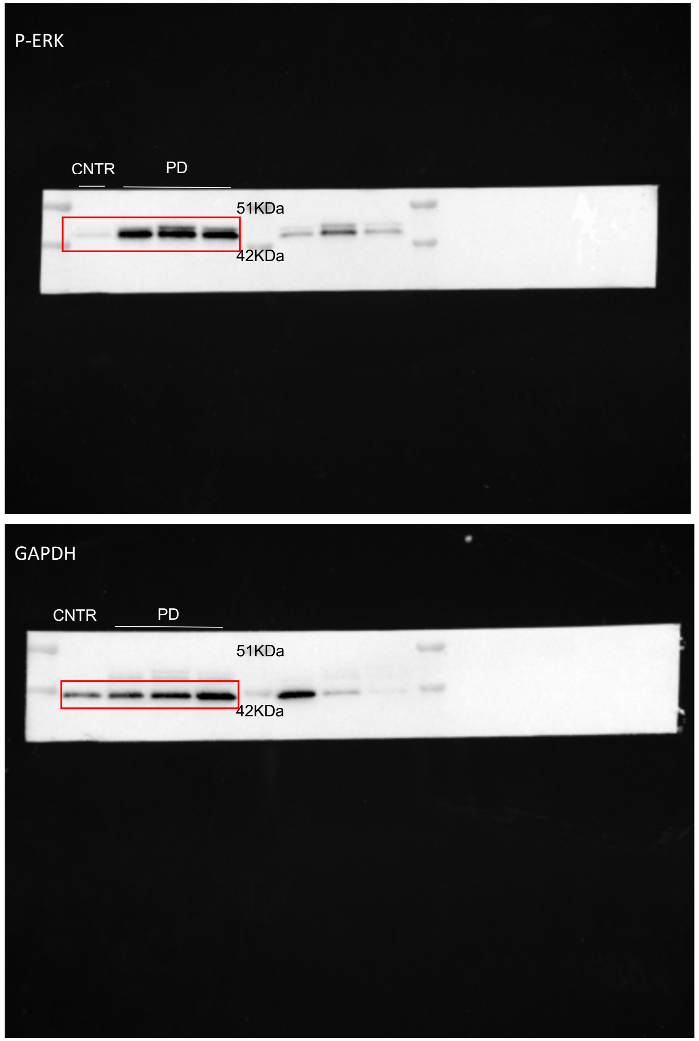

Opti-Protein Marker/Ladder
anti-phospho ERK, Cell Signaling, Danvers, MA, USA, 1:1000
anti-GAPDH, Ambion, Austin. TX, USA, 1:2000

F

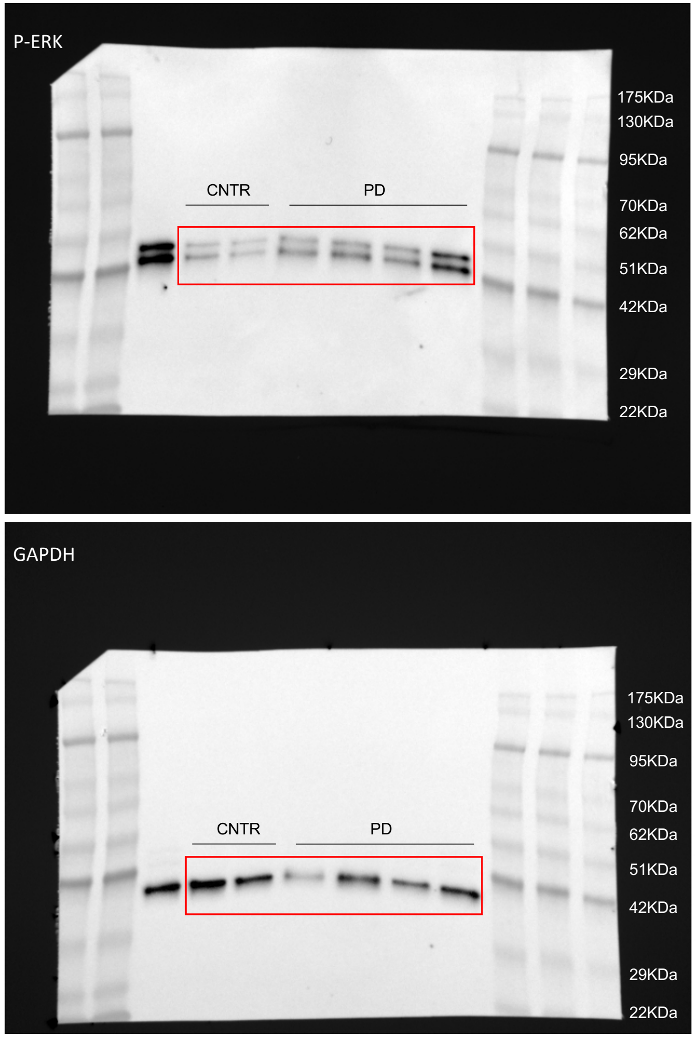

Opti-Protein Marker/Ladder
anti-phospho ERK, Cell Signaling, Danvers, MA, USA, 1:1000
anti-GAPDH, Ambion, Austin. TX, USA, 1:2000

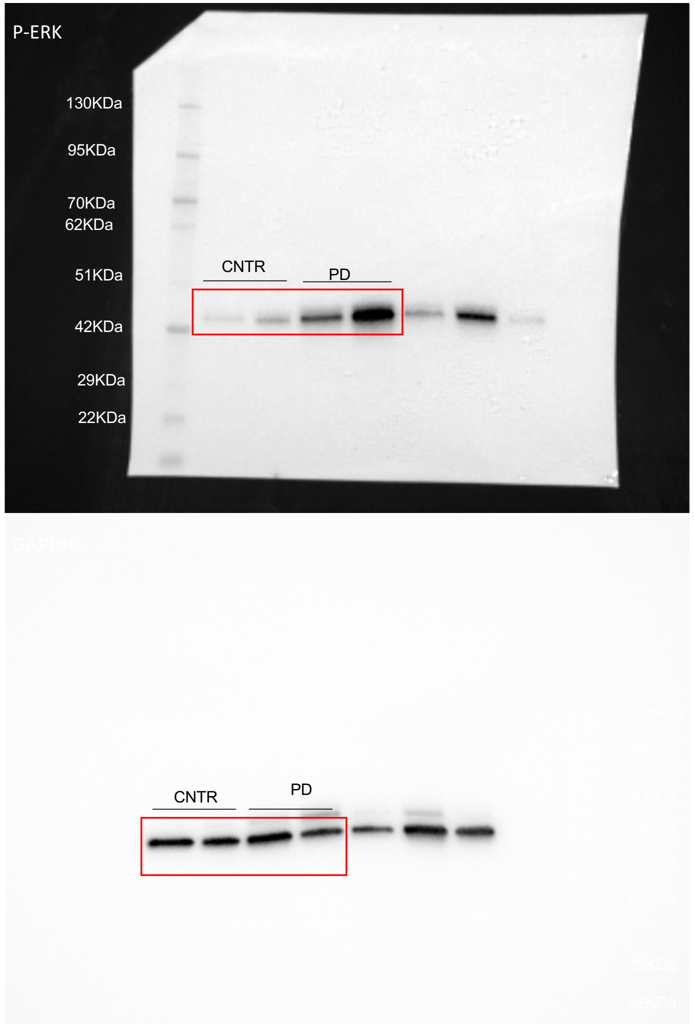

Opti-Protein Marker/Ladder
anti-phospho ERK, Cell Signaling, Danvers, MA, USA, 1:1000
anti-GAPDH, Ambion, Austin. TX, USA, 1:2000
