# Supplementary material for: Correction of oxidative stress enhances enzyme replacement therapy in Pompe disease
Source: EMBO Mol Med. 2021 Oct 4;13(11):e14434. doi: 10.15252/emmm.202114434 (PMC8573602; doi:10.15252/emmm.202114434)
Supplement: Supplementary file 5 — Source Data for Figure 2 [file EMMM-13-e14434-s001.zip › SourceDataForFigur2/Fig2.pdf]

Figure 2-Modulation of autophagy impacts on stress in PD fibroblasts

A

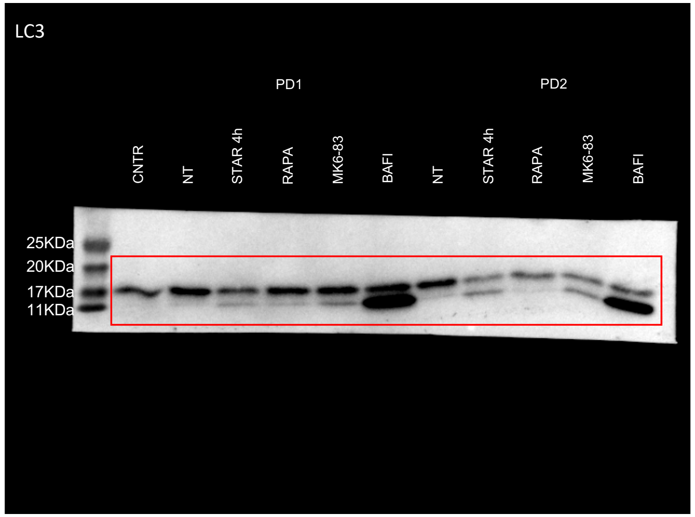

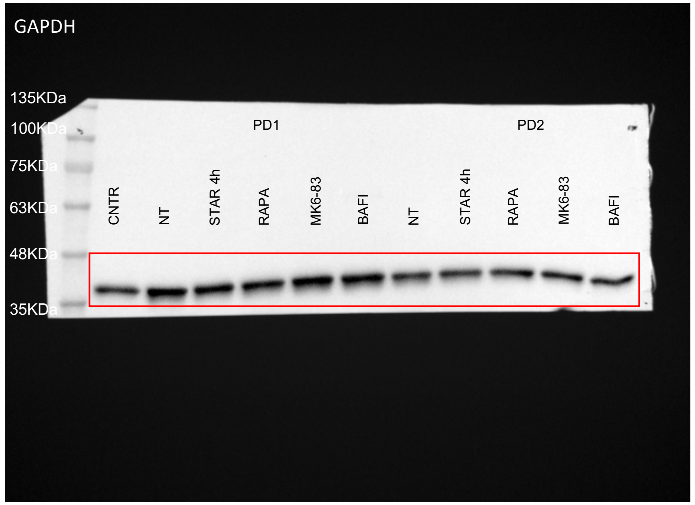

Opti-Protein XL Marker/Ladder
anti-LC3, Novus Biologicals, Littleton, CO, USA, 1:500
anti-GAPDH, Ambion, Austin. TX, USA, 1:2000

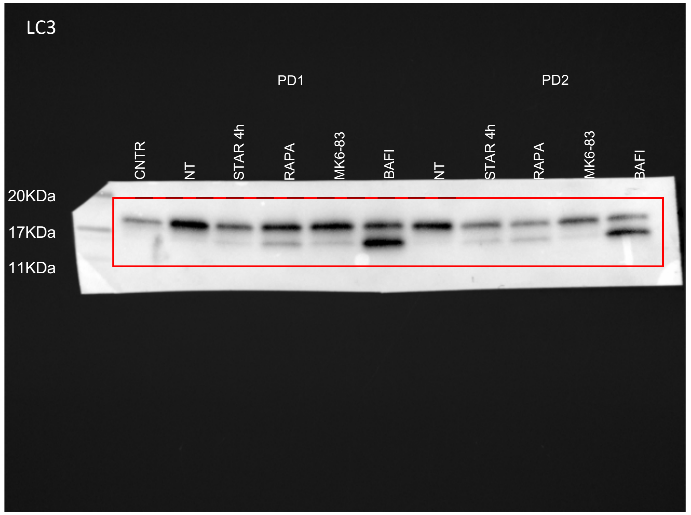

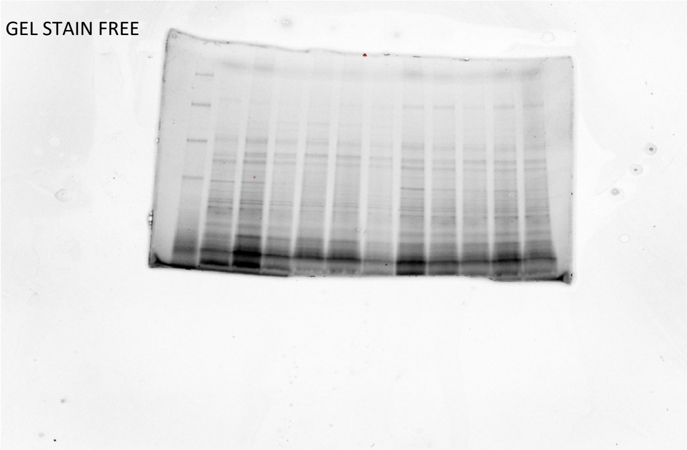

Opti-Protein XL Marker/Ladder
anti-LC3, Novus Biologicals, Littleton, CO, USA, 1:500
