# Supplementary material for: Correction of oxidative stress enhances enzyme replacement therapy in Pompe disease
Source: EMBO Mol Med. 2021 Oct 4;13(11):e14434. doi: 10.15252/emmm.202114434 (PMC8573602; doi:10.15252/emmm.202114434)
Supplement: Supplementary file 7 — Source Data for Figure 4 [file EMMM-13-e14434-s003.zip › SourceDataForFigur4/Fig4.pdf]

Figure 4-Induction of oxidative stress affect rhGAA uptake/processing

4D

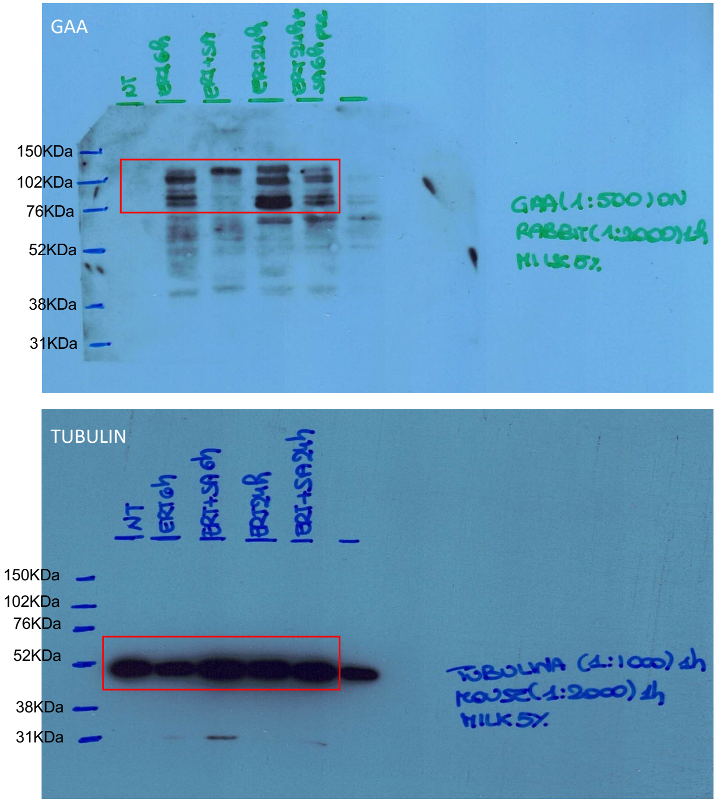

Amersham Rainbow Marker
anti-GAA, PRIMM, MA, 1:500
anti-tubulin, Sigma-Aldrich, St. Louis, MO, USA, 1:2000

4F

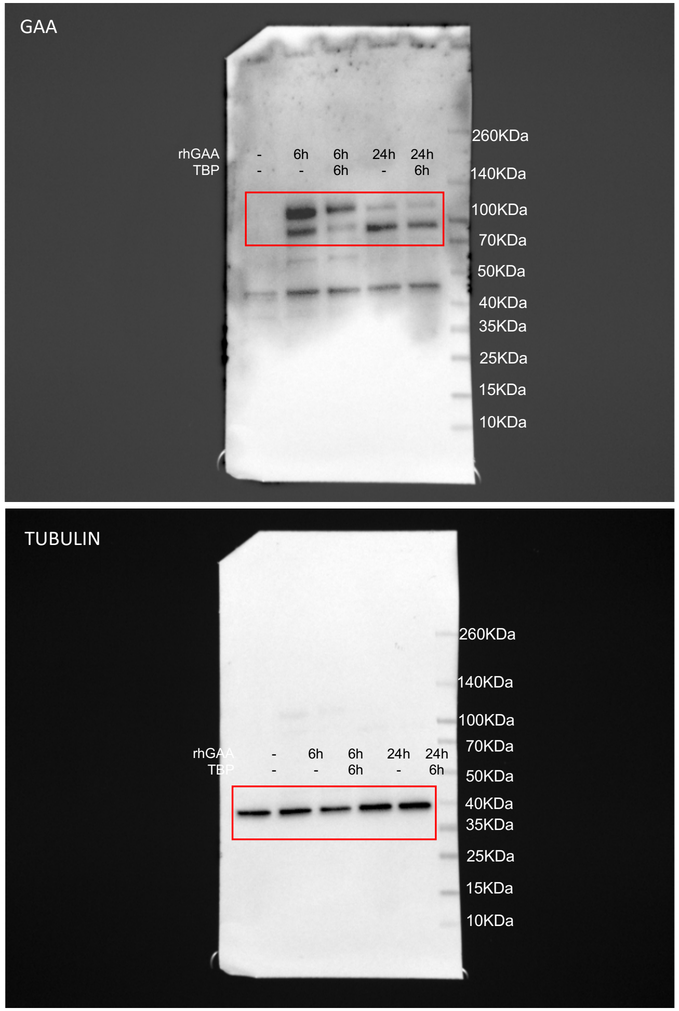

Spectra Multicolor Broad Range Protein Ladder
anti-GAA, PRIMM, MA, 1:500
anti-tubulin, Sigma-Aldrich, St. Louis, MO, USA, 1:2000
