# Supplementary material for: Correction of oxidative stress enhances enzyme replacement therapy in Pompe disease
Source: EMBO Mol Med. 2021 Oct 4;13(11):e14434. doi: 10.15252/emmm.202114434 (PMC8573602; doi:10.15252/emmm.202114434)
Supplement: Supplementary file 8 — Source Data for Figure 5 [file EMMM-13-e14434-s004.zip › SourceDataForFigur5/Fig5.pdf]

Figure 5-Effect of antioxidants on stress and ERT

5C

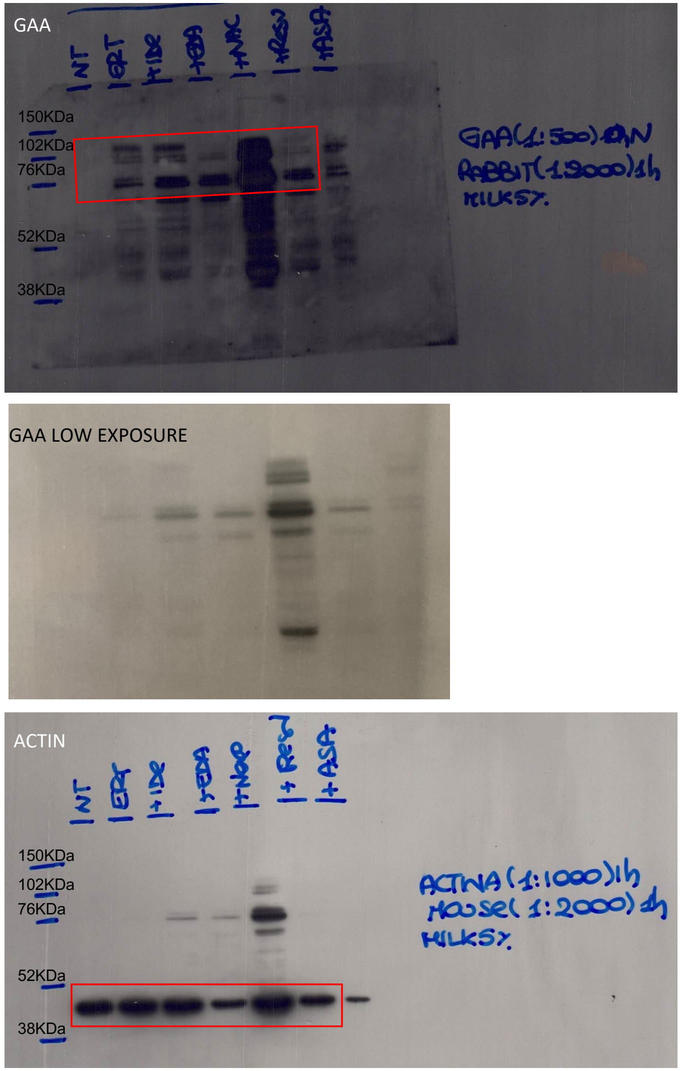

Amersham Rainbow Marker
anti-GAA, PRIMM, MA, 1:500
anti-Actin, Sigma-Aldrich, St. Louis, MO, USA, 1:2000
