# Supplementary material for: Correction of oxidative stress enhances enzyme replacement therapy in Pompe disease
Source: EMBO Mol Med. 2021 Oct 4;13(11):e14434. doi: 10.15252/emmm.202114434 (PMC8573602; doi:10.15252/emmm.202114434)
Supplement: Supplementary file 10 — Source Data for Figure 7 [file EMMM-13-e14434-s002.zip › SourceDataForFigur7/Fig7.pdf]

Figure 7-Effect of antioxidants in PD mouse

7E IDE

Diaphragm

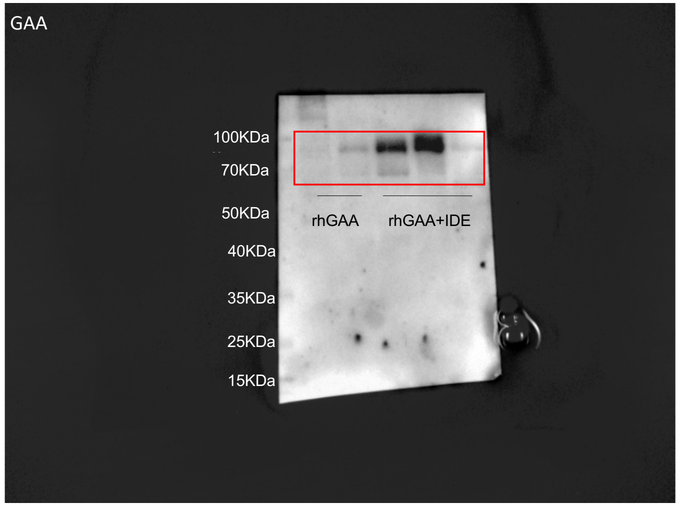

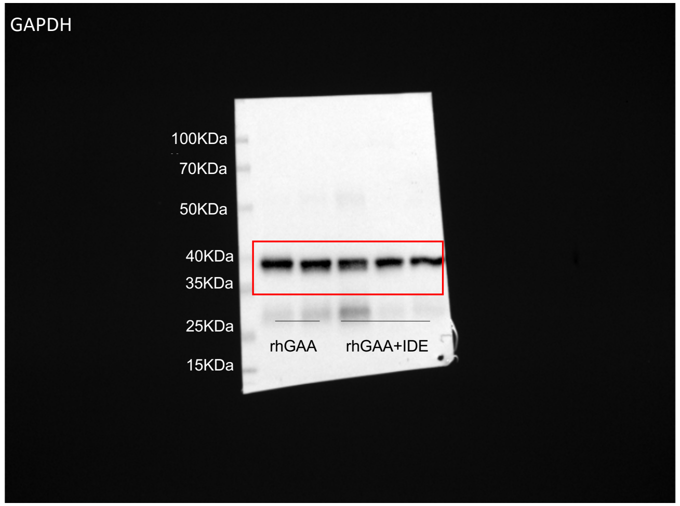

Spectra Multicolor Broad Range Protein Ladder
anti-GAA, PRIMM, MA, 1:500
anti-GAPDH, Ambion, Austin. TX, USA, 1:2000

Heart

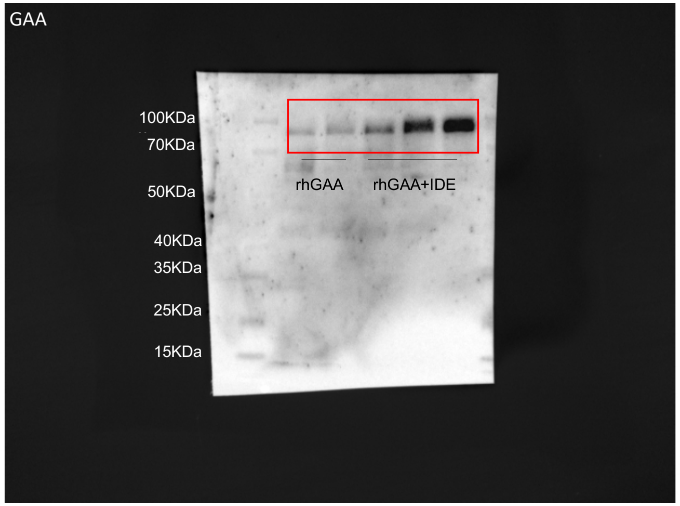

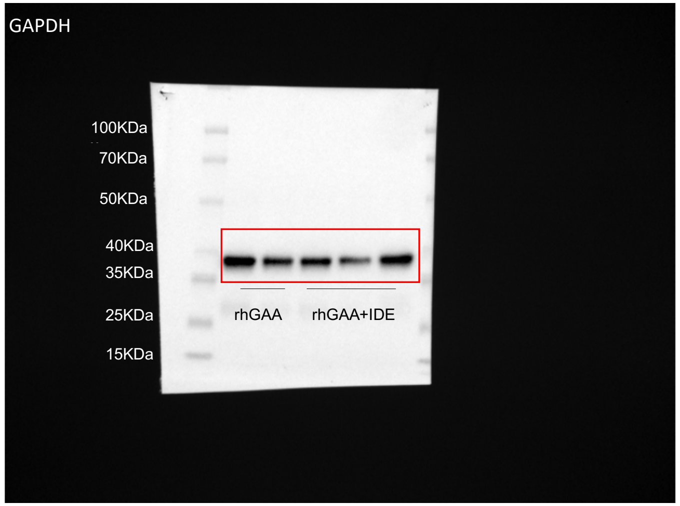

Spectra Multicolor Broad Range Protein Ladder
anti-GAA, PRIMM, MA, 1:500
anti-GAPDH, Ambion, Austin. TX, USA, 1:2000

Liver

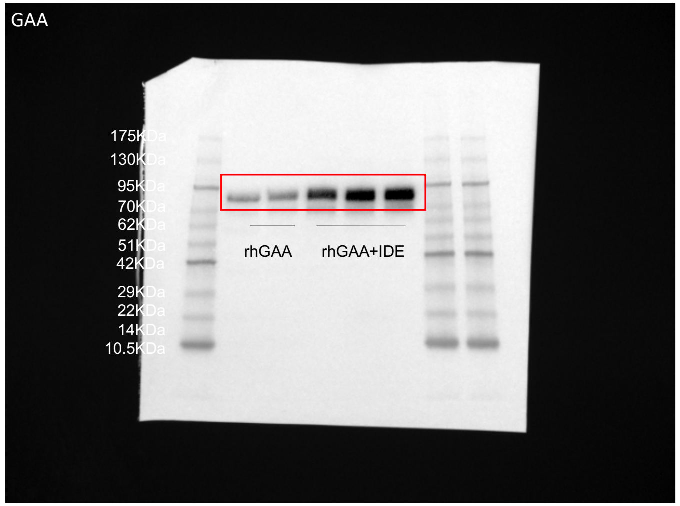

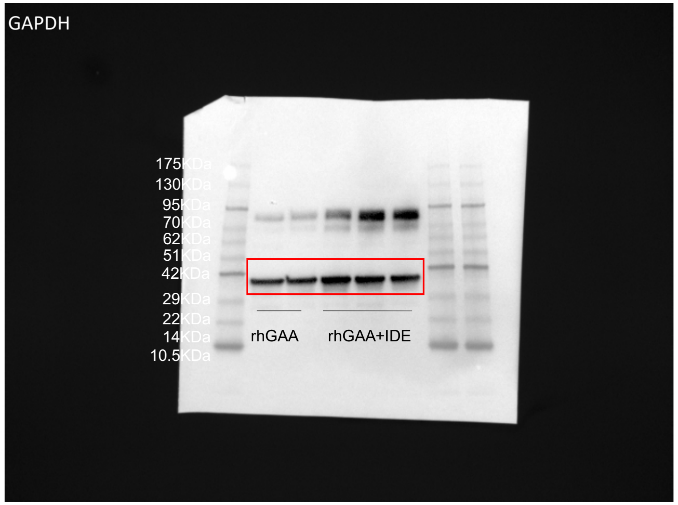

Opti-Protein Marker/Ladder
anti-GAA, PRIMM, MA, 1:500
anti-GAPDH, Ambion, Austin. TX, USA, 1:2000

QD

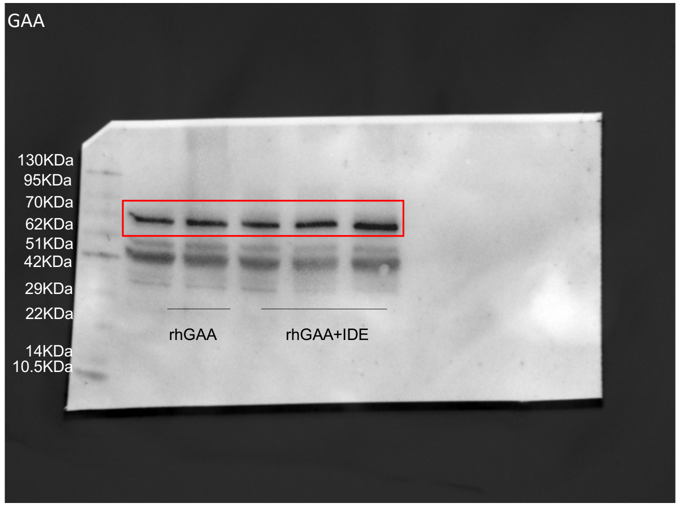

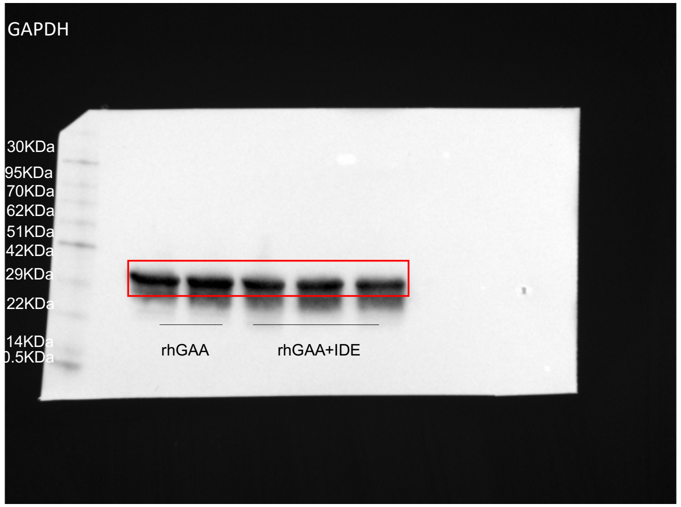

Opti-Protein Marker/Ladder
anti-GAA, PRIMM, MA, 1:500
anti-GAPDH, Ambion, Austin. TX, USA, 1:2000

GS

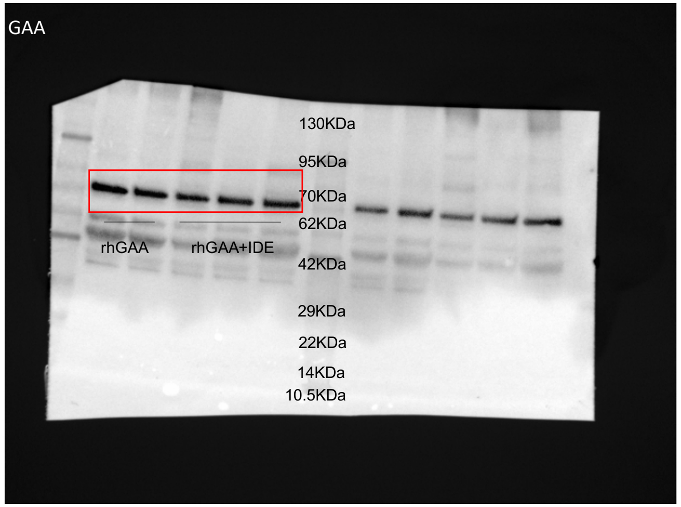

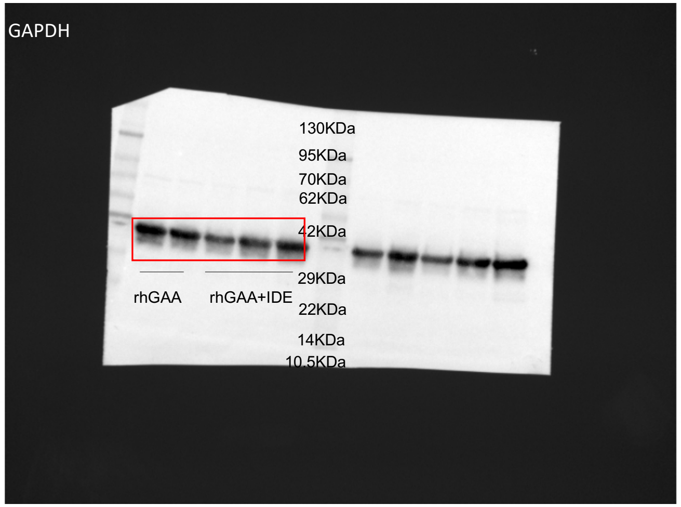

Opti-Protein Marker/Ladder
anti-GAA, PRIMM, MA, 1:500
anti-GAPDH, Ambion, Austin. TX, USA, 1:2000

7F NAC
Diaphragm

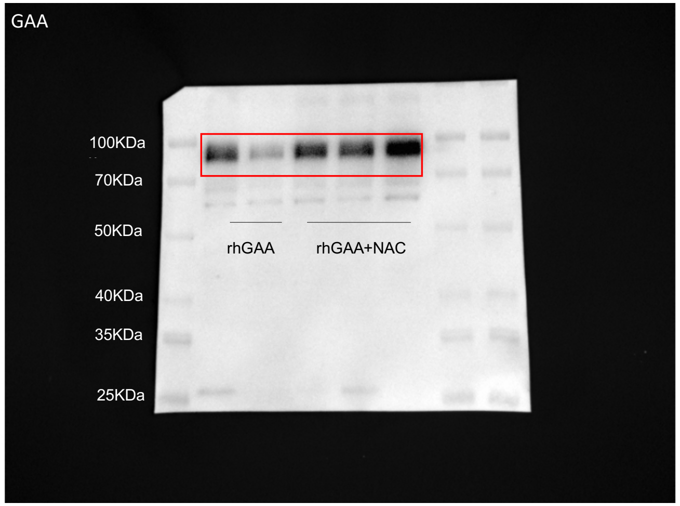

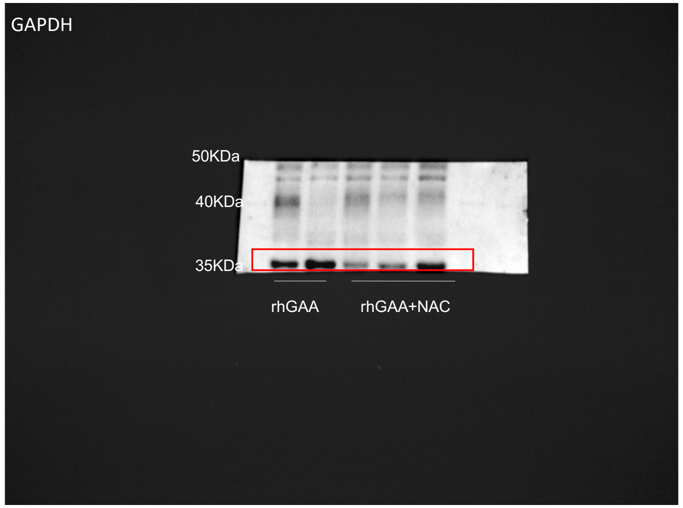

Spectra Multicolor Broad Range Protein Ladder
anti-GAA, PRIMM, MA, 1:500
anti-GAPDH, Ambion, Austin. TX, USA, 1:2000

Heart

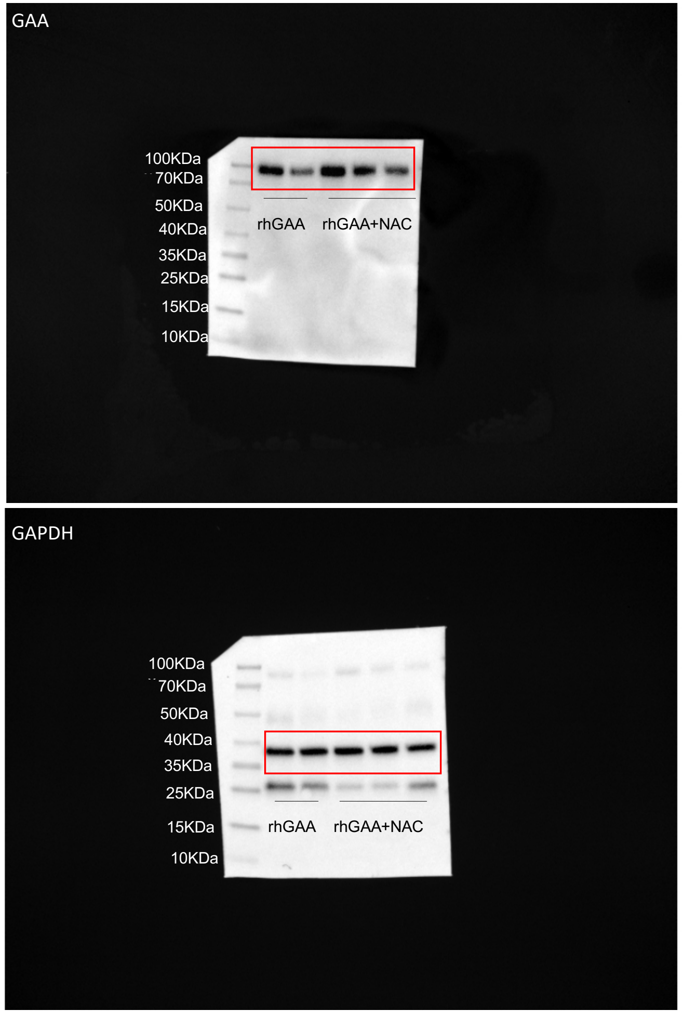

Spectra Multicolor Broad Range Protein Ladder
anti-GAA, PRIMM, MA, 1:500
anti-GAPDH, Ambion, Austin. TX, USA, 1:2000

Liver

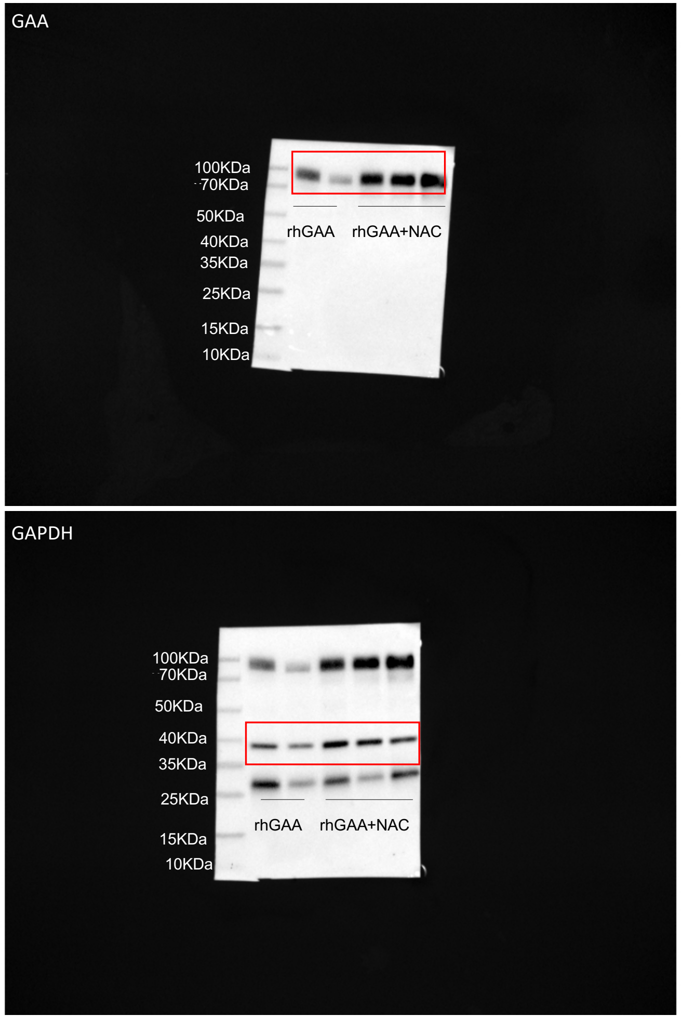

Spectra Multicolor Broad Range Protein Ladder
anti-GAA, PRIMM, MA, 1:500
anti-GAPDH, Ambion, Austin. TX, USA, 1:2000

QD

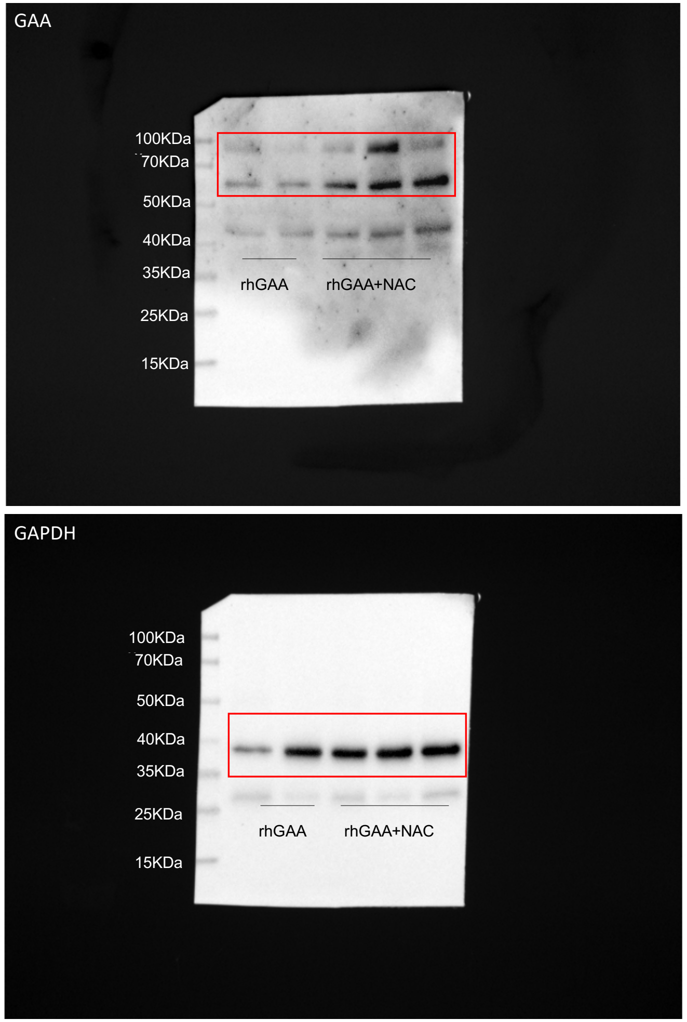

Spectra Multicolor Broad Range Protein Ladder
anti-GAA, PRIMM, MA, 1:500
anti-GAPDH, Ambion, Austin. TX, USA, 1:2000

GS

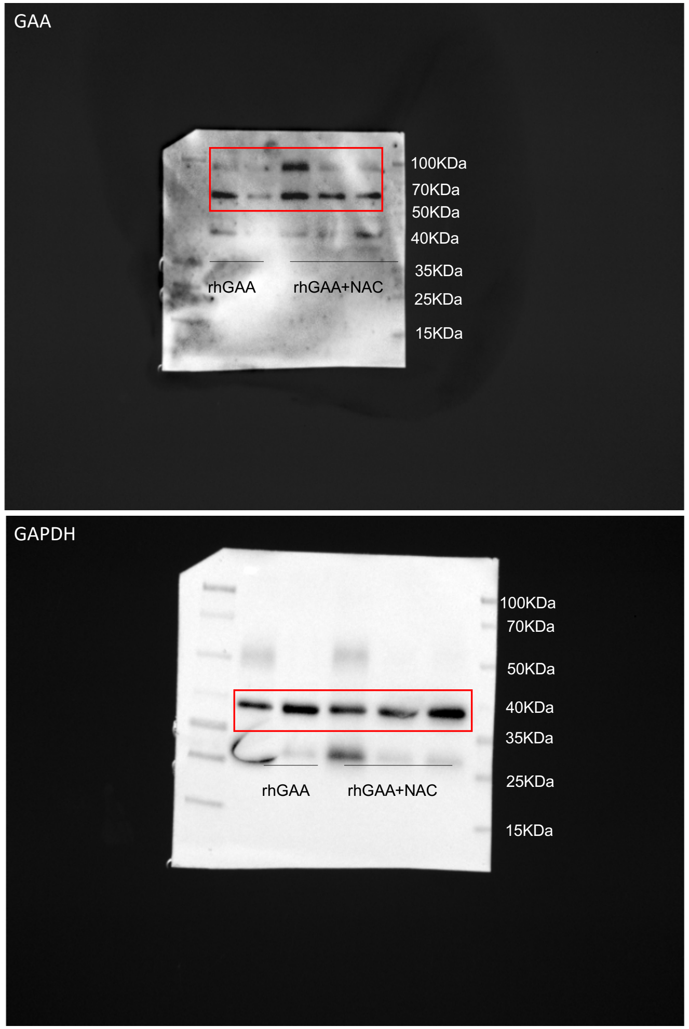

Spectra Multicolor Broad Range Protein Ladder
anti-GAA, PRIMM, MA, 1:500
anti-GAPDH, Ambion, Austin. TX, USA, 1:2000
