# Supplementary material for: Correction of oxidative stress enhances enzyme replacement therapy in Pompe disease
Source: EMBO Mol Med. 2021 Oct 4;13(11):e14434. doi: 10.15252/emmm.202114434 (PMC8573602; doi:10.15252/emmm.202114434)
Supplement: Supplementary file 11 [file EMMM-13-e14434-s005.pdf]

EMBO Molecular Medicine

Correction of oxidative stress enhances enzyme replacement therapy in Pompe disease

Antonietta Tarallo, Carla Damiano, Sandra Strollo, Nadia Minopoli, Alessia Indrieri, Elena Polishchuk, Francesca Zappa, Edorado Nusco, Simona Fecarotta, Caterina Porto, Marcella Coletta, Roberta Iacono, Marco Moracci, Roman Polishchuk, Diego Medina, Paola Imbimbo, Daria Monti, Maria Antonietta De Matteis, and Giancarlo Parenti
DOI: 10.15252/emmm.202114434

Corresponding author: Giancarlo Parenti (parenti@tigem.it)

Review Timeline:

Submission Date:	15th Apr 21
Editorial Decision:	4th May 21
Revision Received:	30th Jul 21
Editorial Decision:	13th Aug 21
Revision Received:	15th Sep 21
Accepted:	20th Sep 21

Editor: Zeljko Durdevic

Transaction Report:

(Note: With the exception of the correction of typographical or spelling errors that could be a source of ambiguity, letters and reports are not edited. Depending on transfer agreements, referee reports obtained elsewhere may or may not be included in this compilation. Referee reports are anonymous unless the Referee chooses to sign their reports.)

1st Editorial Decision 4th May 2021

Dear Prof. Parenti,

Thank you for the submission of your manuscript to EMBO Molecular Medicine. We have now received feedback from the three reviewers who agreed to evaluate your manuscript. As you will see from the reports below, the referees acknowledge the interest of the study but also raise serious and partially overlapping concerns that should be addressed in a major revision. The focus of the revision should be on improving the technical quality of the study, repeating statistical analysis using appropriate statistical tests, addressing the status of autophagy and its effects on the M6PR and GAA uptake.

Further consideration of a revision that addresses reviewers' concerns in full will entail a second round of review. EMBO Molecular Medicine encourages a single round of revision only and therefore, acceptance or rejection of the manuscript will depend on the completeness of your responses included in the next, final version of the manuscript. For this reason, and to save you from any frustrations in the end, I would strongly advise against returning an incomplete revision.

We would welcome the submission of a revised version within three months for further consideration. However, we realize that the current situation is exceptional on the account of the COVID-19/SARS-CoV-2 pandemic. Please let us know if you require longer to complete the revision.

I look forward to receiving your revised manuscript.

Yours sincerely,

Zeljko Durdevic

Zeljko Durdevic
Editor
EMBO Molecular Medicine

***** Reviewer's comments *****

Referee #1 (Remarks for Author):

In the current manuscript Tarallo and colleagues tested whether oxidative stress impacts on enzyme replacement therapy with recombinant human alpha-glucosidase (rhGAA), and whether correction of oxidative stress may be beneficial for rhGAA therapy. They found elevated oxidative stress levels in tissues from the Pompe disease murine model and in patients' cells. In cells, stress levels inversely correlated with the ability of rhGAA to correct the enzymatic deficiency. Antioxidants (N-acetylcysteine, idebenone, resveratrol, edaravone) improved enzymatic activity in

rhGAA-treated cells, enhanced enzyme processing, and improved mannose-6-phosphate receptor localization. When co-administered with rhGAA, antioxidants improved alpha-glucosidase activity in tissues from the Pompe disease mouse model. These results indicate that oxidative stress impacts on the efficacy of enzyme replacement therapy in Pompe disease, and that manipulation of secondary abnormalities may represent a strategy to improve the efficacy of therapies for this disorder.
I compliment the authors to the very interesting data, which are novel and very important not only for Pompe disease, but also other LSDs, which are also treated by enzyme replacement therapy. The fact that oxidative stress needs to be reduced to improve recombinant enzyme uptake could have a major clinical impact for future treatment strategies. Furthermore, the structure of the research is very strict and there is always a red line visible.

However, there are some major concerns, which need to be addressed.
General:
A references list is missing. Thus, there was no possibility to cross check citations etc., which is usually a no go. Furthermore, there are many typos within the manuscript and graphical data presentation is poor. Do all authors read and controlled the final version of the manuscript, which is in their responsibility?
However, since data and experiments are convincing in general and novel, the above referred concerns could be addressed within a major revision.
Furthermore, unfortunately applied statistics are wrong in most cases, which need to be revised and might have a major impact on the outcome.
Regarding the most important major concerns despite from the references and typos, please find my comments as follows:
Methodology:
Provide final concentrations for the antibodies instead of dilutions, since dilutions are not representative and not appropriate. Since antibody concentrations differ from lot to lot, antibody concentrations used for western blots etc. need to be provided as µg/ml for example. This point cannot be discussed. Provided data are too important in that other groups struggle over dilutions for the used antibodies, when transferring the knowledge into their own labs.
Provide complete producer information for the anti-GAA antibody.
Statistics:
If more than TWO columns are tested from ONE experiment, the Student's T test is NOT allowed. Instead, an ANOVA test with subsequent post-hoc analysis needs to be performed to correct for multiple testing. In detail, revise statistics for all experiments in Figure 2, Figure 4 A and C, Figure 5 A, B and D, Figure 6 B, C, G, Figure 7 C, Figure EV2 B, Figure EV3 A below; Figure EV7, Figure EV8 A. After using the appropriate statistical tool, authors might recognize that some outcomes are not significant any more. If a trend is visible, authors are free to perform additional experiments to raise the N. If not, data need to be re-evaluated and discussed.

Graphical layout:
Current figures are looking very poor and do not correspond to the comprehensive experiments and convincing data, which is too bad. Please revise ALL figures and refrain from using poor standard Excel graphics. For instance, use GRAPHPAD Prism for charts. Furthermore, delete the alpha channel in the figures to get rid of the edges around some charts.

Referee #2 (Remarks for Author):

Comment

It is an interesting study. The result may help the treatment of Pompe disease (PD) by enzyme replacement therapy. Current ERT only partially correct the function of patients with a severe form, infantile onset, of the disease. There are several questions about the data:

Figure 1 -.

Figure 1/2 - the authors show that PD cells are with increased oxidative stress. Enhancement of autophagy (starvation, rapamycin) decrease the oxidative stress, while (Bafilomycin) blocking autophagy increases oxidative stress. This finding, though clearly demonstrated, is hard to explain previous finding that autophagy increases (and with dysfunction) in PD. The authors can show status of autophagy in many of their experiment to increase the reliability of the data. Also, the authors should also treat the control cells, but not only show the baseline data of the control cells.

Figure 3 - The legend does not clearly explain the treatment of the cells. Are the responses or stress levels related to the severity of PD (IOPD or LOPD?) of these cells? Why stress decreases at 24h?

Figure 4 - Control cells should be treated. Arsenite, an oxidation agent, decreases GAA uptake; but may be the treatment is too harsh that it destroys cell functions.

Figure 5 - NAC enhancement of GAA uptake is quite impressive. But the confocal result is not compatible with western blot. Experiments concerning M6PR are confusing. The decreased GAA uptake is related to membranous receptor function, but other experiments are talking about intracellular M6PR.

Figure 6 - The receptor study, unfortunately, does not support the supreme action of NAC shown by other experiments.

Figure 7 - Again, the mouse ERT+drug doesn't demonstrate the supreme effect of NAC shown by other experiments.

Referee #3 (Comments on Novelty/Model System for Author):

The models used are adequate. The statistical methodology applied is not adequate, as t test is unsuited to analyze certain datasets.

Referee #3 (Remarks for Author):

The manuscript by Tarallo and colleagues explores the role of oxidative stress in the pathophysiology of Pompe disease and the response to enzyme replacement therapy (ERT).
The authors present several experiments in vitro and one experiment in Pompe mice in which they demonstrate that oxidative stress impairs uptake of recombinant human alpha acid glucosidase (rhGAA) and that antioxidant drugs enhance rhGAA uptake.
The work is highly relevant to the development of more effective adjuvant therapies for Pompe disease as it provides potentially simple therapeutic interventions. Notably, ERT in Pompe disease has several shortcomings, and even next generation ERTs being developed do not appear to

provide significant incremental benefit to patients.

Main comments:

Figure 4. The authors show the impact of oxidative stress on GAA uptake and conclude that treatment with arsenite impairs GAA uptake and lysosomal trafficking. While the result is potentially of great interest, the effect of arsenite on cell viability has not been evaluated, which may impact GAA uptake. Additionally, the results would be more convincing if a second agent inducing oxidative stress was used in the experiments.

Figure 6. The results presented are not convincing. First, it is unclear why the authors speculate that oxidative stress would affect M6PR abundance on the cell surface. This could easily be the result of autophagy blockage. One easy way to answer the question could be to treat Pompe fibroblasts with a compound enhancing autophagy (like rapamycin or torin) and compare the results with the ones obtained with antioxidants, which are not particularly strong. It would be better to use ANOVA for the statistical analysis of Fig. 6G (rather than t test).

Figure 7. The main hypothesis presented by the authors is that oxidative stress reduced efficacy of GAA, possibly by impairing uptake via M6PR. If this is the case, then it would be more appropriate to present Western blot data on the tissue collected from the animal treated with rhGAA to show uptake and particularly trafficking to the lysosome (cleaved forms of GAA). The measurement of GAA activity in tissues does not necessarily provide this information.

Most of the statistical analyses appear to be done with a t test, which is not appropriate to compare multiple treatment conditions in the same experiment.

It would strengthen the results to provide some data on glycogen clearance, in vitro or in vivo.

Discussion: it would be ideal to add a paragraph on the limitations of the study. Specifically, on the lack of efficacy data in vitro and in vivo (unless added to the manuscript) and on the fact that antioxidant therapies often times have failed to deliver clinically meaningful results in trials.

Referee #1

General:
A references list is missing. Thus, there was no possibility to cross check citations etc., which is usually a no go. Furthermore, there are many typos within the manuscript and graphical data presentation is poor. Do all authors read and controlled the final version of the manuscript, which is in their responsibility? However, since data and experiments are convincing in general and novel, the above referred concerns could be addressed within a major revision.

We apologize for the mistake in uploading the manuscript files. A reference list has been added. The text has been checked for typos and the graphical presentation of data improved.

Provide final concentrations for the antibodies instead of dilutions since dilutions are not representative and not appropriate. Since antibody concentrations differ from lot to lot, antibody concentrations used for western blots etc. need to be provided as Provide complete producer information for the anti-GAA antibody.

The final concentrations of the antibodies have been added in the section “Methods” (tables at page 17 and 20 of the revised manuscript).
Only for the anti-GAA antiserum, that is not commercial, this is information is not available. This is a polyclonal antiserum developed by our laboratory. This antiserum detects all GAA molecular isoforms and has been used in several previous studies (for example Parenti et al, Mol Ther. 2007, 15:508-14; Porto et al. Mol Ther. 2009, 17:964-71; Porto et al, Mol Ther. 2012, 20:2201-11).

Statistics:
Unfortunately applied statistics are wrong in most cases, which need to be revised and might have a major impact on the outcome.
If more than TWO columns are tested from ONE experiment, the Student's T test is NOT allowed. Instead, an ANOVA test with subsequent post-hoc analysis needs to be performed to correct for multiple testing. In detail, revise statistics for all experiments in Figure 2, Figure 4 A and C, Figure 5 A, B and D, Figure 6 B, C, G, Figure 7 C, Figure EV2 B, Figure EV3 A below; Figure EV7, Figure EV8 A. After using the appropriate statistical tool, authors might recognize that some outcomes are not significant any more. If a trend is visible, authors are free to perform additional experiments to raise the N. If not, data need to be re-evaluated and discussed.

The statistics has been thoroughly revised, according to the reviewer’s suggestion. Specifically, we have revised the statistical analysis in the following figures:
- Figure 2; (now includes also controls, see reviewer 2 comment);
- Figure 4A and D (C in the original manuscript; now the figure includes also controls, see reviewer 2 comment);
- Figure 5 A, B, F (previously D) and G (we have added a new panel);

- Figure 6 D (this figure has been revised and the original B and C panels are now in Figure EV6);
- Figure 7 C and D;
- Figure EV2 B (now Figure EV1B, C);
- Figure EV3 B (now Figure EV1E, F);
- Figure EV7 (now Figure EV4);
- Figure EV8 A (now Figure EV5A).

The statistical tests used are now indicated in each of the figure legends.

In all instances the statistical significance of our data has been confirmed, of course with different p-values.

Graphical layout: Current figures are looking very poor and do not correspond to the comprehensive experiments and convincing data, which is too bad. Please revise ALL figures and refrain from using poor standard Excel graphics. For instance, use GRAPHPAD Prism for charts. Furthermore, delete the alpha channel in the figures to get rid of the edges around some charts.

All figures and supplementary material have been revised, as suggested by the reviewer, using GRAPHPAD Prism 9.1.1. We thank the reviewer for this suggestion.

Referee #2

Figure 1/2 - the authors show that PD cells are with increased oxidative stress. Enhancement of autophagy (starvation, rapamycin) decrease the oxidative stress, while (Bafilomycin) blocking autophagy increases oxidative stress. This finding, though clearly demonstrated, is hard to explain previous finding that autophagy increases (and with dysfunction) in PD. The authors can show status of autophagy in many of their experiment to increase the reliability of the data.

The point raised by the reviewer is interesting. Actually, literature data support the idea that the autophagic defect in Pompe disease derives from dysregulation of this pathway at different levels with “a combination of induction of autophagy and autophagic block” (see Myerowitz R, et al. Impaired autophagy: The collateral damage of lysosomal storage disorders. EBioMedicine 2021; 63:103166). It is also clear that the autophagic pathway can be further manipulated. It has been suggested that “given that the block of autophagic flux appears to be a shared feature in many lysosomal storage diseases, the attempts to overcome/reduce this blockage by stimulating autophagy seem reasonable” (same review as above, Myerowitz et al, 2021). Indeed, there are studies in which the autophagic pathway was genetically or pharmacologically manipulated. For example:
- Lim JA, et al. (Therapeutic Benefit of Autophagy Modulation in Pompe Disease. Mol Ther 2018, 26:1783-1796);
- Spampanato C, et al. (Transcription factor EB is a new therapeutic target for Pompe disease. EMBO Mol Med 2013; 5:691-706), in which stimulation of autophagy through TFEB overexpression resulted in “increase lysosomal–autophagosomal fusion“;

- Lim JA, et al. (Modulation of mTOR signaling as a strategy for the treatment of Pompe disease. EMBO Mol Med 2017; 9:353-370), suggesting that “the aberrant mTOR signaling can be reversed by arginine”.
These studies concur with the concept that the autophagic pathway can be further stimulated, even if already upregulated, most likely leading to improved autophagosomal-lysosomal fusions.

According to the reviewer’s request, we have checked the effect of the autophagy-modulating treatments shown in figure 2 (starvation, rapamycin, MK6-83, bafilomycin) on autophagy status, through western blot analysis of the common markers LC3-I/II. Under baseline conditions three Pompe disease fibroblast cell lines showed increased LC3, compared to controls (consistent with previous data obtained in our lab, see Cardone M, et al. Abnormal mannose-6-phosphate receptor trafficking impairs recombinant alpha-glucosidase uptake in Pompe disease fibroblasts. Pathogenetics 2008; 1:6). Stimulation of autophagy in these cell lines, under the experimental conditions set in the original manuscript, resulted into a relative increase of LC3-II, while bafilomycin induced remarkable accumulation LC3-II, consistent with a complete block of the pathway and further accumulation of undegraded LC3-II. These data are now included in Figure 2A and B and in Figure EV2C.

We now discuss that the combination of literature-derived information and of the results of our experiments indicates that the autophagy flux is impaired in Pompe disease but not totally blocked (section “Results”, page 8, lines 16-21). Even if upregulated, the autophagic pathway is still sensitive to pharmacological or physiological stimuli and can be further manipulated and activated. While substrates (such as glycogen) cannot be degraded in lysosomes, due the enzymatic defect, ROS can be better disposed of upon stimulation of autophagy.

The experiments shown in figure 2 are those in which it is most important and appropriate to look at effects of treatments on autophagy status. For sake completeness, we also looked at the effects of the other treatments included in our manuscript on the autophagy status. Specifically, we now show the effects of sodium arsenite, TBP and antioxidants (Figure EV2D, E); page 9 lines, 20-23; page 11, lines 7-9). The results of these experiments are consistent with literature data (references have been added).
All data concerning the autophagy status under different conditions are summarized in a single figure as expanded view material (Figure EV2).

Figure 1/2. Also, the authors should also treat the control cells, but not only show the baseline data of the control cells.

Figures 2 and 4B have been modified according to the reviewer’s comment. Figure 2 now includes the effects of pharmacological manipulation of autophagy in control cells. As expected, in controls, in which baseline stress levels are normal, activation of autophagy showed only minor effects (although with a similar trend compared to Pompe cells). In contrast, bafilomycin-mediated block of autophagy in controls induced evident consequences, with substantial changes in terms of increased ROS and lipid peroxidation, and reduction of GSH (see section “Results”, page 8, lines 7-11). These results appear plausible, given the normal baseline status of autophagy in controls.

We also included the effects of sodium arsenite treatment and tert-butyl-peroxide in controls in Figure 4B. Both treatments resulted in perturbation of stress indicators in these cells, with significant increases of ROS and lipid peroxidation, and reduction of GSH.

Figure 3 - The legend does not clearly explain the treatment of the cells. Are the responses or stress levels related to the severity of PD (IOPD or LOPD?) of these cells?

Yes, apparently IOPD cells internalize rhGAA less efficiently than LOPD. In the original manuscript this information could be inferred by comparing the GAA activity data with table 1. We have now made this information more explicit with a short comment in the text (See section Results, page 8, last two lines).

Why stress decreases at 24h?

Actually, stress does not decrease at 24 hrs. Please, notice that the scales of the two panels are different (due to the substantially higher increments in GAA activity at 24 hrs, compared to 4 hrs).

Figure 4 - Control cells should be treated.

According to the reviewer's suggestion we now provide data in controls (Figure 4B). As expected, arsenite treatment in controls increases stress levels (page 9, lines 18-20).
We did not measure the effects of arsenite on the uptake of rhGAA in controls. In general, we do not measure rhGAA uptake in controls because of the presence of normal endogenous GAA. This has been our approach in our previous papers on pharmacological chaperones and ERT (for example: Porto C, et al. The pharmacological chaperone N-butyldeoxynojirimycin enhances enzyme replacement therapy in Pompe disease fibroblasts. Mol Ther. 2009 17:964-71; Porto C, et al. Pharmacological enhancement of α-glucosidase by the allosteric chaperone N-acetylcysteine. Mol Ther. 2012 20:2201-11). We never had comments on this approach by reviewers. Actually, the results in controls would be difficult to interpret and confounding, possibly with a combination of effects of arsenite not only on uptake but also on the synthesis/trafficking of the endogenous enzyme.
Please notice that the effects of a second oxidating agent, tert-butyl-peroxide, have been added in Figure 4.

Figure 4 - Arsenite, an oxidation agent, decreases GAA uptake; but may be the treatment is too harsh that it destroys cell functions.

We have added information on the effect of arsenite treatments on cell viability at different time points and concentrations (Figure 4A) (also for another stress-inducing agent, tert-butyl-peroxide, see reviewer 3 comments). We used a validated test (MTT

assay, 3-(4,5-dimethylthiazol-2-yl)-2,5-diphenyltetrazolium bromide) to study cell viability and cytotoxicity. This test showed that 100 μM arsenite treatment for up to 4 hours does not affect cell viability and that at 6 hours (the condition used in our experiments) cell viability is still acceptable (>50%). Clear effects of arsenite treatment on GAA activity also detectable at shorter time points (4 hours at 100 μM) and lower arsenite concentrations (30 μM at 6 hours) at which there was no evident toxicity by MTT test (See Appendix Figure 3). This indicates that the effect on GAA activity correction is not due to cell toxicity.
These data are now discussed in the section “Results” (page 9, lines10-17).

Figure 5 - NAC enhancement of GAA uptake is quite impressive. But the confocal result is not compatible with western blot.

The confocal IF images shown in Figure 5D in the original manuscript are those obtained in one of the three cell lines studied (PD3). The results obtained in the other two cell lines were shown in EV7 (now Figure EV4).
We agree with the reviewer that the images shown in the original manuscript poorly represent the quantitative analysis (Figure 5D, right) and may be confusing for the reader.
However, the quantitative analysis of immunofluorescence experiments data (that combines the analyses performed in all three cell lines and is the most important information concerning rhGAA-LAMP2 co-localization, see panel G) is consistent with the results of GAA activity and western blot, confirming that NAC is clearly the most effective drug.
Thus, for sake of clarity and to address the reviewer’s concerns, we have reassembled Figure 5 (with data already available in the first version of the manuscript), showing in panel 5E the results obtained in patient PD1 that are more representative of the quantitative analysis of all cells, and are more consistent with the western blot data. The revised version of figure 5, now includes the IF images (panel 5E) obtained in PD1, the quantitative analysis of PD1 (Figure 5F) and the quantitative analysis made by combining the data obtained in all cell lines (Figure 5G). The supplemental material EV7 includes the images in PD2 and PD3, and the quantitative analysis in the individual these cell lines.
Please, notice that in this case we have not performed new experiments but only reassembled the figures, already available and informative, that were in the original manuscript.

Experiments concerning M6PR are confusing. The decreased GAA uptake is related to membranous receptor function, but other experiments are talking about intracellular M6PR.

We agree with the reviewer that the main information in Figure 6 relates to the amounts of M6PR available at the plasma membrane and to the changes induced by antioxidant treatment.
However, after internal discussion (particularly with our co-authors that are more qualified in cell biology), we decided also to show the total amount of M6PR in cells and

at the trans-Golgi, because we thought that this information could be useful and because we expected that readers would ask also for it.
To meet the reviewer's request, we have moved panels A, B and C to the supplemental material (Figure Appendix 4). We believe that, by rearranging the figure in this way, on one hand the reading of the paper is more straightforward, while on the other hand the information remains available for those who would like to know it.

Figure 6 - The receptor study, unfortunately, does not support the supreme action of NAC shown by other experiments.

The enhancement of M6PR availability at the plasma membrane is probably only one of different mechanisms (for example vesicle trafficking, vesicle membrane composition, etc). This was already clearly stated in the discussion in the original manuscript and is now further emphasized in the paragraph on the limitations of the study, as requested by reviewer 3.
In addition to that, as clearly indicated in the original manuscript (section Discussion page 13, lines 5-8 from top; now page 14, lines 18 and following), NAC is not only an antioxidant but has also a chaperone effect directly on rhGAA (see Porto C, et al. Pharmacological enhancement of α-glucosidase by the allosteric chaperone N-acetylcysteine. Mol Ther 2012; 20:2201-11). This effect is probably in part mediated by reduction of an oxidized cysteine at position 938 (see Roig-Zamboni et al. Structure of human lysosomal acid α-glucosidase-a guide for the treatment of Pompe disease. Nat Commun 2017; 8:11112017). Thus, the remarkable effect of NAC in vitro is the results of different and combined modes of action.
In our opinion this is sufficient to explain the apparent discrepancy between the data on NAC effect on GAA activity, rhGAA processing, rhGAA lysosomal trafficking, and the data on M6PR localization. We have slightly modified the discussion to make these comments more explicit and clearer (page 14, lines 23-26).

Figure 7 - Again, the mouse ERT+drug doesn't demonstrate the supreme effect of NAC shown by other experiments.

We believe that is extremely difficult to compare results obtained *in vitro* (under experimental conditions in which there is full and prolonged exposure to drugs, without major physiological or anatomical barriers, without drug metabolism) with *in vivo* experiments, in which many factors impact on the efficacy of drugs (intestinal absorption, barriers, bioavailability, pharmacokinetics, drug metabolism in liver and other organs, etc).
We agree with the reviewer that discussing these concepts would be useful for the readers, and with reviewer 3 (see below) that a paragraph on the limitations of our study would be of help. Thus, these concepts are now discussed in further detail in the discussion (page 15, last paragraph).

Referee #3

Figure 4. The authors show the impact of oxidative stress on GAA uptake and conclude that treatment with arsenite impairs GAA uptake and lysosomal trafficking. While the result is potentially of great interest, the effect of arsenite on cell viability has not been evaluated, which may impact GAA uptake. Additionally, the results would be more convincing if a second agent inducing oxidative stress was used in the experiments.

Data on the effect of arsenite on cell viability are now available in figure 4 (see response to reviewer 2).
According to the reviewer's suggestions we have also tested the effect of another stress-inducing agent (tert-butyl-peroxide, TBP) at concentrations (10 μM) that do not significantly impact on cell viability (Figure 4A). Similarly to arsenite, incubation of control and Pompe disease cells with TBP induced stress (Figure 4B) and resulted into reduced correction of GAA activity by rhGAA, and into impaired processing of the recombinant enzyme (Figure 4C- F).

Figure 6. The results presented are not convincing. First, it is unclear why the authors speculate that oxidative stress would affect M6PR abundance on the cell surface. This could easily be the result of autophagy blockage. One easy way to answer the question could be to treat Pompe fibroblasts with a compound enhancing autophagy (like rapamycin or torin) and compare the results with the ones obtained with antioxidants, which are not particularly strong.

As indicated in the original manuscript, M6PR is one of the most important players in the uptake and trafficking of recombinant lysosomal enzymes. In addition, we know from previous work that M6PR is mislocalized in Pome disease fibroblasts and is less abundant at the plasma membrane (Cardone M, et al. Abnormal mannose-6-phosphate receptor trafficking impairs recombinant alpha-glucosidase uptake in Pompe disease fibroblasts. Pathogenetics 2008; 1:6). Therefore, we looked at M6PR as one of the possible mechanisms involved in the defective correction of GAA activity and at the effects of antioxidants on M6PR. We are aware that there might be other factors, for example the impaired vesicle trafficking through the endocytic pathway (see references mentioned in the introduction) that impact on rhGAA uptake and GAA activity correction.
We believe that these aspects are sufficiently discussed in the sections "Results" (page 11, lines 17-21) and "Discussion" (page 14, last paragraph and page 15, first paragraph).

As suggested by the reviewer, we have also tested the effect of rapamycin and torin. The results are now provided as supplementary material (Figure EV5C, D). Rapamycin induced some improvement of M6PR amounts at the plasma membrane of Pompe disease fibroblasts, although to a lower extent compared with antioxidants. This slight enhancement is in line with the results already shown in Figure EV5A (EV8 in the previous version of the manuscript), indicating that also stimulation of autophagy improves correction of GAA activity by rhGAA. We may speculate that both correction of stress and stimulation of autophagy improve correction of GAA activity. This is not surprising as stimulation of autophagy by itself reduces stress (see Figure 2) and since it is known that autophagy enhancement (by TFEB overexpression) results into accelerated vesicle trafficking in Pompe cells (see Spampanato C, et al. Transcription

factor EB is a new therapeutic target for Pompe disease. EMBO Mol Med 2013; 5:691-706). As antioxidant drugs are widely used in human therapy and show in general good safety profiles, their use would advantageous compared to autophagy enhancers.
We now briefly discuss these aspects in the section “Results” (page 12, second paragraph) and in the section “Discussion” (page 15, lines 22-24).

It would be better to use ANOVA for the statistical analysis of Fig. 6G (rather than t test).

This has been done (see also comments of reviewer 1).

Figure 7. The main hypothesis presented by the authors is that oxidative stress reduced efficacy of GAA, possibly by impairing uptake via M6PR. If this is the case, then it would be more appropriate to present Western blot data on the tissue collected from the animal treated with rhGAA to show uptake and particularly trafficking to the lysosome (cleaved forms of GAA). The measurement of GAA activity in tissues does not necessarily provide this information.

We thank the reviewer for this suggestion. Figure 7 E, F now shows the results of GAA western blot analysis in three mice (the samples were already available from the original experiments). Western blot images have been quantitatively analyzed and appear in general consistent (although not statistically significant) with the results of the enzymatic assays, with improved amounts of mature GAA in mice treated with the combination of ERT and antioxidants, compared to ERT alone.

Most of the statistical analyses appear to be done with a t test, which is not appropriate to compare multiple treatment conditions in the same experiment.

The statistics analysis has been thoroughly revised (see response to reviewer 1). All figures have been revised.

It would strengthen the results to provide some data on glycogen clearance, in vitro or in vivo.

We have performed a glycogen assay in relevant tissues (heart, muscles) from mice treated with ERT alone or in combination with NAC or idebenone. Tissue homogenates, deriving from the original experiments on GAA activity, were already available in our lab.
We observed a general trend towards decrease in glycogen in tissues from animals treated with NAC and ERT, compared to those treated with ERT alone, that reached statistical significance in quadriceps (Figure 7H). We did not observe significant changes with ERT-idebenone co-administration. (Figure 7H). These results should be interpreted considering the slight superiority of NAC *in vivo* and the fact that it is plausible and expected that a single injection of rhGAA is not sufficient to cause detectable changes in substrate storage. Long-term studies for further pre-clinical development of a

combination treatment may address this point. The scope of the present work is only to provide a proof of principle that secondary dysregulation of cellular functions and pathways are potential targets of therapy.

Discussion: it would be ideal to add a paragraph on the limitations of the study. Specifically, on the lack of efficacy data in vitro and in vivo (unless added to the manuscript) and on the fact that antioxidant therapies often times have failed to deliver clinically meaningful results in trials.

Yes, we agree with the reviewer, thanks for the suggestion, this would make the manuscript more comprehensible and clear. We have introduced a paragraph in the section "Discussion" (page 15, last paragraph).
We agree that antioxidants have failed to provide clinically meaningful results in trials, but the focus of our work is the synergy with ERT (and possibly with other therapeutic approaches), not the effects of these drugs alone.

1st Revision - Editorial Decision 13th Aug 2021

13th Aug 2021

Dear Prof. Parenti,

Thank you for the submission of your revised manuscript to EMBO Molecular Medicine. I am pleased to inform you that we will be able to accept your manuscript pending the following final amendments:

1) Please address all the concerns raised by the referees. Particular attention should be given to manuscript preparation (text and figures) i.e in Figure 3 label E needs adjusting/aligning to the corresponding figure panel. Please provide representative western blot images and describe in M&M the method you used to quantify them particularly regarding curved and unevenly loaded western blots.
2) In the main manuscript file, please do the following:
- Correct/answer the track changes suggested by our data editors by working from the attached document.
- Please clarify whether Maria Antonietta De Matteis in the manuscript and Antonella De Matteis in our submission system is the same person and use the correct name.
- For author contributions please use initials.
- In M&M, include a statement that informed consent from patients was obtained and that the experiments conformed to the principles set out in the WMA Declaration of Helsinki and the Department of Health and Human Services Belmont Report.
- Add title/short legend to Table 1. Please fuse antibody tables to Table 2, add title/short legend, move it to the end of the manuscript and call it out at appropriate po on M&M.
3) Synopsis:
- Synopsis text: Please submit synopsis text as a separate .doc file. Please check your synopsis text, revise it if necessary and submit its final versions with your revised manuscript. Please be aware that in the proof stage minor corrections only are allowed (e.g., typos).
- Synopsis image: Please provide a striking image or visual abstract as a high-resolution jpeg file 550 px-wide x (250-400)-px high to illustrate your article.
4) Funding: Please make sure that information about all sources of funding are complete in both our submission system and in the manuscript
5) As part of the EMBO Publications transparent editorial process initiative (see our Editorial at http://embomolmed.embopress.org/content/2/9/329), EMBO Molecular Medicine will publish online a Review Process File (RPF) to accompany accepted manuscripts. This file will be published in conjunction with your paper and will include the anonymous referee reports, your point-by-point response and all pertinent correspondence relating to the manuscript. Let us know whether you agree with the publication of the RPF and as here, if you want to remove or not any figures from it prior to publication. Please note that the Authors checklist will be published at the end of the RPF.
6) Please provide a point-by-point letter INCLUDING my comments as well as the reviewer's reports and your detailed responses (as Word file).

I look forward to reading a new revised version of your manuscript as soon as possible.

Yours sincerely,

Zeljko Durdevic

Zeljko Durdevic
Editor
EMBO Molecular Medicine

***** Reviewer's comments *****

Referee #1 (Remarks for Author):

This is a revised version of a previously submitted manuscript.
During the revision, the authors revised the statistic part and calculations as requested. However, the major concerns that raiased from the first version was a lack of accurateness, which was reflected by a missing reference list, many typos as well as questinable western blots. Although a refernce list is now provided and most typos have been corrected, the authors again failed to present a accurate final version of their manuscript. Within the revised version of the manuscript the first 3 figures are missing. Similar to the initial submission final approval of the manuscript wasn't successful.
Furthermore, western blots in figure 1 f, 2 a, 4 d and 5 c are creepy and do not allow a proper quantification, espacially if GAA isoforms are analyzed. If these were the best blots the reader needs to ask himself how poor were the others. This is not the quality usually published in EMBO.

Referee #2 (Remarks for Author):

The authors have responded to my questions and provided additional positive data. However, the manuscript preparation is still poor, tracking is not removed, and the combined pdf is broken. For data, gel loading was often uneven and curved, and quantification sometimes don't look reliable.

Referee #3 (Comments on Novelty/Model System for Author):

The manuscript expands upon earlier findings on autophagy and mitophagy impairment in Pompe disease and provides solid data in vitro and in vivo models of the disease demonstrating that modulation of oxidative stress has a potential impact on the efficacy of enzyme replacement therapies. The manuscript also provides possible mechanistic insights on how the modulation of oxidative stress would affect efficacy of GAA replacement, i.e. via upregulation of M6PR at the surface of affected tissues.

Referee #3 (Remarks for Author):

The revised version of the manuscript is substantially improved and addressed the issues initially raised to the attention of the authors. The work is solid and nicely presented and is of potential interest of the Pompe community.

The authors performed the requested editorial changes.

***** Reviewer's comments *****

Referee #1 (Remarks for Author):

This is a revised version of a previously submitted manuscript.
During the revision, the authors revised the statistic part and calculations as requested.
However, the major concerns that raiased from the first version was a lack of accurateness, which was reflected by a missing reference list, many typos as well as questinable western blots. Although a refernce list is now provided and most typos have been corrected, the authors again failed to present a accurate final version of their manuscript. Within the revised version of the manuscript the first 3 figures are missing. Similar to the initial submission final approval of the manuscript wasn't successful.

I apologize for all inaccuracies in the previous version of manuscript, particularly in the section “Materials and Methods”. The whole text of the current version has been revised.
The point concerning the first 3 figures is addressed in the cover letter.

Furthermore, western blots in figure 1 f, 2 a, 4 d, and 5 c are creepy and do not allow a proper

quantification, espacially if GAA isoforms are analyzed. If these were the best blots the reader needs to ask himself how poor were the others. This is not the quality usually published in EMBO.

As stated above we have added new images, even though we believe that the results of western blots already provided in the previous version were clear, were in line with common standards in many published papers, and allow for reliable quantification using standard tools.
We provide new images for the western blots shown in Figure 1F and Figure 2A. We hope this is sufficient to address reviewer 1 concerns. In Fig. 4D the bands corresponding to GAA isoforms are straight and the pattern is consistent with the common pattern reported in many papers from the literature (to mention a few: Bijvoet et al, 1998, Hum Mol Genet, 7:1815; van den Hout et al, 2004, Pediatrics, 113:448; Bali et al. Am J Med Genet C Semin Med Genet. 2012 160C:40; Nilsson et al, 2014, Gene. 537:41; Wang et al, 2014, Mol Genet Metab. 111:92; Khanna et al, 2014, Plos One, 9:e102092, etc) and in commercial antibody brochures (for example: https://www.rndsystems.com/products/human-lysosomal-alpha-glucosidase-antibody-2489c_mab8329). The same is true for Figure 5C. We would also like to mention that in this figure, given the major effect of NAC on GAA stability and processing, the lane corresponding to the treatment with rhGAA + NAC may appear overexposed. However, this is the only exposure that allows for visualization of GAA isoforms in fibroblasts treated with rhGAA alone (relatively faint, compared to those treated with rhGAA + NAC). The quantitative analysis is based on different exposures of the same blot that consistently show the same results.
In the section "Materials and Methods we now mention the software that was used to capture images and for band densitometric quantification (pages 17-18).

Referee #2 (Remarks for Author):

The authors have responded to my questions and provided additional positive data. However, the manuscript preparation is still poor, tracking is not removed, and the combined pdf is broken. For data, gel loading was often uneven and curved, and quantification sometimes don't look reliable.

See responses to the editor and to reviewer 2.

Referee #3 (Comments on Novelty/Model System for Author):

The manuscript expands upon earlier findings on autophagy and mitophagy impairment in Pompe disease and provides solid data in vitro and in vivo models of the disease demonstrating that modulation of oxidative stress has a potential impact on the efficacy of enzyme replacement therapies. The manuscript also provides possible mechanistic insights on how the modulation of oxidative stress would affect efficacy of GAA replacement, i.e. via upregulation of M6PR at the surface of affected tissues.

Referee #3 (Remarks for Author):

The revised version of the manuscript is substantially improved and addressed the issues initially raised to the attention of the authors. The work is solid and nicely presented and is of potential interest of the Pompe community.

No response required.

We are pleased to inform you that your manuscript is accepted for publication and is now being sent to our publisher to be included in the next available issue of EMBO Molecular Medicine.

EMBO PRESS

YOU MUST COMPLETE ALL CELLS WITH A PINK BACKGROUND ↓

PLEASE NOTE THAT THIS CHECKLIST WILL BE PUBLISHED ALONGSIDE YOUR PAPER

Corresponding Author Name: Giancarlo Parenti
Journal Submitted to: EMBO Molecular Medicine
Manuscript Number: EMM-2021-14434

Reporting Checklist For Life Sciences Articles (Rev. June 2017)

This checklist is used to ensure good reporting standards and to improve the reproducibility of published results. These guidelines are consistent with the Principles and Guidelines for Reporting Preclinical Research issued by the NIH in 2014. Please follow the journal's authorship guidelines in preparing your manuscript.

A- Figures
1. Data
The data shown in figures should satisfy the following conditions:

- the data were obtained and processed according to the field's best practice and are presented to reflect the results of the experiments in an accurate and unbiased manner.
- figure panels include only data points, measurements or observations that can be compared to each other in a scientifically meaningful way.
- graphs include clearly labeled error bars for independent experiments and sample sizes. Unless justified, error bars should not be shown for technical replicates.
- if n< 5, the individual data points from each experiment should be plotted and any statistical test employed should be justified
- Source Data should be included to report the data underlying graphs. Please follow the guidelines set out in the author ship guidelines on Data Presentation.

2. Captions
Each figure caption should contain the following information, for each panel where they are relevant:

- a specification of the experimental system investigated (eg cell line, species name).
- the assay(s) and method(s) used to carry out the reported observations and measurements
- an explicit mention of the biological and chemical entity(ies) that are being measured.
- an explicit mention of the biological and chemical entity(ies) that are altered/varied/perturbed in a controlled manner.

- the exact sample size (n) for each experimental group/condition, given as a number, not a range;
- a description of the sample collection allowing the reader to understand whether the samples represent technical or biological replicates (including how many animals, litters, cultures, etc.).
- a statement of how many times the experiment shown was independently replicated in the laboratory.
- definitions of statistical methods and measures:
  - common tests, such as t-test (please specify whether paired vs. unpaired), simple χ2 tests, Wilcoxon and Mann-Whitney tests, can be unambiguously identified by name only, but more complex techniques should be described in the methods section;
  - are tests one-sided or two-sided?
  - are there adjustments for multiple comparisons?
  - exact statistical test results, e.g., P values = x but not P values < x;
  - definition of 'center values' as median or average;
  - definition of error bars as s.d. or s.e.m.

Any descriptions too long for the figure legend should be included in the methods section and/or with the source data.

In the pink boxes below, please ensure that the answers to the following questions are reported in the manuscript itself. Every question should be answered. If the question is not relevant to your research, please write NA (non applicable). We encourage you to include a specific subsection in the methods section for statistics, reagents, animal models and human subjects.

USEFUL LINKS FOR COMPLETING THIS FORM

http://www.antibodypedia.com
http://1degreebio.org
http://www.equator-network.org/reporting-guidelines/improving-bioscience-research-repor
http://grants.nih.gov/grants/olaw/olaw.htm
http://www.mrc.ac.uk/Ourresearch/Ethicsresearchguidance/Useofanimals/index.htm
http://ClinicalTrials.gov
http://www.consort-statement.org
http://www.consort-statement.org/checklists/view/32-consort/66-title
http://www.equator-network.org/reporting-guidelines/reporting-recommendations-for-tumo
http://datadryad.org
http://figshare.com
http://www.ncbi.nlm.nih.gov/gap
http://www.ebi.ac.uk/ega
http://biomodels.net/
http://biomodels.net/miriam/
http://jjj.biochem.sun.ac.za
https://osp.od.nih.gov/biosafety-biosecurity-and-emerging-biotechnology/
http://www.selectagents.gov/

B- Statistics and general methods

Please fill out these boxes ↓ (Do not worry if you cannot see all your text once you press return)

1.a. How was the sample size chosen to ensure adequate power to detect a pre-specified effect size?	For in vitro studies we used cell lines available at our cell bank. Firoblasts and myoblast cultures had been obtained for diagnostic purposes. We chose not to perform new biopsies (that require invasive procedures potentially causing discomfort to patients) to increase sample size.
1.b. For animal studies, include a statement about sample size estimate even if no statistical methods were used.	Sample size was in line with previous studies performed in our laboratory
2. Describe inclusion/exclusion criteria if samples or animals were excluded from the analysis. Were the criteria pre-established?	We only used male animals, in which the PD phenotype is fully expressed
3. Were any steps taken to minimize the effects of subjective bias when allocating animals/samples to treatment (e.g. randomization procedure)? If yes, please describe.	In in vivo studies we used biochemical tests or western blot analysis, that are not subject to subjective biases
For animal studies, include a statement about randomization even if no randomization was used.	No randomization used
4.a. Were any steps taken to minimize the effects of subjective bias during group allocation or/and when assessing results (e.g. blinding of the investigator)? If yes please describe.	NA
4.b. For animal studies, include a statement about blinding even if no blinding was done	No blinding
5. For every figure, are statistical tests justified as appropriate?	Yes. The reviewers' concerns about statistical methods have been addressed in the revised version (see rebuttal)
Do the data meet the assumptions of the tests (e.g., normal distribution)? Describe any methods used to assess it.	NA
Is there an estimate of variation within each group of data?	No

Is the variance similar between the groups that are being statistically compared?	NA

C- Reagents

6. To show that antibodies were profiled for use in the system under study (assay and species), provide a citation, catalog number and/or clone number, supplementary information or reference to an antibody validation profile. e.g., Antibodypedia (see link list at top right), 1DegreeBio (see link list at top right).	Provided in the text (materials and methods)
7. Identify the source of cell lines and report if they were recently authenticated (e.g., by STR profiling) and tested for mycoplasma contamination.	Cells were available at our biobank. Cells are periodically tested for mycoplasma contamination

* for all hyperlinks, please see the table at the top right of the document

D- Animal Models

8. Report species, strain, gender, age of animals and genetic modification status where applicable. Please detail housing and husbandry conditions and the source of animals.	A KO PD mouse model obtained by insertion of neo into the GAA gene exon 6 (Raben et al, 1998) was purchased from Charles River Laboratories (Wilmington, MA), and is currently maintained at the Cardarelli Hospital's Animal Facility (Naples, Italy) and TIGEM Animal Facility (Pozzuoli, Italy).
9. For experiments involving live vertebrates, include a statement of compliance with ethical regulations and identify the committee(s) approving the experiments.	Animal studies were performed according to the EU Directive 86/609, regarding the protection of animals used for experimental purposes (IACUC project n°523/2015-PR approved by the Italian Ministry of Health). Every procedure on the mice were performed with the aim of ensuring that discomfort, distress, pain, and injury would be minimal. Mice were euthanized following anesthesia.
10. We recommend consulting the ARRIVE guidelines (see link list at top right) (PLoS Biol. 8(6), e1000412, 2010) to ensure that other relevant aspects of animal studies are adequately reported. See author guidelines, under 'Reporting Guidelines'. See also: NIH (see link list at top right) and MRC (see link list at top right) recommendations. Please confirm compliance.	The studies have been performed in compliance with guidelines

E- Human Subjects

11. Identify the committee(s) approving the study protocol.	NA
12. Include a statement confirming that informed consent was obtained from all subjects and that the experiments conformed to the principles set out in the WMA Declaration of Helsinki and the Department of Health and Human Services Belmont Report.	NA
13. For publication of patient photos, include a statement confirming that consent to publish was obtained.	NA
14. Report any restrictions on the availability (and/or on the use) of human data or samples.	NA
15. Report the clinical trial registration number (at ClinicalTrials.gov or equivalent), where applicable.	NA
16. For phase II and III randomized controlled trials, please refer to the CONSORT flow diagram (see link list at top right) and submit the CONSORT checklist (see link list at top right) with your submission. See author guidelines, under 'Reporting Guidelines'. Please confirm you have submitted this list.	NA
17. For tumor marker prognostic studies, we recommend that you follow the REMARK reporting guidelines (see link list at top right). See author guidelines, under 'Reporting Guidelines'. Please confirm you have followed these guidelines.	NA

F- Data Accessibility

18: Provide a "Data Availability" section at the end of the Materials & Methods, listing the accession codes for data generated in this study and deposited in a public database (e.g. RNA-Seq data: Gene Expression Omnibus GSE39462, Proteomics data: PRIDE PXD000208 etc.) Please refer to our author guidelines for 'Data Deposition'.<br><br>Data deposition in a public repository is mandatory for:<br>a. Protein, DNA and RNA sequences<br>b. Macromolecular structures<br>c. Crystallographic data for small molecules<br>d. Functional genomics data<br>e. Proteomics and molecular interactions	NA
19. Deposition is strongly recommended for any datasets that are central and integral to the study; please consider the journal's data policy. If no structured public repository exists for a given data type, we encourage the provision of datasets in the manuscript as a Supplementary Document (see author guidelines under 'Expanded View' or in unstructured repositories such as Dryad (see link list at top right) or Figshare (see link list at top right).	NA
20. Access to human clinical and genomic datasets should be provided with as few restrictions as possible while respecting ethical obligations to the patients and relevant medical and legal issues. If practically possible and compatible with the individual consent agreement used in the study, such data should be deposited in one of the major public access-controlled repositories such as dbGAP (see link list at top right) or EGA (see link list at top right).	NA
21. Computational models that are central and integral to a study should be shared without restrictions and provided in a machine-readable form. The relevant accession numbers or links should be provided. When possible, standardized format (SBML, CellML) should be used instead of scripts (e.g. MATLAB). Authors are strongly encouraged to follow the MIRIAM guidelines (see link list at top right) and deposit their model in a public database such as Biomodels (see link list at top right) or JWS Online (see link list at top right). If computer source code is provided with the paper, it should be deposited in a public repository or included in supplementary information.	NA

G- Dual use research of concern

22. Could your study fall under dual use research restrictions? Please check biosecurity documents (see link list at top right) and list of select agents and toxins (APHIS/CDC) (see link list at top right). According to our biosecurity guidelines, provide a statement only if it could.	NA
